# Signature reversion of three disease‐associated gene signatures prioritizes cancer drug repurposing candidates

**DOI:** 10.1002/2211-5463.13796

**Published:** 2024-03-26

**Authors:** Jennifer L. Fisher, Elizabeth J. Wilk, Vishal H. Oza, Sam E. Gary, Timothy C. Howton, Victoria L. Flanary, Amanda D. Clark, Anita B. Hjelmeland, Brittany N. Lasseigne

**Affiliations:** ^1^ Department of Cell, Developmental and Integrative Biology, Heersink School of Medicine The University of Alabama at Birmingham AL USA

**Keywords:** cancer, drug repurposing, gene signature, glioblastoma, transcriptomic signature

## Abstract

Drug repurposing is promising because approving a drug for a new indication requires fewer resources than approving a new drug. Signature reversion detects drug perturbations most inversely related to the disease‐associated gene signature to identify drugs that may reverse that signature. We assessed the performance and biological relevance of three approaches for constructing disease‐associated gene signatures (i.e., limma, DESeq2, and MultiPLIER) and prioritized the resulting drug repurposing candidates for four low‐survival human cancers. Our results were enriched for candidates that had been used in clinical trials or performed well in the PRISM drug screen. Additionally, we found that pamidronate and nimodipine, drugs predicted to be efficacious against the brain tumor glioblastoma (GBM), inhibited the growth of a GBM cell line and cells isolated from a patient‐derived xenograft (PDX). Our results demonstrate that by applying multiple disease‐associated gene signature methods, we prioritized several drug repurposing candidates for low‐survival cancers.

## Graphical abstract

Signature reversion can be used for drug repurposing by identifying drug perturbations most inversely related to disease‐associated signatures. We applied three methods for disease‐associated signature construction to prioritize drug repurposing candidates for low‐survival cancers including pamidronate for GBM, which we further show decreases cell viability *in vitro* for two GBM cell culture models.

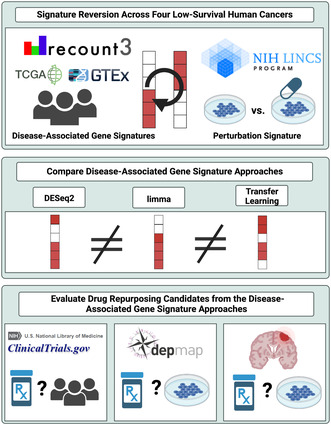

**Abbreviations**
CORUM: The Comprehensive Resource of Mammalian Protein Complexes
CYP450: cytochrome P450
DepMap: Cancer Dependency Map
FDR: false discovery rate
GBM: glioblastoma
GO: Gene Ontology
GTEx: Genotype‐Tissue Expression
LIHC: liver hepatocellular carcinoma
LINCS: The Library of Integrated Network‐based Cellular Signatures
LOF: loss‐of‐function
LUAD: lung adenocarinoma
MET: MET proto‐oncogene
MOA: mechanism of action
NCS: normalized connectivity score
PAAD: pancreatic adenocarcinoma
PCA: principal component analysis
PDX: patient‐derived xenograft
PLIER: pathway‐level information extractor
PPI: protein–protein interaction
PRISM: Profiling Relative Inhibition Simultaneously in Mixtures
RET: RET proto‐oncogene
SRA: Sequence Read Archive
STR: short tandem repeat
TCGA: The Cancer Genome Atlas
TPM: transcript per million
VEGFR‐2: vascular endothelial growth factor receptor 2
VST: variance stabilizing transformation

New therapeutic options are critical for improving cancer survival rates [1]. While novel drug discovery can be effective, developing these new drugs costs billions of US dollars on average and can take upwards of 13 years for FDA approval [2, 3]. Additionally, 95% of phase I clinical trial oncology drug candidates are ultimately not approved due to toxicity or inefficacy [2, 4]. However, drug repurposing (identifying novel indications for previously approved drugs) is a promising alternative to drug discovery. As the candidates have already passed toxicity testing in previous clinical trials, drug repurposing reduces both the time and cost needed for approval for a new indication [5, 6]. This approach successfully identified oncology and non‐oncology drugs for novel cancer applications. For example, imatinib, a tyrosine kinase inhibitor for chronic myeloid leukemia, has been repurposed for treating gastrointestinal stromal tumors, and rapamycin, originally used as an immunosuppressant for kidney transplants, was repurposed for the treatment of renal cell carcinoma [2, 7, 8].

Identifying prioritized drugs for future cancer clinical trials requires identifying both the drug target(s) associated with the disease process and the drug predicted to perturb those target(s). Toward those goals, several computational drug repurposing methods were developed that leverage optimized algorithms and high‐performance computing systems to prioritize novel drug repurposing candidates more quickly and with less expense than exhaustive large‐scale experimental approaches like phenotypic drug screens [5]. One such approach is signature reversion, a computational drug repurposing method used to prioritize drug repurposing candidates for the treatment of several cancers that were successfully validated in cell culture systems or mouse xenograft models [9, 10, 11, 12]. Signature reversion identifies drugs predicted to reverse disease‐associated gene signatures (i.e., gene expression differences between disease and control tissue) by determining which cell line drug perturbation signatures (i.e., gene expression differences before and after drug treatment) are most inverse from the disease‐associated gene signature [13, 14, 15]. While previous studies have investigated how signature reversion methods perform [9, 11, 16, 17, 18], understanding the impact of different approaches for selecting a disease‐associated gene signature on downstream signature reversion is critical for further candidate prioritization and interpretation to advance drug repurposing studies [11].

Here we applied and evaluated three approaches for selecting a disease‐associated gene signature for downstream signature reversion (Fig. S1). Two approaches involved developing a disease‐associated gene signature from differential gene expression analysis using limma and DESeq2, respectively [19, 20]. Limma and DESeq2 consistently perform well in differential gene expression analysis evaluations, incorporate covariates to reduce technical influences such as batch effects [21, 22], and are widely used in cancer signature reversion drug repurposing applications [9, 10, 11, 12]. For the third approach, we developed disease‐associated gene signatures built on transfer learning latent variables. In this case, the transfer learning approach transfers biologically meaningful linear combinations of gene expression patterns (known as latent variables) from large databases to the tumor and control gene expression profiles [23]. To evaluate these methods in a cancer drug repurposing context, we focused on cancers with gene expression profiles available from The Cancer Genome Atlas (TCGA) that also had the lowest survival. These four cancers [pancreatic adenocarcinoma (PAAD, 5‐year survival < 8%) [24], liver hepatocellular carcinoma (LIHC, 5‐year survival < 18%) [25], glioblastoma (GBM, 5‐year survival < 4%) [26], and lung adenocarcinoma (LUAD, 5‐year survival = 5% for metastatic cases)] require novel drug candidates desperately [27, 28].

We demonstrated that these three approaches for selecting a disease‐associated gene signature for downstream signature reversion each captured enough unique biology to prioritize mostly different drug repurposing candidates. By comparing the identified drug candidates to drugs that have already progressed to clinical trials and the PRISM drug screen for each cancer, we validated these approaches for generating hypotheses for future *in vitro* and *in vivo* experiments. As a proof of concept, we further demonstrated that two novel drug repurposing candidates, which we predicted would be beneficial for GBM treatment (nimodipine and pamidronate), inhibited GBM growth *in vitro*. In addition to providing prioritized drug repurposing candidates for PAAD, LIHC, and LUAD, we conclude that by incorporating multiple approaches for selecting a disease‐associated gene signature for downstream signature reversion, additional viable cancer drug repurposing candidates can be identified.

## Results

### Selection and evaluation of disease‐associated gene signatures for each low‐survival cancer

In order to identify drug repurposing candidates, we first applied and evaluated three approaches for selecting a disease‐associated gene signature for downstream signature reversion. For each cancer (Data S1), we identified disease‐associated gene signatures by comparing TCGA tumor to TCGA and GTEx non‐cancer tissue gene expression profiles with two differential gene expression methods (limma and DESeq2) and a transfer learning approach, MultiPLIER [23]. Briefly, the DESeq2 algorithm modeled the gene expression counts as a negative binomial distribution and generated generalized linear models to determine differentially expressed genes while limma's approach uses gene‐wise linear models [19, 20]. These differential gene expression approaches provided adjusted *P*‐values and log_2_ fold changes for gene expression between conditions that have been used to develop disease‐associated gene signatures [9, 10, 11, 12]. To develop a disease‐associated gene signature via a transfer learning approach, we first validated the transfer of information via the gene labels of the transfer learning approach (see ‘Differential latent variable analysis’ in Methods) and then identified the latent variables that are different between cancer and non‐tumor tissue via latent variable‐wise linear models with limma [19, 23]. From these latent variables, we determined the genes that contributed the most to the latent variables (i.e., have the highest weights in the latent variable linear gene expression equation) to develop the disease‐associated gene signature.

**Fig. 1:**
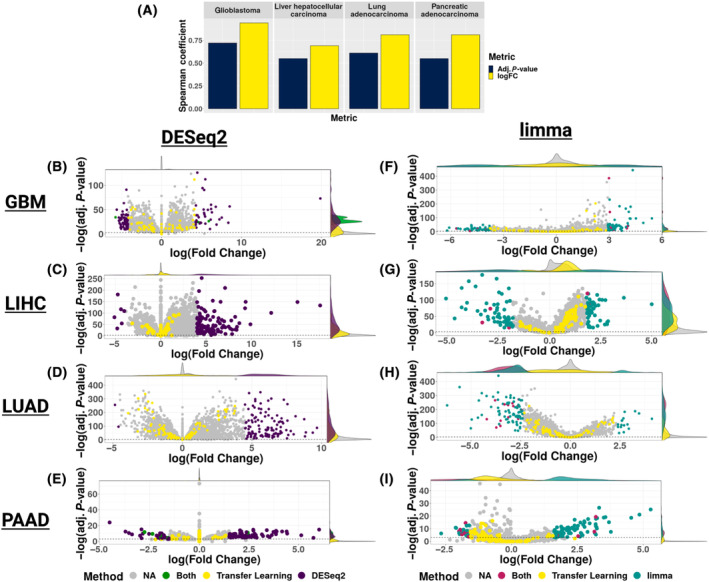
Comparison of gene inclusion in disease‐associated gene signatures for downstream signature reversion. (A) Bar plot of the Spearman correlation of adjusted *P*‐values and log fold changes between the disease‐associated differential gene expression signatures across each cancer. Volcano plots of DESeq2 calculated log fold change and adjusted *P*‐values to compare with transfer learning disease‐associated gene signature genes identified across each cancer (purple = DESeq2 disease‐associated signature genes, yellow = transfer learning disease‐associated genes, green = genes shared by both the DESeq2 and transfer learning disease‐associated gene signatures, gray = genes not in the DESeq2 or transfer learning disease‐associated gene signatures) for (B) GBM, (C) LIHC, (D) LUAD, and (E) PAAD. Volcano plots of limma calculated log fold change and adjusted *P*‐values to compare with transfer learning disease‐associated gene signature genes identified across each cancer (teal = limma disease‐associated signature genes, yellow = transfer learning disease‐associated genes, magenta = genes shared by both the limma and transfer learning disease‐associated gene signatures, gray = genes not in the limma or transfer learning disease‐associated gene signatures) for (F) GBM, (G) LIHC, (H) LUAD, and (I) PAAD.

We next compared the disease‐associated gene signature sets identified by DESeq2, limma, and transfer learning within each cancer. First, we calculated the Spearman correlation between log_2_ fold change and the adjusted *P*‐value from DESeq2 and limma. We found that all of the correlations were significant (linear regression model *P*‐values < 2.2e‐16) (Fig. S2). While the log_2_ fold changes had a correlation coefficient between 0.69 and 0.94 for each cancer, the correlation coefficient for the adjusted *P*‐values was between 0.55 and 0.72 (Fig. 1A). This suggests that the differences between the genes included in the DESeq2 and limma disease‐associated signatures were not due to differences in log_2_ fold change. Instead, gene inclusion was driven by differences in the adjusted *P*‐value methods (Fig. 1A). Second, to compare the DESeq2 and limma disease‐associated gene signatures with the disease‐associated gene signature identified by transfer learning, we labeled the transfer learning genes in the volcano plots of the DESeq2 and limma differential expression analyses. The volcano plots demonstrated that the transfer learning genes did not have larger absolute log_2_ fold changes compared to genes identified as significantly differentially expressed by either DESeq2 or limma (Fig. 1B–I). This underscores that the genes with the highest weights in the differential expressed latent variables identified by transfer learning were not the top differentially expressed genes identified by high absolute log_2_ fold change. Also, several transfer learning genes had non‐significant adjusted *P*‐values in the DESeq2 analysis (i.e., ~ 31% in GBM, ~ 16% in LIHC, ~ 9% in LUAD, ~ 73% in PAAD) or in the limma analysis (i.e., ~ 45% in GBM, ~ 5% in LIHC, ~ 11% in LUAD, and ~ 61% in PAAD). In total, these results highlight that each approach for selecting a disease‐associated gene signature identified gene sets that were largely non‐overlapping. In fact, the overlap in the disease‐associated gene signatures was 0.8%, 0%, 0%, and 1% for GMB, LIHC, LUAD, and PAAD, respectively (Fig. S3).

Next, we used the STRING database to construct a protein–protein interaction (PPI) network to assess the degree (the number of immediate neighbors in a network), betweenness (quantification of the node importance in information flow), and eigenvector centrality (a measure of the node's degree along the degree of neighboring nodes) of the genes in each of the disease‐associated signatures [29]. Previous studies have shown that FDA‐approved or Phase 4 drug targets have higher network centrality than other genes in PPI networks because of higher degree, higher betweenness, higher bridge centrality (a metric that describes nodes that connect modular subregions of a network), lower average shortest path and lower topological coefficient (a measure for the extent to which a node shares neighbors with other nodes) network centrality metrics [30, 31, 32, 33]. Therefore, we wanted to compare the PPI network properties of the unique genes selected for each cancer's disease‐associated signature. We found that the unique genes identified in the transfer learning disease‐associated signature have a significantly higher degree, betweenness, and eigenvector centrality than the unique genes identified by either DESeq2 or limma in GBM, LUAD, and LIHC (Kruskal–Wallis and two‐tailed Wilcox tests followed by a Bonferroni *P*‐value adjustment, adjusted *P*‐values < 0.05) (Figs S4 and S5). However, in PAAD, limma's and transfer learning's unique disease‐associated genes were not significantly different for all three centrality metrics (Fig. S5D–F). While PPI network degree, betweenness, and eigenvector centrality metrics for the unique genes identified by DESeq2 were the lowest, followed by those from limma, and then transfer learning, these comparisons were not always statistically significant. Our results highlight that transfer learning may identify disease‐associated genes with a higher PPI degree, betweenness, and eigenvector centrality than DESeq2 or limma. We further investigated the centrality metrics (i.e., degree, betweenness, and eigenvector centrality) of the highest‐weighted genes in all of the latent variables used for transfer learning (*n* = 385). We found that the 10 highest weighted genes for each latent variable (i.e., the most important genes for defining that latent variable) had significantly higher degree, betweenness, and eigenvector centrality than genes that were not in the 10 highest weighted genes for any latent variable (Fig. S6). Therefore, by using the top‐weighted genes from the latent variables that we identified as significantly different between cancer and non‐disease control samples, the transfer learning approach might have been biased to select disease‐associated gene signatures with higher centrality genes (based on degree, betweenness, and eigenvector centrality from the disease‐associated gene signatures and the top 10 highest weighted genes from all the 385 latent variables) than in the disease‐associated signatures constructed from the DESeq2 or limma differential expression analyses.

**Fig. 2:**
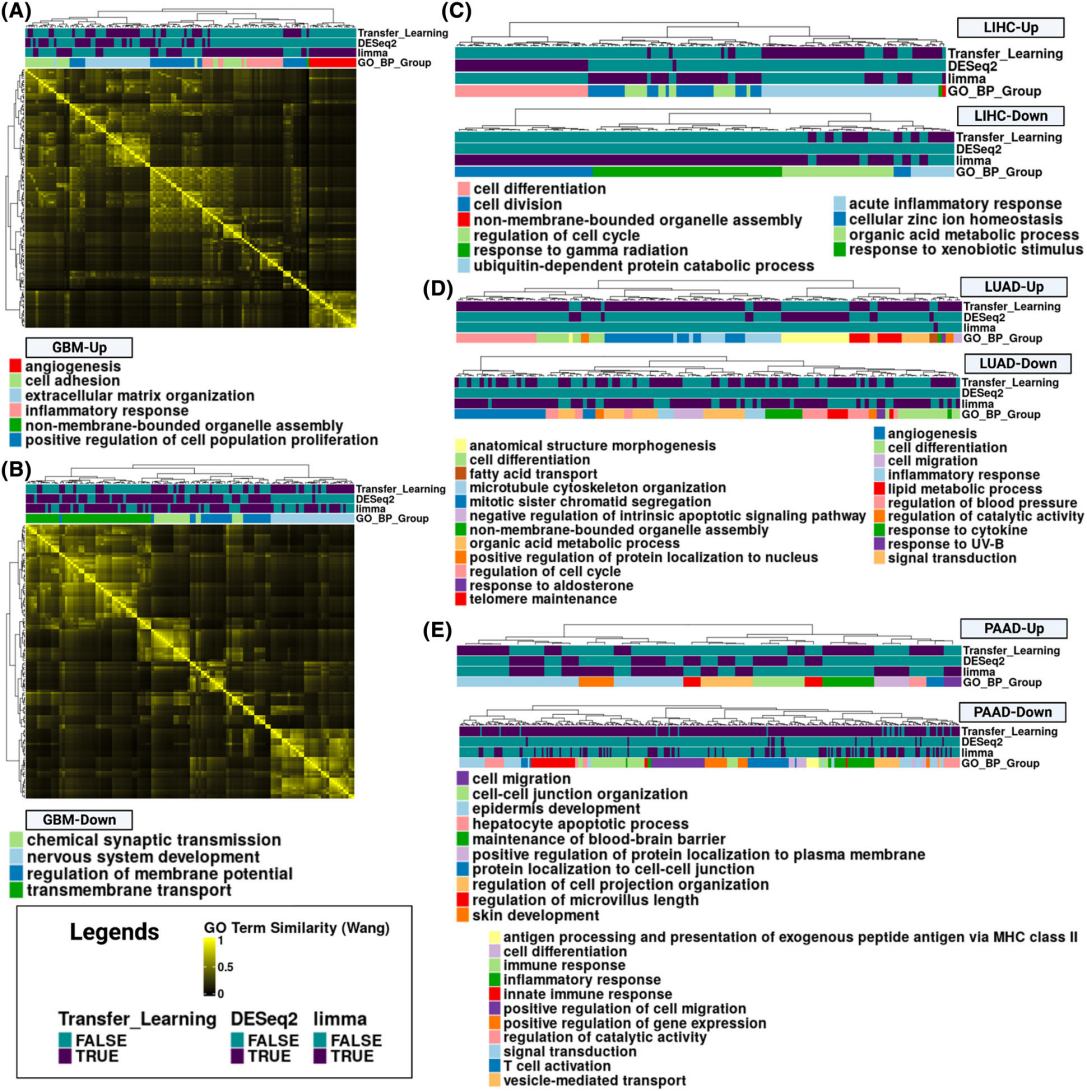
Functional enrichment analysis of disease‐associated gene signatures used for signature reversion. (A) Heatmap of the Gene Ontology (GO) term semantic similarity (Wang method) of the up‐regulated enriched GO Biological Process terms from the GBM disease‐associated gene signatures. Each term is associated with a disease‐associated gene signature if the row for that method is purple. In addition, the Gene Ontology Biological Process terms were grouped together based on common parent terms, and the different groups are indicated in the GO_BP_Group. (B) A heatmap of the GO term semantic similarity (Wang method) of down‐regulated enriched Gene Ontology Biological Process terms for GBM. (C–E) Dendrogram from the up and down‐regulated GO Biological Process terms for LIHC, LUAD, and PAAD cancer, respectively. Up‐regulated terms are plotted on top and down‐regulated terms on the bottom. The legend for the up‐regulated GO term groups is listed at the top and left. The down‐regulated terms are listed at the bottom and the right.

We next asked if the three approaches identified distinct genes from common pathways. Therefore, we applied functional enrichment analysis of gene sets from Gene Ontology (GO) [34], KEGG [35], Reactome [36], WikiPathways [37], TRANSFAC [38], miRTarBase [39], Human Protein Atlas [40], and The Comprehensive Resource of Mammalian Protein Complexes (CORUM) [41] to identify any pathways or gene sets enriched in each disease‐associated gene signature [42]. We observed that 10% or less of pathway terms were shared across all three disease‐associated gene signatures by cancer (Fig. S7). The largest was a ~ 9% overlap in the up‐ and down‐regulated pathways for the GBM disease‐associated gene signatures (Fig. S7A,B). To determine how similarly enriched biological process terms were between the three approaches within each cancer [43], we applied GO semantic similarity. We divided enriched GO terms into subgroups (GO_Group) based on semantic similarity and a common parent term in the GO graph to group similar GO terms together. Based on this hierarchical clustering of GO terms, we found similarities between the disease‐associated gene signatures within each cancer (Fig. 2). However, we also found enrichment of biological processes previously implicated in disease progression or therapeutic relevance that were not shared between all the disease‐associated gene signatures. For example, in the GBM up‐regulated enriched GO terms (Fig. 2A), we found that all three approaches had terms associated with cell adhesion, extracellular matrix organization, and positive regulation of cell population proliferation, pathways which are all known to be perturbed in cancer. However, terms were also associated only with a specific approach's gene set. For example, pro‐inflammatory response in microglia has previously been associated with GBM progression by single‐cell studies [44]. Several pro‐inflammatory gene sets were enriched in the limma disease‐associated gene signature, as were terms related to angiogenesis (i.e., blood vessel development, circulatory system development, vasculature development). Angiogenesis is a common molecular feature of GBM and the therapeutic target of bevacizumab, which is FDA‐approved for recurrent GBMs [45]. However, the down‐regulated GO terms enriched for each of the three disease‐associated gene sets were more similar (Fig. 2B). All three analyses also identified pathways associated with dysfunctional brain tissue (e.g., chemical synapse transmission, regulation of membrane potential, and transmembrane transport) and nervous system development, further highlighting signal de‐differentiation that has been associated with therapy resistance (Fig. 2B) [46].

While we identified similar GO terms represented in the disease‐associated gene signatures across cancers based on the GO similarity clustering, there were also distinct GO term subgroups identified by only one disease‐associated gene signature. We were interested in investigating the GO term groups that differed between the disease‐associated gene signatures, as these may drive the differences in the drug candidate sets. Many of the distinct GO term groups are associated with disease progression or response to therapy [47, 48, 49, 50, 51, 52, 53]. For example, the LIHC disease‐associated gene signature from the DESeq2 approach contained up‐regulated GO terms unique for the cell differentiation subgroup compared to the other two signatures (Fig. 2C, Fig. S8A). For LIHC down‐regulated GO terms, we found that the disease‐associated genes from the limma methodology were enriched for terms related to the response to xenobiotic stimulus and cellular zinc ion homeostasis (Fig. 2C, Fig. S8B). In LUAD (Fig. 2D, Fig. S9), we found the following distinct GO term subgroups from the respective disease‐associated gene signatures: up‐regulation of anatomical structure morphogenesis (DESeq2), down‐regulation of regulation of response to UV‐B (limma), and down‐regulation of lipid metabolic processes, as well as up‐regulation of five GO term subgroups (Transfer Learning). For PAAD, up‐regulated enriched GO terms included: regulation of the microvillus length (DESeq2), positive regulation of the protein localization to the plasma membrane, cell migration, & skin development (limma), and maintenance of the blood–brain barrier, hepatocyte apoptotic process, & protein localization to cell–cell junction (Transfer Learning) (Fig. 2E, Fig. S10A). Interestingly, the only two distinct GO term subgroups for down‐regulated PAAD disease‐associated gene signatures were enriched in the transfer learning signature: antigen processing and presentation of exogenous peptide antigen via MHC class II and cell differentiation (Fig. 2E, Fig. S10B). Overall, based on the similarity clustering of the enriched GO terms, these results highlight that the disease‐associated signatures were enriched for distinct GO terms. Thus, each approach for defining disease‐associated gene signatures identified different aspects of cancer biology to target via signature reversion.

### Identifying prioritized drug repurposing candidates for each cancer

**Fig. 3:**
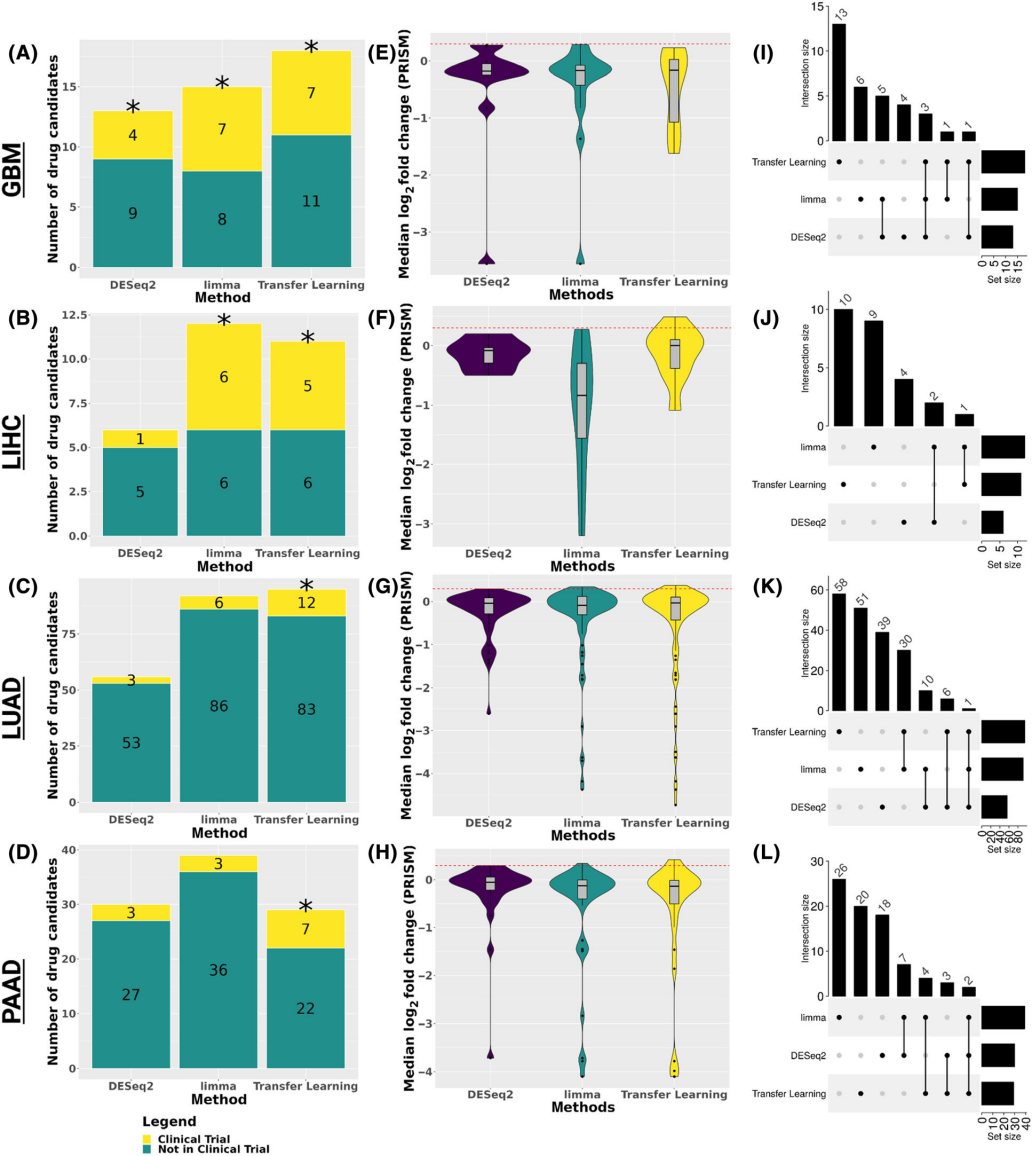
Performance of drug candidates predicated by signature reversion for each disease‐associated gene signature. The number of predicted drugs annotated by whether it has been in a clinical trial for (A) GBM, (B) LIHC, (C) LUAD, or (D) PAAD, respectively. The asterisks indicate enrichment of drugs in previous clinical trials compared to random drug selection (One‐tailed Wilcoxon rank‐sum test *P*‐value < 0.05). Violin plot of the median log_2_ fold change of cancer‐specific cell lines for candidates identified by the disease‐associated gene signatures where the red horizontal line indicates the 0.3 log fold change threshold indicating cell line sensitivity to the drug candidates in the PRISM primary screen for (E) GBM, (F) LIHC, (G) LUAD, and (H) PAAD. (I–L) Upset plots of the overlapping drug candidates between disease‐associated signatures from each method with a negative normalized connectivity score and a tau value < −80 for GBM, LIHC, LUAD, and PAAD, respectively.

To perform signature reversion, we applied the LINCS methodology in the SignatureSearch R package to identify drug repurposing candidates for each of the four cancers using each of the three disease‐associated signatures [13, 54]. We considered a drug as a potential candidate if it had a negative normalized connectivity score (i.e., the perturbation signature was inverse to the disease‐associated gene signature), a false discovery rate (FDR) < 0.05, and a Tau value (describes the overlap between the signatures) < −80. With the limma disease‐associated gene signature, we identified 15 FDA‐approved candidates for GBM, 12 candidates for LIHC, 92 candidates for LUAD, and 39 candidates for PAAD. With the DESeq2 disease‐associated gene signature, we identified 13 FDA‐approved candidates for GBM, 6 candidates for LIHC, 56 candidates for LUAD, and 30 candidates for PAAD. With the transfer learning disease‐associated gene signature, we prioritized 18 FDA‐approved candidates for GBM, 11 candidates for LIHC, 95 candidates for LUAD, and 29 candidates for PAAD (Fig. 3, Figs S11–S14).

We next assessed if the drug repurposing candidates identified for each disease‐associated signature and cancer have been in clinical trials or assessed by PRISM (a pooled drug screen of 930 cancer cell lines treated with 21 000 drugs to identify which inhibit cancer growth) for that cancer (Fig. 3, Figs S16–S19). We performed permutation testing of a matched number of random sets of drugs for each as a comparison (Data S2). The limma disease‐associated signature drugs were enriched for drugs identified as sensitive by the PRISM drug screen for all four cancers but only enriched for drugs in clinical trials compared to the random sets for 2 of the 4 cancers (i.e., GBM and LIHC). Transfer learning disease‐associated signature drugs were enriched for drugs in clinical trials compared to the random sets for all four cancers but only enriched for drugs identified as sensitive by the PRISM drug screen in two of the four cancers (i.e., GBM and PAAD). Finally, DESeq2 disease‐associated signature drugs were only significant compared to random sets of drugs in clinical trials for GBM. However, in the case of the PRISM drug screen, DESeq2 disease‐associated signature drugs were enriched for sensitive drugs for all four cancers. Across the cancers, there were similar performances for the positive controls between the methods. Still, a lack of overlapping candidates (discussed further in the next section) suggests that each of the three disease‐associated gene signature approaches identified different signatures, resulting in different but plausible candidates.

### Comparison by cancer of prioritized drug targets and mechanisms of action

For each of the disease‐associated gene signatures, most of the drug repurposing candidates identified through signature reversion were unique to a particular analysis: 50%, 79%, 61%, and 65% in GBM, LIHC, LUAD, and PAAD, respectively. Between the sets of candidates per cancer, the maximum overlap between them was 3 in GBM (~ 9% of all the candidates, Fig. 3I–L). The mechanism of action (MOA) captured by each set also varied (Figs S15–S18). This might be due to broad MOA (e.g., tyrosine kinase inhibitors) or incomplete knowledge about drug MOA and targets [e.g., cabozantinib, which was known to inhibit MET proto‐oncogene (MET) and vascular endothelial growth factor receptor 2 (VEGFR‐2), but later found to also target RET proto‐oncogene (RET)] [55]. Interestingly, each analysis found a diverse group of drugs with several different MOAs. GBM and LUAD had more MOA overlap than LIHC and PAAD. In LUAD, we identified drug repurposing candidates from all three disease‐associated gene signature reversion results with the glucocorticoid receptor agonist MOA. This MOA has been shown to induce cell dormancy and stop the growth of cell lines [56]. In LIHC, we found mTOR inhibitors in both the limma and transfer learning disease‐associated gene signature reversion candidates. These inhibitors have been previously tested in the treatment of liver cancer and are thought to impact several liver cancer cellular phenotypes, such as inflammation, angiogenesis, and metabolism [57]. Lastly, in PAAD, the drug formoterol (a β adrenergic receptor antagonist identified by DESeq2 and transfer learning disease‐associated gene signatures) and phentolamine (an α‐adrenergic receptor antagonist identified by limma disease‐associated gene signature) share a common MOA, adrenergic receptor antagonist. Within the literature, α‐adrenergic receptor antagonists have been shown to affect apoptosis and proliferation [58], and β‐adrenergic receptor antagonists have suppressed cell invasion [59].

**Fig. 4:**
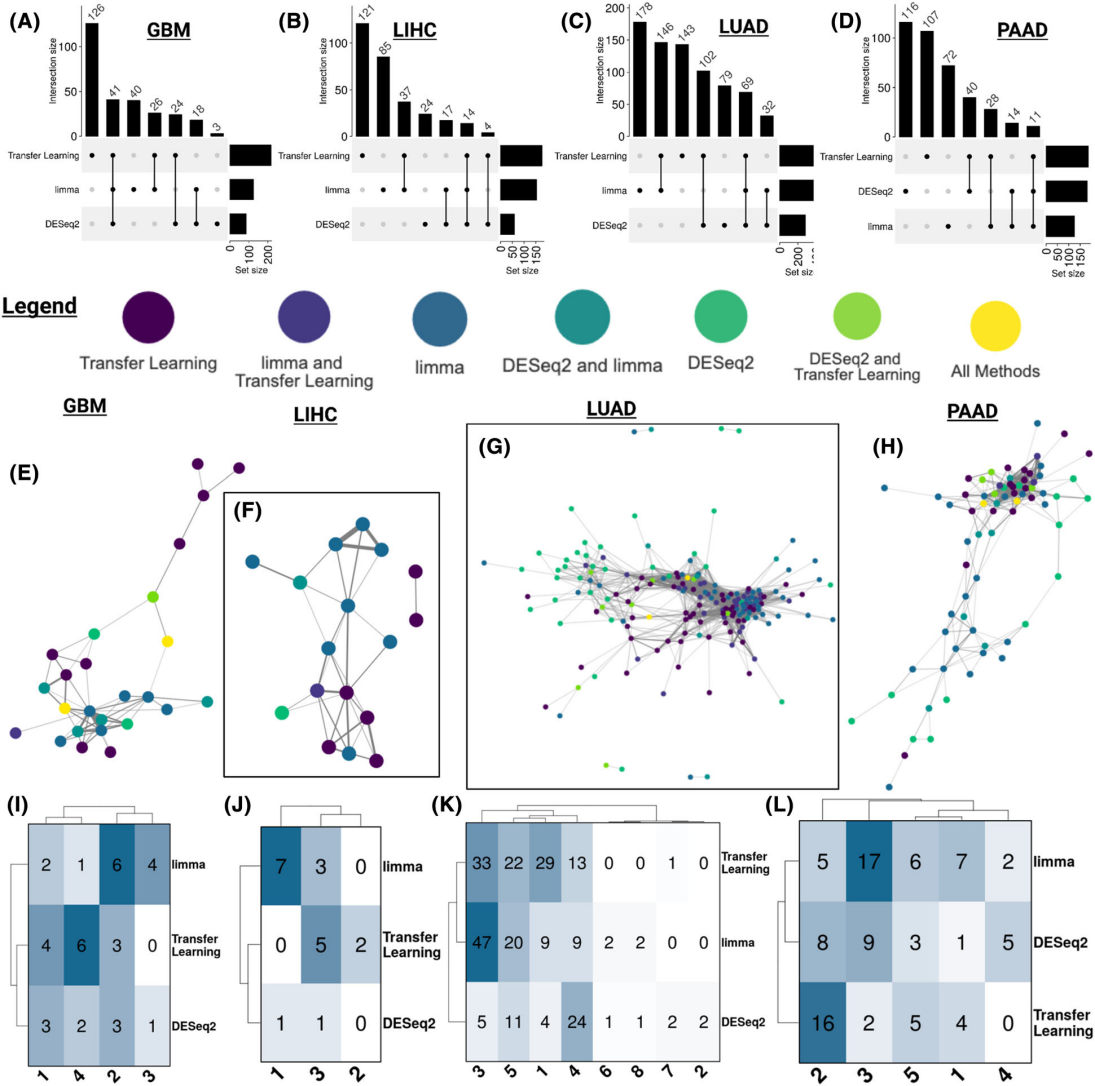
Drug–drug similarity network analysis for prioritized candidates. (A–D) Upset plots of the overlap between the known drug targets of the top identified drug candidates for GBM, LIHC, LUAD, and PAAD, respectively. Drug–drug similarity networks based on cosine similarity of LINCS perturbation profiles of candidates, where each node is a candidate colored by the disease‐associated gene signature used to identify that candidate and the top 90% of edges are displayed and weighted by cosine similarity for (E) GBM candidates (GI1 profiles), (F) LIHC cancer candidates (HEPG2 profiles), (G) LUAD candidates (A529 LINCS profiles), and (H) PAAD candidates (YPAC profiles). Heatmaps of the composition of the Leiden communities in the drug–drug similarity networks for (I) GBM candidates (GI1 profiles), (J) LIHC candidates (HEPG2 profiles), (K) LUAD candidates (A529 LINCS profiles), and (L) PAAD candidates (YPAC profiles).

We also investigated the overlap of the drug targets annotated from LINCS, DrugBank, CLUE, and STITCH databases for the identified candidates and found < 15% overlapped across all three analyses for a given cancer (Fig. 4A–D). GBM had the highest number of overlapping drug targets (~ 14.7% of drug targets), while LIHC had the lowest (~ 5% of drug targets). As expected, several of these shared drug targets for each cancer are cytochrome P450 (CYP450) enzymes, essential for drug metabolism. In addition, to determine if the identified candidate set differences between the three analyses within a cancer were due to cut‐offs, we conducted a Spearman's correlation between the normalized connectivity score (NCS) and FDR for each candidate in each analysis within the four cancers (Figs S19–S22). These correlations (NCS range: −0.027 to 0.59; FDR range: −0.12 to 0.38) suggest that the disease‐associated gene signature reversion analyses (i.e., DESeq2, limma, and Transfer Learning) result in different rankings of drug candidates (Fig. S23). Overall, signature reversion of disease‐associated gene signatures from three different approaches identified sets of drug candidates with different MOAs and drug targets.

Next, we wanted to determine if the candidates identified through signature reversion of different disease‐associated gene signatures perturb the same genes. We used the perturbation profiles (LINCS level 5 modified *z*‐scores) from the cancer‐specific cell lines (the same ones used in the signature reversion step of our study), to determine the gene expression changes induced by each drug repurposing candidate. We applied cosine similarity to these perturbation profiles between candidates and constructed a drug–drug perturbation similarity network for each cancer. These drug–drug networks allowed us to compare and evaluate the different perturbation profiles (i.e., the gene expression changes before and after treatment) between the various candidates. In our analysis, we found drug repurposing candidates for each cancer generally clustered by the disease‐associated gene signature approach used to identify them (Fig. 4E–H). This indicates that repurposing candidates identified from the same disease‐associated gene signature reversion analysis tend to perturb more similar gene sets than candidates identified from other disease‐associated gene signature reversion analyses. Thus, they are not perturbing the same genes across the three analyses. To further support this observation, we determined Leiden communities for each cancer drug–drug network and compared the composition of these communities (Fig. 4I–L). Drugs in the same Leiden community are more connected and thus more similar to each other compared to candidates in other communities. In GBM, which had the largest overlap of drug candidates across analyses, there were distinct communities with a higher number of candidates from one analysis, such as community 4 (transfer learning) and community 3 (limma) (Fig. 4I). These distinct communities with a large portion of candidates from one analysis were also seen in the LIHC, LUAD, and PAAD drug–drug networks (Fig. 4J–L). Across the different cancers, this highlights that drug candidate cell line gene expression perturbation profiles differ between the three disease‐associated gene signature reversion candidate sets.

Because previous studies show that drugs with similar structures tend to have similar efficacy and safety profiles [60], we also created drug–drug similarity networks based on drug structures using the Tanimoto coefficient between drug structures (Fig. S24). We found a similar trend to the drug–drug perturbation similarity network where repurposing candidates identified by signature reversion of the same disease‐associated gene signature clustered closer to each other than repurposing candidates identified by signature reversion of the other disease‐associated gene signatures. However, these structure similarity network clusters had more candidates from different disease‐associated gene signature candidate sets in the same communities than the drug–drug perturbation similarity networks (Fig. S24). This might be because several candidates did not have available drug structures (i.e., SMILES structures from customCMPdb R package LINCS data set). Overall, both the structure similarity and perturbation drug–drug networks further support that the candidates identified by each disease‐associated gene signature approach differ. Combined with our findings that drug candidates identified by reversion of each of the three disease‐associated gene signatures are similarly represented in clinical trials and drug responses in the PRISM cell lines, these disease‐associated signature methods identified disparate but potentially similarly effective drug repurposing candidates.

### Additional validation of prioritized GBM FDA‐approved drug repurposing candidates

**Fig. 5:**
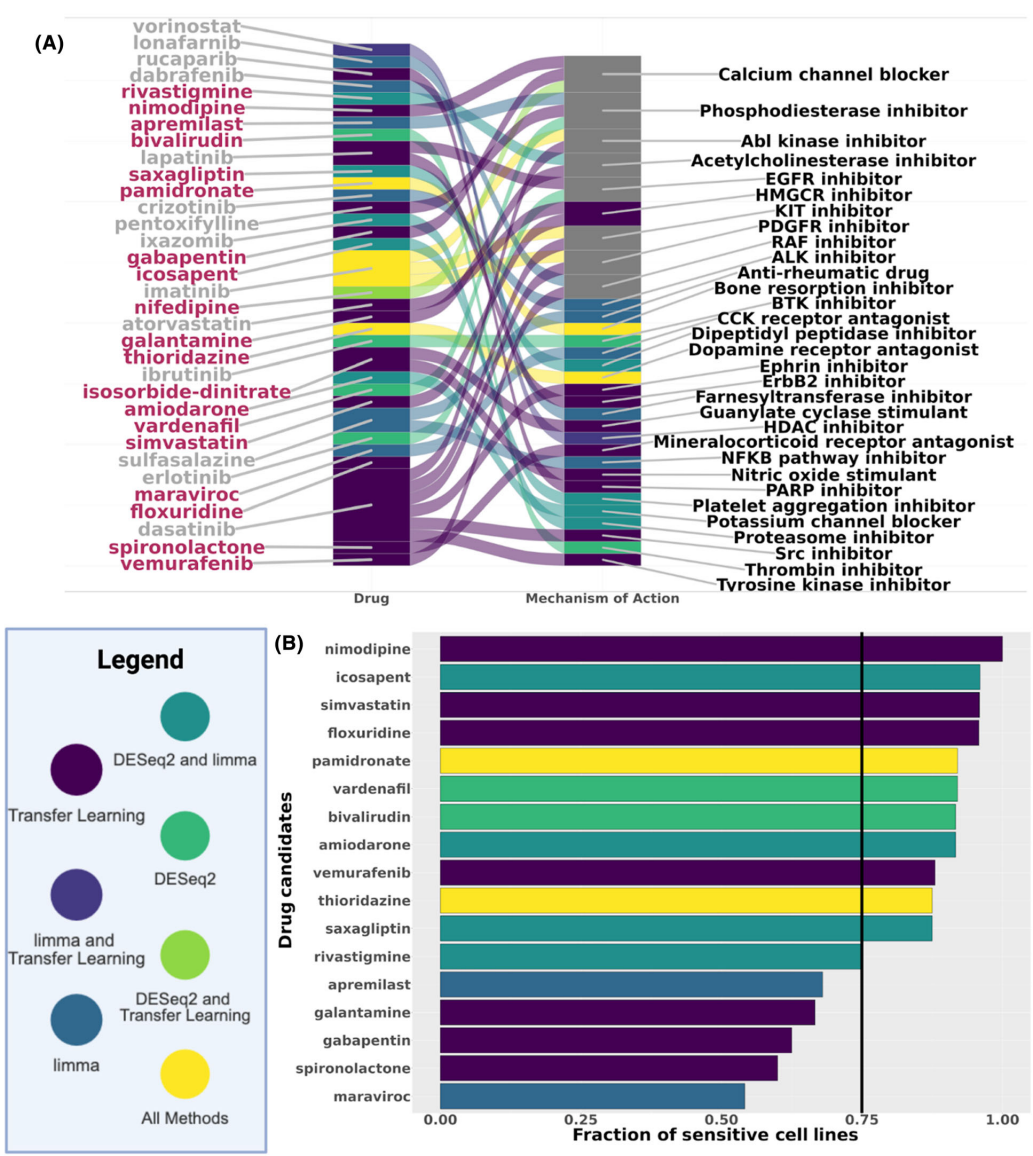
Prioritized GBM FDA‐approved drug repurposing candidates perturb multiple MOAs and impact GBM cell line growth. (A) Alluvial plot of the mechanism of action for the drug candidates from DESeq2, limma, and transfer learning disease‐associated gene signature reversion analyses with drugs ordered by the median rank across analyses and MOAs ordered by the number of candidates with that MOA and then by alphabetical order (gray = candidates that have been in a clinical trial for GBM, maroon = candidates have not been in a clinical trial for GBM). (B) The fraction of sensitive cell lines based on the PRISM primary screen for the top drug repurposing candidates that were not in a previous clinical trial for GBM. Black line at 0.75 indicates the fraction of GBM cell lines sensitive to the standard treatment, TMZ. For both plots, drug candidates are colored by the disease‐associated gene signature used to identify them as candidates.

**Fig. 6:**
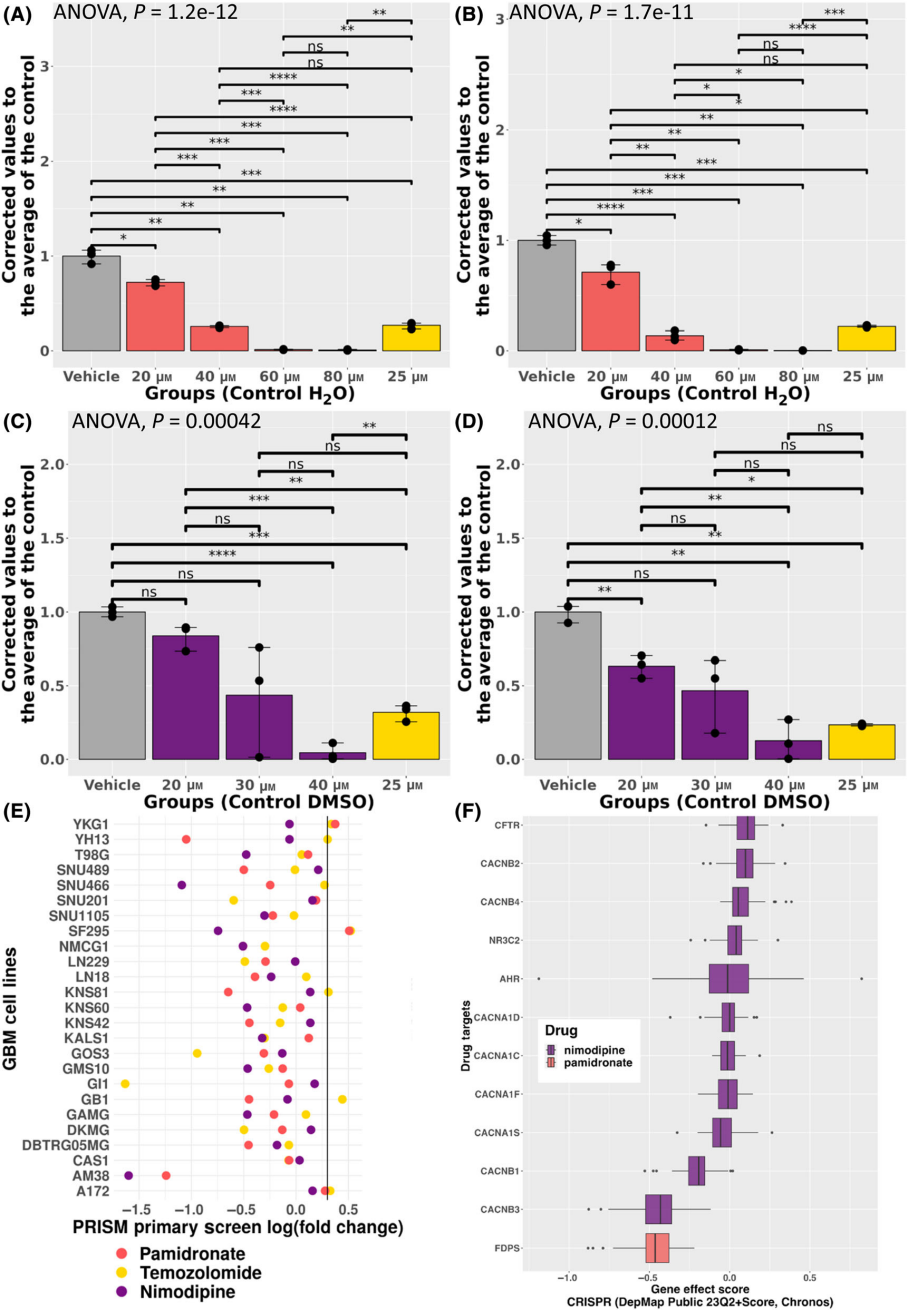
FDA‐approved pamidronate and nimodipine inhibit GBM growth *in vitro*. CellTiter‐Glo growth assay results for (A, B) pamidronate or nimodipine (C, D) in the GBM cell line U251 (A, C) or in cells derived from the GBM PDX JX39 (B, D). The yellow bar is the results from temozolomide‐treated cells. *P < 0.05, **P < 0.01, ***P < 0.001, ****P < 0.0001 ANOVA with Bonferroni corrected t‐tests for pairwise comparisons with standard error bars (representative experiment from three biological replicates each with minimum *n* = 3 technical replicates). (E) Dot plot of PRISM primary screen results by GBM cell lines. (F) Boxplot of the gene effect scores of the drug targets for pamidronate and nimodipine.

We further prioritized validating repurposing candidates for GBM because it is the rarest of the selected cancers studied here and has only one standard of care chemotherapy, temozolomide (TMZ) [61, 62]. The urgent need for new treatments for GBM that may come from drug repurposing is shown by the fact that TMZ only increased median survival by 2 months, MGMT promoter unmethylated patients are much less sensitive to the drug, and rapid tumor recurrence often occurs [63, 64]. In addition, our prioritized drug candidates included more drugs in GBM clinical trials and more GBM cell lines sensitive to treatment in the PRISM drug screen than selecting random drugs. This cancer also had the most overlap between candidates (3 out of 32) across the three disease‐associated gene signature reversion analyses (Fig. 5A). We first filtered for FDA‐approved drug repurposing candidates that have not been in a clinical trial for GBM and have more PRISM cell lines sensitive to treatment with our candidate than TMZ (Fig. 5B). This resulted in 11 FDA‐approved candidates from the three disease‐associated gene signature reversion results (two from DESeq2 and four from transfer learning, including three shared between DESeq2 and limma and two shared between all three). From those, simvastatin, floxuridine, vardenafil, amiodarone, and thioridazine had already been investigated for the treatment of GBM with *in vitro* and/or *in vivo* studies [65, 66, 67, 68, 69, 70, 71]. We did not further consider bivalirudin because there was evidence in DrugBank that this drug was unlikely to pass the blood–brain barrier. Vemurafenib was also excluded from further testing because of a basket clinical trial for gliomas with BRAFV600 mutation [72, 73]. Therefore, we had four remaining candidates: pamidronate, nimodipine, icosapent, and saxagliptin. We found compelling evidence for each of these with respect to their drug targets, perturbed pathways, and other *in vitro* drug testing [74, 75, 76, 77]. Thus, we conducted cell viability experiments to determine if each of the four candidates was capable of decreasing the growth of the U251 cell line, a GBM cell line not included in the PRISM drug screen, the JX39, a TMZ‐sensitive patient‐derived xenograft, and the D456, a TMZ‐resistant patient‐derived xenograft (Table 1). In a screen with concentrations ranging up to 100 μm, there was no effect of icosapant and saxagliptin in any of the cell lines tested (Fig. S25 and data not shown). In contrast, we observed significant effects of pamidronate and nimodipine (Fig. 6A–D, Fig. S26 and data not shown) and they were further evaluated.

**Table 1:** Key resources table.

Reagent or resource	Source	Identifier
Critical commercial assays		
CellTiter‐Glo® Luminescent Cell Viability Assay	Promega	G755A and G756A
RealTime‐Glo Annexin V Apoptosis and Necrosis Assay Promega	Promega	JA1011
Experimental models: cell lines and patient‐derived xenografts		
U251	C. Griguer, University of Iowa	
JX39	J. Sarkaria, Mayo Clinic	
D456	D. Bigner at Duke University	
Software and algorithms		
Cancer_Signature_Reversion scripts	This study	https://doi.org/10.5281/zenodo.7661401
PLIER	Mao *et al*. [107]	https://github.com/wgmao/PLIER
MultiPLIER	Taroni *et al*. [23]	https://github.com/greenelab/multi‐plier
DESeq2	Love *et al*. [20]	https://bioconductor.org/packages/release/bioc/html/DESeq2.htm l
Limma	Ritchie *et al*. [19]	https://bioconductor.org/packages/release/bioc/html/limma.html
SignatureSearch	Duan *et al*. [13]	https://bioconductor.org/packages/release/bioc/html/signatureSearch.html
jenfisher7/rstudio_cancer_dr (Docker Image)	This study	https://hub.docker.com/r/jenfisher7/rstudio_cancer_dr https://doi.org/10.5281/zenodo.7662005
jenfisher7/rstudio_tf_dr_v3 (Docker Image)	This study	https://hub.docker.com/r/jenfisher7/rstudio_tf_dr_v3 https://doi.org/10.5281/zenodo.7662126
SR_TAU_CELL_environment.yml (Conda environment)	This study	https://doi.org/10.5281/zenodo.7661401

For both FDA‐approved pamidronate and nimodipine, we conducted three independent cell growth drug screens of GBM cells propagated under brain tumor‐initiating cell conditions (in the absence of serum). Cells were from a standard cell line (U251) as well as isolated from two patient‐derived xenografts (JX39, D456) for which the brain tumor‐initiating cells have different sensitivities to TMZ (Fig. 6A–D, Fig. S27). We determined that a single treatment of 40 μm pamidronate or nimodipine was sufficient to significantly inhibit the growth of all of the GBM cells tested after 5–7 days (Fig. 6A–D and data not shown) and lower concentrations of the drug were effective in some cells with repeated treatment (Fig. S26). Furthermore, we determined that apoptosis was a mechanism of cell death for pamidronate but not nimodipine (Fig. S27).

Pamidronate, one of the top candidates for all the methods (ranked via the NCS as 2nd, 5th, and 10th from the DESeq2, transfer learning, and limma disease‐associated gene signatures, respectively), is an FDA‐approved bone resorption inhibitor used for the treatment of moderate to severe hypercalcemia of malignancy, Paget's disease, osteolytic metastases, and osteoporosis (Fig. 5A) [73, 78]. Pamidronate induces apoptosis of hematopoietic tumor cells by inhibiting farnesyl diphosphate and geranylgeranyl diphosphate [73]. Many of the most common adverse events of pamidronate were associated with the underlying disease state of the patients [79]. The common adverse events include hypertension and bone pain in the clinical studies for hypercalcemia of malignancy and Paget's disease [79]. There are box warnings related to deterioration in renal function and pregnancy for pamidronate [80].

In this study, we compared the treatment response of GBM cell lines treated with pamidronate to the standard of care, TMZ, in the PRISM cell line data set. While this dataset only contains cell line growth results and TMZ therapy is usually combined with radiotherapy in the clinic, we found that 12 of the 24 GBM cell lines treated with both drugs had a lower log_2_ fold change in cell growth with the pamidronate treatment, and across all GBM cell lines (*n* = 25) only two were identified as not sensitive to pamidronate treatment (log_2_ fold change > 0.3) (Fig. 6E). Further functional enrichment analysis of the GI1 GBM cell line gene expression perturbation profile (i.e., the up and down‐regulated genes from the LINCS database due to treatment) used in our signature reversion analysis identified several pathways associated with GBM. This included pathways associated with WNT signaling, development, and proliferation in the up‐regulated gene set, while acute inflammatory response and metabolism to fat‐soluble and retinoids were enriched in the down‐regulated gene set (Data S3).

We further investigated the known drug target of pamidronate, *FDPS*, by assessing the Chronos gene effect scores (i.e., infers if a gene is essential for cancer cell line survival) from the Project Achilles CRISPR/Cas9 genome‐scaled pooled loss‐of‐function (LOF) screens [81]. When we looked at the GBM cell lines, we found that *FDPS* had a mean gene effect score of −0.4683033, indicating that *FDPS* is a potentially essential gene in these GBM cell lines (Fig. 6F). In addition, while not identified as significantly differentially expressed by DESeq2 or limma, we observed increased gene expression of pamidronate's known drug target, *FDPS*, in primary tumor tissue (Fig. S28A). This suggests that pamidronate might particularly target tumor tissue itself. *FDPS* codes for an enzyme that has been implicated in the stem‐cell‐like characteristics of GBM that have been previously associated with GBM treatment resistance [82]. Pamidronate has also been previously shown to enhance paclitaxel‐induced apoptotic death in the U87MG GBM cell line [74]. To our knowledge, these PDX drug screen results are novel findings indicating GBM PDX‐derived cells are sensitive to pamidronate. PDX systems have been shown to better reflect patient molecular profiles and phenotypes, like drug response, than cell lines due to genetic drift experienced during passaging and long‐term growth in *in vitro* environments [83, 84]. Overall, these results from our drug screen supported pamidronate as a GBM drug repurposing candidate.

The other candidate, nimodipine, was the highest‐ranking drug repurposing candidate that was not in a previous GBM clinical trial from the transfer learning methodology for GBM (Fig. 5A). This FDA‐approved calcium channel blocker is used as an adjunct to improve neurologic outcomes following subarachnoid hemorrhage from a ruptured intracranial berry aneurysm [73]. Additionally, the most common adverse event for nimodipine (4.4% of 823 patients with subarachnoid hemorrhage in the clinical trials) was decreased blood pressure [85]. In previous studies, this drug was shown to be more effective in causing cancer cell death in combination with other anticancer drugs [75, 86]. In the PRISM drug screen, 16 of the 24 GBM cell lines treated with both nimodipine and TMZ were more sensitive to nimodipine (Fig. 6E). Additionally, this drug screen found all the GBM cell lines tested sensitive to nimodipine, while only 75% of the cell lines were sensitive to TMZ. In our *in vitro* experiments with cells derived from the D456 PDX, these GBM cells were sensitive to nimodipine but not TMZ (Fig. S26B). Furthermore, our DESeq2 differential gene expression analysis confirms that all 11 of the drug targets of nimodipine are differentially expressed at the gene level between GBM and control brain tissue samples (Fig. S28B). Also, within the Project Achilles CRISPR/Cas9 dataset, the drug target *CACNB3* had the lowest average gene effect score across the drug targets for nimodipine (−0.4559023), indicating that *CACNB3* is potentially essential in these GBM cell lines (Fig. 6F). The functional enrichment analysis of the GI1 perturbation profile of this drug further suggests that it perturbs pathways associated with metabolism, cellular processes, and the cytoskeleton (Data S3). Our study highlights novel FDA‐approved drug repurposing candidates for further investigation as potential treatments of GBM.

## Discussion

While previous cancer studies have applied signature reversion to identify drug repurposing candidates [11], we sought to investigate how different approaches to developing the disease‐associated gene signature for signature reversion impact downstream drug candidate selection. In four low‐survival cancers, we demonstrate that signature reversion identified drug repurposing candidates enriched for drugs in clinical trials or performed well in PRISM's cancer cell line drug screen [87, 88]. The candidates identified from the two differential expression (DESeq2 and limma) and transfer learning (MultiPLIER) disease‐associated signature approaches were not commonly shared, and candidates prioritized from the same disease‐associated gene signature were more similar to each other than candidates identified with other disease‐associated gene signature approaches. This observation indicates that these disease‐associated gene signature approaches identify unique biology that researchers can further leverage to prioritize drug repurposing candidates. Our deeper investigation of the disease‐associated gene signatures underscored how different the disease‐associated gene signature methods are by determining the gene composition and pathway enrichment of the disease‐associated gene signatures. Overall, the main finding of our study is that researchers should apply these different disease‐associated gene signature approaches in tandem to increase the number of prioritized drug repurposing candidates for downstream *in vitro* and *in vivo* testing.

In addition to being among the lowest survival cancers, some of the selected cancers also have unique disease features or data availability that we leveraged in our study. As previously mentioned, GBM is the rarest of the cancers in this study, which limited data availability, while the more common LUAD had the most drug candidates tested in a cancer‐specific cell line in the LINCS database (e.g., *n* = 6742 for the A529 LUAD cell line compared to 685 candidates for the GI1 GBM cell line). PAAD bulk tumor tissue RNA‐seq profiles are known to be more similar to non‐tissue profiles than most cancers because PAAD tumors are mostly composed of cancer‐associated fibroblasts and a small population of cancer cells [89]. Our principal component analysis highlights this: the pancreatic tumor samples cluster with non‐cancerous pancreatic tissue samples (Fig. S29). Despite the influence these factors may have on the disease‐associated gene signatures, signature reversion, and the subsequent prioritized repurposing candidates, we find that most of the prioritized candidates had been in clinical trials and/or performed well in PRISM drug screens. Therefore, we are optimistic that the approaches we present here are robust and that our prioritized candidates warrant further investigation by the research community.

We also demonstrated the potential use of transfer learning for identifying a disease‐associated gene signature for signature reversion. Transfer learning has previously been used to identify disease‐associated latent variables in several rare disease applications, including medulloblastoma, a rare brain tumor, and in other drug repurposing studies [90, 91]. Here, we compared its performance to other disease‐associated gene signature methods across multiple cancers. Overall, the transfer learning disease‐associated gene signature approach prioritized candidates in all four cancers that were either previously in clinical trials or demonstrated cancer cell line sensitivity in the PRISM database. Additionally, the top two predicted GBM drug repurposing candidates inhibited GBM cell growth in the *in vitro* GBM cell culture systems we tested. This approach may also be beneficial for identifying drug repurposing candidates for other heterogeneous complex diseases.

Many prior studies support the repurposing of the drug candidates we identified. Itraconazole, an antifungal medication we predicted from the LIHC transfer learning disease‐associated signature, has been shown to inhibit LIHC cell growth and promote apoptosis via several pathways (e.g., WNT, ROS, AKT/mTOR/S6K) and other death receptor pathways [92]. In another example, from the limma disease‐associated gene signature for LUAD, we prioritized cladribine, a chemotherapy indicated for leukemia. There is prior evidence that the A529 cell line (the reference cell line we used for our signature reversion analysis) was sensitive to cladribine and that this drug enhanced apoptotic cell death due to overexpression of DNase γ [93]. While this might indicate that specifically, the A529 cell line might be sensitive to this candidate, all of the other PRISM LUAD cell lines were also sensitive to cladribine. Additionally, we predicted albendazole, an antihelminthic used to treat neurocysticercosis (an infection of the nervous system caused by pork tapeworms) and other worm infections, via the DESeq2 disease‐associated gene signature for the treatment of PAAD. This drug has been shown to induce apoptosis and reduce proliferation and migration in pancreatic cell lines and an *in vivo* nude mouse xenograft model [94]. As expected, many candidates we identified are currently approved for another type of cancer (e.g., cladribine is used for leukemia treatment but predicted here for application in lung cancer). However, we also found many repurposing candidates currently approved for conditions that are not neoplasms (e.g., nimodipine candidates for GBM). The PRISM study also observed the identification of non‐neoplastic drugs for cancer applications [87]. In addition, some drugs have drug targets for different organisms, such as fungi and tapeworms (i.e., itraconazole and albendazole). This observation suggests that these drugs have off‐target or cancer context‐specific drug mechanisms that are not entirely understood, a potentially interesting research direction.

In addition to the *in silico* controls (i.e., the clinical trial and PRISM drug screen analysis), we conducted a preliminary drug screen of the top GBM drug repurposing candidates pamidronate, nimodipine, icosapent, and saxagliptin. Our *in vitro* drug screen demonstrated pamidronate and nimodipine inhibited GBM cell growth in U251, JX39, and D456 GBM cell culture systems under brain tumor‐initiating cell maintenance conditions. We also investigated the gene effect score of CRISPR screen and differential expression of drug targets and the GI1 cell line up‐ and down‐regulated genes in the LINCS profiles for pathways associated with GBM severity and progression (e.g., WNT pathway, proliferation, and metabolism) [95, 96]. While there needs to be more investigation of pamidronate and nimodipine to determine if they are ideal drug candidates for GBM, these results were a proof of concept that the GBM drug repurposing candidates predicted from the three disease‐associated gene signature reversion approaches were identifying potential novel candidates for GBM.

Pamidronate and nimodipine inhibited the growth of D456 cells, which were resistant to TMZ‐resistant. GBMs have intratumoral heterogeneity with both TMZ‐sensitive and TMZ‐resistant cancer cell populations. This intratumoral heterogeneity has been identified as a reason for TMZ treatment failure for many patients [97, 98, 99]. The ability of both pamidronate and nimodipine to inhibit growth in TMZ‐sensitive and TMZ‐resistant cancer cell populations is a potential advantage of these drug repurposing candidates that needs to be further explored.

While overall, we did not find that signature reversion of one disease‐associated gene signature approach outperforms the others, we did observe a specific challenge for developing a disease‐associated gene signature via the DESeq2 methodology. To generate the DESeq2 disease‐associated gene signatures, we used an absolute shrinkage log_2_ fold change cut‐off to identify a list of ~ 100 genes. For the LIHC and LUAD data sets, we observed that with DESeq2, there was more variance between 0 and the highest positive fold change compared to the variance between the lowest fold change and 0 for differential gene expression (Fig. 1C,D). This resulted in a disease‐associated gene signature that contained predominantly up‐regulated genes in cancer compared to non‐tumor tissue. This may have resulted in the observed lower performance of the DESeq2 disease‐associated gene signature drug repurposing candidates. Also, it is important to note that our analyses rely on gene expression differences which may or may not be present at the protein level.

The results from our cell culture experiments provided evidence of pamidronate and nimodipine inhibiting GBM cell growth in a cancer cell line and cells derived from two different PDXs. However, we did not observe any growth inhibitory properties of icosapent and saxagliptin, although those were predicted by the signature reversion drug repurposing approaches. There could be multiple reasons why these two candidates did not have the growth inhibitory properties predicted. Importantly, we conducted our *in vitro* experiments in the absence of serum under conditions that maintain brain tumor‐initiating cells/cancer stem cells to best recapitulate the disease. Brain tumor‐initiating cells represent an important tumor cell subset that must be targeted successfully in order to treat GBM, because these cells are known to be resistant to chemo and radiotherapy in comparison to non‐stem cancer cells isolated from the same tumor [100]. We tested the drug candidates against cells maintained as brain tumor‐initiating cells as we felt that successful targeting of these cells had the highest potential for clinical translation. However, this meant that our *in vitro* experimental conditions were not identical to that in the PRISM and LINCS drug screen pooled drug screens. Furthermore, it remains possible that these candidates might be successful in other model systems as all *in vitro* work with cancer cells has limitations and the disease is very heterogeneous (i.e., genetic drift and long‐term growth *in vitro* environments) [83, 84].

In addition, we focused on cancer‐specific cell line perturbation profiles for each cancer as multiple studies, including the LINCS project data release, found perturbation profiles of the same cell line with different drugs had more similarity than the same drug across different cell lines [11, 54]. This influence of the molecular context of cell lines on drug response is important because it affects the prediction of drug repurposing candidates and our orthogonal validation analyses. For example, we hypothesize that the drug structure drug–drug similarity networks did not cluster the candidates the same as the drug perturbation drug–drug similarity network analysis due to the effect of cell line context on the perturbation profiles. In addition, while the literature provides supporting evidence for our results, as evidenced in the above paragraph, additional context may be important. For example, EGFR has been a drug target for many therapies in clinical trials for GBM, including those we predicted by reversion of the transfer learning disease‐associated gene signature, erlotinib and lapatinib [101]. However, as many EGFR inhibitors have failed in GBM clinical trials, EGFR inhibition alone may not be a suitable target for GBM [101]. However, there is evidence that EGFR inhibitors might be therapeutic for some GBM patients and not others, underscoring the importance of disease heterogeneity and personalized medicine efforts to pair the right drug with the right patient at the right time. In addition, within GBM tumors, some tumor cells are sensitive to EGFR inhibitors, while some cells are not [101]. Thus, approaches for determining patient treatment by tumor subtypes or novel drug design to target multiple mechanisms across clonal populations are needed to combat tumor heterogeneity [101]. Ideally, future drug repurposing studies will be powered to segregate tumor profiles and LINCS‐like perturbation profiles to match these different subtypes or biomarkers for predicting therapies as we were limited here by the cell lines in the LINCS dataset, the dosing protocol (i.e., 24‐h and 10 μm dosing protocols), and single‐agent studies. These challenges highlight the most critical limitation of signature reversion: dependence on context‐specific perturbation signatures. Future development of perturbation signature resources and methods has the potential to improve this approach.

This study addressed the critical question in signature reversion drug repurposing of how different disease‐associated gene signature approaches impact downstream drug repurposing candidate selection for four low‐survival cancers. Here, we demonstrate the utility of transfer learning‐based disease‐associated gene signature approaches to signature reversion and prioritize candidates for four of the lowest survival cancers. By investigating the predicted drug candidates and the disease‐associated gene signatures for each approach, we found that each disease‐associated signature approach identified unique genes, pathways, and drug repurposing candidates. We validated our candidates using publicly available cancer cell line drug screen and clinical trial data and further validated top GBM drug repurposing candidates in cell line and xenograft systems. We also provide a valuable resource of prioritized candidates for future efficacy and safety studies. In conclusion, our results underscore how using each disease‐associated gene signature in tandem may identify additional prioritized drug repurposing candidates for further investigation.

## Methods

### Scripts, Dockers, and Conda environment

The scripts for this project are available on Zenodo at https://doi.org/10.5281/zenodo.7661401. In addition to the scripts here, the Docker images used for this analysis are publicly available on Docker Hub (jenfisher7/rstudio_tf_dr_v3 & jenfisher7/rstudio_cancer_dr) and Zenodo (https://doi.org/10.5281/zenodo.7662126 and https://doi.org/10.5281/zenodo.7662005). For the Tau calculations, a conda environment was used (SR_TAU_CELL_environment.yml).

All key resources used are summarized in Table 1. Detailed computer and package version information are included in Data S4.

### Data download

For this study, we downloaded RNA‐Seq count profiles from the Recount3 database using the Recount3 R package [102]. This database contains RNA‐Seq samples from tumor samples via The Cancer Genome Altas (TCGA) and control samples from TCGA and the Genotype‐Tissue Expression (GTEx) project (Access date for GBM/BRAIN, LIHC/LIVER, LUAD/LUNG, and PAAD/PANCREAS Recount3 project, respectively: December 2021, May 2022, May 2022, and May 2022). We used the rest of the human samples from the Sequence Read Archive (SRA) that were also processed and stored by Recount3 for the dimension reduction step for transfer learning (*n* = 316 443) (Access date: December 2021). The numbers of tumor and control non‐tumor samples for each cancer are listed in Data S1. For the GBM comparison, we filtered the TCGA samples to include only IDH‐wildtype due to the reclassification of brain tumors in 2021 [103]. In addition, because GBM can originate from almost any brain region, we also used all GTEx samples from any brain region [104]. A limitation of this is that the brain regions are known to vary in gene expression. Unfortunately, the GBM TCGA data does not include information on tumor location in their database; therefore, we could not use batch correction or regression to reduce the influence of the brain region for the GBM analyses.

The Library of Integrated Network‐based Cellular Signatures (LINCS) program created a database of gene expression profiles of cell lines before and after being exposed to perturbing agents such as small molecules (1131 cell lines and 41 847 drugs) [54]. We downloaded the Expanded CMAP LINCS Resource 2020's modified *z*‐score (level 5) for compound data (aka. small molecules) from https://clue.io/data/CMap2020#LINCS2020 (Access date: January 2022). The LINCS level 5 data has been uniformly processed to control for plate variation and had technical replicates combined. In addition, we accessed the metadata for the compounds, genes, cell lines, and signatures. For this study, we used signatures for the 10 μm at 24 h because it has more drug signatures than other concentrations and time points. In addition, for this study, we focused on the perturbation profiles of cancer cell lines derived from the particular cancer we are studying (i.e., GI1 for GBM, HEPG2 for LIHC, A529 for LUAD, and YAPC for PAAD).

We downloaded the Drugs@FDA database in March 2022 from https://www.fda.gov/drugs/drug‐approvals‐and‐databases/drugsfda‐data‐files (Accessed date: March 2022). From the marketing status and products tables, we generated a table including the method of drug delivery, dosage, active ingredients, and FDA approval (fda_product_info_df.csv). In addition, we used ClinicalTrials.gov to determine drugs in clinical trials for each of the different cancers (accessed date: GBM‐February 2022; LUAD‐June 2022; PAAD‐June 2022; LIHC‐June 2022). For each cancer, we searched by cancer name to collect all of the clinical trial information.

The Profiling Relative Inhibition Simultaneously in Mixtures (PRISM) project conducted a pooled drug screen that treated 930 cancer cell lines with 21 000 drugs to identify which inhibit cancer growth [87]. We downloaded the primary and secondary screen data from the Cancer Dependency Map (DepMap) portal (Accessed date: February 2022) [105]. We limited the analysis to a single cell line per cancer type since previous studies have shown that perturbation profiles of the same cell line across treatments were more similar than those derived from applying the same drug across cell lines [11, 54].

The Archilles project investigated essential genes in cancer cell lines via CRISPR‐Cas9 lost of function screens. We download the “CRISPR (DepMap Public 23Q2+Score, Chronos)” scores for the available drug targets of pamidronate (i.e., *FDPS*) and nimodipine (i.e., *AHR*, *CACNA1C*, *CACNA1D*, *CACNA1F*, *CACNA1S*, *CACNB1*, *CACNB2*, *CACNB3*, *CACNB4*, *CFTR*, and *NR3C2*) from the Cancer Dependency Map (DepMap) portal (Accessed date: June 2023) [105].

We downloaded a human protein–protein interaction network from the STRING database (accessed date: March 2022; version 11.5) [29]. This database contains known and predicted protein–protein interactions, including direct and indirect associations [29]. We divided the protein–protein interactions in the network by 1000 and filtered for interactions > 0.7, as a score > 0.7 indicates high confidence in that protein–protein interaction. We converted the protein Ensembl ID to the HGNC gene symbol for each protein using the provided STRING annotations. Additionally, we accessed the drug structure data files for drug candidates via customCMPdb's loadSDFwithName function for the LINCS dataset (Accessed date: July 2022) [106].

### Principal component analysis

We conducted principal component analysis (PCA) for each of the selected cancers and the GTEx control tissue. We then used the variance stabilizing transformation (VST) from DESeq2 to transform the raw count data from Recount3 data (download described in the previous section) [22] and used it for the PCA with the prcomp function in base R. The PC plots were visually inspected for clustering of samples based on sex, age, RIN score, and ischemic time. While the GTEx data did vary according to ischemic time and RIN score, as the TCGA dataset did not contain this information, they were not included as covariates in subsequent models.

### Transfer learning for disease‐associated gene signature identification

#### Dimension reduction and transfer learning

We normalized the gene expression profiles from the Recount3 database as transcript per million (TPM) normalized for the transfer learning approach. To decompose the TPM normalized gene expression profiles from Recount3 into latent variables, we applied the dimension reduction method pathway‐level information extractor (PLIER) [23, 107]. We removed the TCGA and GTEx samples from the downloaded Recount3 database (*n* = 316 443 samples remaining) to remove their influence on the resulting latent variables. This resulted in 385 latent variables. The PLIER algorithm dimension reduction approach features the L1 and L2 penalties in the loss function resulting in (a) latent variables reflective of annotated pathways (e.g., from Reactome) and (b) most genes having a zero weight (i.e., only a fraction of genes have a high and significant gene weight) [107]. We transferred these learned latent variables from Recount3 to the TPM normalized gene expression profiles in TCGA and GTEx via matrix multiplication using MultiPLIER [23].

#### Differential latent variable analysis

We wanted to validate the transfer of information via the gene labels of the transfer learning approach. We hypothesized that if the gene labels transfer information and the gene labels were switched, the transfer learning approach would not be able to identify significant latent variables. To test this hypothesis, we compared the GTEx brain frontal cortex and cerebellar hemisphere region samples from Recount3 by switching a fraction of gene labels starting at 10 up to 100% at 10% intervals. We performed each label switch step 50 times. We then calculated the average number of latent variables significant for each interval (adj. *P*‐value < 0.05; fold change of 0.05) (Fig. S30) and used a linear regression model to determine if the percent of gene labels switched influenced the number of latent variables (*R* ^2^ = 0.906 and *P*‐value = 1.381e‐05). This confirmed that when gene labels are randomly switched, the number of significant latent variables from the transfer learning approach decreases. This analysis indicates that information about the genes is indeed transferred in transfer learning.

To determine the disease‐associated gene signature for the signature reversion drug repurposing, we conducted a differential latent variable analysis via latent variable‐wise linear regression models and identified which latent variables are different between disease and control (limma) [19]. As we used data from TCGA and GTEx for the tumor and control samples, we included the database (TCGA/GTEx) as a covariate to reduce the influence of database‐specific technical noise. We applied a Bonferroni‐adjusted *P*‐value of < 0.05 as a cut‐off for whether a latent variable was significant. In addition, we used the absolute fold change of approximately 3 standard deviations away from the mean to identify the most different latent variables between tumor and non‐tumor samples for each cancer. For example, if 3 standard deviations from the mean resulted in an upper cut‐off of 0.27 and a lower cut‐off of −0.23, an absolute cut‐off of 0.25 was used. Then, the genes that contribute the most to the latent variables (i.e., had the highest weights in the latent variable linear gene expression equation) for each cancer were identified. The number of top genes for each cancer varied based on how many of those genes overlapped with genes in other latent variables and the genes assayed in LINCS. The target disease‐associated gene signature had approximately 100 genes. Table 2 notes the total genes for each cancer. Finally, we used the gene expression fold change from the DESeq2 analysis between disease and control tissues to determine if the genes were up or down‐regulated in the disease [20]. These up and down‐regulated genes were the disease‐associated gene signatures in the gene expression signature reversion method.

**Table 2:** Disease‐associated gene signatures across all the cancers. Table of the number of up‐regulated (Up) and down‐regulated (Down) genes included in the disease‐associated gene expression signature.

Method	GBM	PAAD	LUAD	LIHC
DESeq2	Up: 37 Down: 66	Up: 68 Down: 33	Up: 115 Down: 2	Up: 115 Down: 6
Limma	Up: 58 Down: 60	Up: 85 Down: 26	Up:16 Down: 92	Up: 49 Down: 57
Transfer Learning	Up: 52 Down: 54	Up: 25 Down: 95	Up: 47 Down: 69	Up: 62 Down: 26

### Differential gene expression analyses for disease‐associated gene signature identification

As mentioned in the Introduction, limma and DESeq2 consistently perform well in differential gene expression analysis, incorporate covariates, and are widely used in cancer signature reversion drug repurposing applications [9, 10, 11, 12, 21, 22]. It is also known that limma and DESeq2 can identify different genes [22, 108]. In comparison studies with limma and DESeq2 that used simulated datasets or samples with exogenous RNA (‘spike‐in’) of known quantities, there is no tool for differential expression that is best for all cases as reviewed by Conesa *et al*. [21]. One noted observation is that DESeq2 seems to perform poorly in a large sample and high variation data, and limma performs better under these conditions, which is common in human cancer RNA‐Seq data [22].

#### Limma

For each of the cancers, we normalized the raw counts from Recount3 for GTEx and TCGA as transcript per million normalized and log‐transformed (log(TPM + 1)). To analyze differentially expressed genes between disease and control, our limma design matrix included the experimental groups and the originating database (i.e., GTEx or TCGA) [19]. We then used the Bonferroni method for multiple hypothesis testing. Based on the limma differential expression results, we selected genes with an adjusted *P*‐value < 0.05 and the highest absolute log_2_ fold change. However, for the disease‐associated gene expression signature, we selected 90–120 genes with the highest and lowest log fold changes (based on the study by Yang *et al*. [11]) that overlapped with genes in the LINCS database. If the gene did not overlap, we removed it and used the next gene with the highest or lowest fold change in the signature. The number of genes in the disease‐associated gene signature for each method is listed in Table 2.

#### DESeq2

For each cancer, we used the raw counts from gene expression Recount3 for the GTEx and TCGA samples for the DESeq2 differential expression analysis, identifying differentially expressed genes between each cancer and non‐disease sample set. The DESeq2 design formula also included the experimental groups and the database with other DESeq2 parameters at default values [20]. Based on the DESeq2 results, we considered genes with an adjusted *P*‐value < 0.05 and highest absolute log_2_ fold change as significantly differentially expressed. Again, we used the top ~ 90–120 highest and lowest log fold changed genes for the disease‐associated gene expression signature.

### Signature reversion

For this study, we used the enrichment approach applying a bi‐directional weighted Kolmogorov–Smirnov statistic via the signatureSearch R package [13] because it has been shown to perform better at identifying similar drugs based on MOA and drug structure similarity than other enrichment approaches [13]. The LINCS signature reversion method signatureSearch identifies drug candidates with a gene expression perturbation profile in LINCS most inverse to the disease profile (in this case, the disease‐associated gene expression signatures) [13]. This approach ranks drug candidates by the normalized bi‐directional weighted Kolmogorov–Smirnov statistic (to determine how similar disease and drug profiles are to each other) normalized by dividing the statistic by the signed mean of all the statistics from the subset of signatures corresponding to the same cell line and drug in the LINCS reference. We then filtered drug candidates based on the FDR to correct for multiple hypothesis testing and on the Tau values that were determined based on the calculation conducted by the signatureSearch package [13]. Tau values describe the uniqueness of the overlap between the perturbation signature and disease‐associated gene signature. A Tau value of −100 means that the negative connectivity score (i.e., a more inverse signature) from the comparison between X perturbation signature and the disease‐associated gene signature was more negative than that same X perturbation signature to other perturbation signatures in the reference database (here, the LINCS 2020 database). For example, the negative normalized connectivity score (NCS) from the signature reversion analysis for perturbation X is less than NCS scores from X perturbation signature to other perturbation signatures in the LINCS database. A Tau value of 100 indicates that the positive connectivity score (i.e., a closer matching signature) from the comparison between the perturbation and disease‐associated gene signature was more positive than that same perturbation signature to other perturbation signatures in the LINCS 2020 database. Tau values close to 0 suggest that the overlap between disease‐associated gene signatures and perturbation signatures is not a unique overlap for the perturbation. Overall, the Tau value helps identify drugs uniquely inverse to the disease‐associated gene signature [54]. In addition, we focused on the signature reversion results for the specific cancer cell line of interest (i.e., GI1 for GBM, HEPG2 for LIHC, A529 for LUAD, and YAPC for PAAD).

### Drug target analyses

The signatureSearch signature reversion results include information about the candidate's drug targets. From this information, we plotted drug target gene expression (TPM) and the DESeq2 adjusted *P*‐values to determine if there was a significant difference in gene expression of those targets between tumor and non‐tumor control samples [20]. In addition, we applied the signatureSearch R package drug set enrichment analysis (dsea_hyperG hypergeometric test [13]) to the FDA‐approved drug candidates identified for each cancer with each disease‐associated gene expression signature and their drug targets. In addition, we filtered the Chronos gene effect scores for CRISPR‐Cas9 LOF screens in GBM cell lines (*n* = 44) and plotted them for the drug targets of the top two GBM drug repurposing candidates, pamidronate and nimodipine. A gene effect score of zero indicates that a gene is not essential, and a negative gene effect score indicates that a gene is possibly an essential gene. Most cancer‐essential genes have a gene effect score near negative one.

### Clinical trial evaluation

To evaluate if signature reversion of disease‐associated gene expression signatures from a particular differential expression approach was able to identify more drug candidates that were already in clinical trials for a specific cancer, we performed permutation testing. We randomly selected the same number of FDA‐approved drugs 1000 as were identified as significant for each case, and determined what fraction of randomly selected drugs were in clinical trials for that specific cancer. We performed a one‐tailed Wilcoxon rank sum test to determine if the fraction of FDA‐approved drugs selected for each case was higher than for the randomly selected drugs.

### Drug candidate performance in the PRISM drug screen

We used the PRISM primary and secondary pooled drug screens to further evaluate if the identified candidates could reduce cell growth of cancer cell lines derived from the same cancer as the drug identified for repurposing [87]. For the primary screen, PRISM has calculated the median of log fold change median fluorescence intensity between replicates of a cell line treated with a drug. They considered a cell line sensitive to a treatment if the median‐collapsed fold change was < 0.3. We also assessed if a given approach for identifying differentially expressed genes for the disease‐associated gene expression signatures for signature reversion results in a set of drug repurposing candidates with a larger fraction of candidates that cancer cell lines are sensitive to (for the specific cancer in question) than an equal size set of randomly selected drugs 10,000 times and then performed a one‐tailed Wilcoxon rank‐sum test.

### Comparing genes included in the disease‐associated gene signature from different approaches

To investigate how different the log fold change and adjusted *P*‐values from DESeq2 and limma were, we calculated Spearman correlations. DESeq2 uses the apeglm method for effect size shrinkage in their fold change calculations to reduce large estimates of log_2_ fold change caused by high variance or low expression of certain genes that are not representative of the true difference between disease and control [109]. Limma, on the other hand, assumes that the input data is log_2_‐transformed and then calculates the mean gene expression difference between disease and control by gene [19]. Because of these differences in algorithms and statistical models within each approach, the log_2_ fold changes and adjusted *P*‐values for each method are not the same. To compare transfer learning disease‐associated signature genes with DESeq2 and limma disease‐associated signature genes, we constructed volcano plots of the log‐transformed fold change and the adjusted *P*‐value for both DESeq2 and limma. On these plots, we labeled genes by the disease‐associated gene signature they are a member of.

### Functional enrichment of disease‐associated gene signature genes

We used the R package gprofiler2 [110] for functional enrichment analysis to determine pathways and other annotated gene sets enriched in the up‐regulated and down‐regulated gene sets used for signature reversion [42]. These gene sets used in functional enrichment analysis include all the genes associated with the gene set term (e.g., cell proliferation), and genes within a gene set can have multiple functions associated with these gene set terms (e.g., positive and negative regulation). For example, up‐regulated genes with an enrichment of cell proliferation genes do not mean cell proliferation is up‐regulated. The accurate interpretation is that more up‐regulated genes were associated with cell proliferation than by chance. This analysis cannot assess the activity of a pathway.

We used the Biological Process GO terms in a Gene Ontology semantic similarity analysis. In this analysis, we compared the enriched GO terms from all disease‐associated gene signature sets for a given cancer by semantic similarity based on the method proposed by Wang *et al*. [111]. This approach considers the location of GO terms in the GO graph and their relationship to parent and children terms via the GOSemSim R package [43, 111]. We plotted the results in a heatmap and clustered via the wald.D2 algorithm using ComplexHeatmap [112]. We used functions from rrvgo (getGoTerm, loadOrgdb, getGoSize, reduceSimMatrix) to create GO term subgroups from the enriched pathways based on the parent term in the GO graph and the Wang semantic similarity [113].

### Functional enrichment analysis of LINCS profiles and annotation of top candidates

We analyzed the perturbation profiles (level 5 LINCS data) with gprofiler2 functional enrichment analysis to identify which pathways or transcription factors were perturbed by the drug candidates [42]. For each drug, the gene cut‐off for the functional enrichment analysis was the LINCS's level 5 modified *z*‐score > 2 for up‐regulated gene set and < −2 for the down‐regulated gene set. These modified *z*‐scores were calculated by the LINCS consortium by aggregating across the replicates within the level 4 data plate‐control normalized z‐scores. We investigated identified drug candidate targets, mechanisms of action, and blood–brain permeability with DrugBank, PubTator, and PubMed [73, 114, 115].

### Cosine similarity between drug candidate LINCS perturbation profiles

For each cancer, we calculated the cosine similarity between perturbation profiles in the LINCS level 5 data for the specific cell type we used as the reference for signature reversion [116]. We clustered these values in a heatmap [112] by Euclidean distance and the ward.D2 algorithm and then labeled each by the disease‐associated gene signature reversion analyses used to identify the drug candidate.

We developed a drug–drug similarity network with the highest 10% of cosine similarity weights for each cancer with the igraph and visnetwork R packages [117, 118]. In these networks, each node is a candidate where the color indicates the disease‐associated gene signature reversion analysis that identified that drug candidate, the edges are weighted based on the cosine similarity, and edge thickness corresponds to the strength of similarity between drug candidates. We applied the Leiden algorithm from igraph to identify drug candidate communities [117].

### Comparing signature reversion results between disease‐associated gene signature reversion analyses

We wanted to compare the signature reversion results between each disease‐associated gene signature reversion analysis by cancer. Therefore, we conducted Spearman correlation and linear regression to determine if there is a correlation between the normalized connectivity scores and false discovery rates for the three analyses per cancer. For this analysis, we focused on the signature reversion results for the specific cancer cell line of interest (i.e., GI1 for GBM, HEPG2 for LIHC, A529 for LUAD, and YAPC for PAAD).

### Drug structure similarity analysis

For each cancer, we calculated the Tanimoto Coefficient between drug structures from customCMPdb via the ChemmineR package [106, 119]. We clustered the Tanimoto Coefficients in a heatmap by Euclidean distance and ward.D2 clustering and labeled drug candidates by which disease‐associated gene signature reversion analysis identified the drug candidate. We also constructed drug–drug similarity networks based on drug structure similarity (Tanimoto Coefficient) [117, 118]. In these networks, each node is a drug candidate colored by the disease‐associated gene signature reversion analysis that identified it, and the edges are weighted based on the Tanimoto Coefficient, where their thickness correlates with the Tanimoto Coefficient. Therefore, the thicker the edge, the stronger the similarity between candidates.

### Network centrality analysis

For this analysis, we considered a gene high in weight if it was one of the 10 most highly weighted genes contributing to a latent variable. We determined this threshold by sampling the top genes and evaluating the gene overlap between different latent variables. We determined that there was a progressively larger overlap between top genes of different latent variables as the gene set was increased (Fig. S7A). For each gene in the protein–protein interaction network from the STRING database, we calculated the degree, betweenness, and Eigenvalue of centrality [29, 117] and performed two‐tailed Wilcoxon rank‐sum tests for each network metric followed by a Bonferroni *P*‐value adjustment. We log_2_‐transformed the centrality metrics for plotting and visualization. We compared the distribution of the log_2_‐transformed metrics of the unique genes included in the disease‐associated gene signature by each of the three approaches (i.e., DESeq2, limma, transfer learning). We did not consider overlapping genes to maintain the assumption of independence for both the Kruskal–Wallis and pairwise Wilcox tests. Then we conducted a Kruskal–Wallis test to determine if there were differences between the three sets within a cancer. If the Kruskal–Wallis test was significant (α = 0.05), then we applied a pairwise Wilcoxon rank‐sum test with Benjamini Hochberg *P*‐value adjustment.

### Information about cell culture systems for *in vitro* cell viability assay

The following cell culture systems were gifted to the Hjelmeland lab: U251 (CVCL_0021, parental, TMZ sensitive) from C. Griguer at the University of Iowa, D456 (parental, TMZ resistant) from D. Bigner at Duke University, and the JX39 (parental, TMZ sensitive) from J. Sarkaria, Mayo Clinic. Within the last three years, the U251, D456, and JX39 were authenticated by short tandem repeat (STR) analysis, yielding more than an 80% match in profiled loci. For this study, all cell lines were identified as mycoplasma‐free using a Polymerase Chain Reaction (PCR) assay.

### Information about drug treatment for *in vitro* cell viability assay

For the cell viability assays, pamidronate was resuspended in water to 100 mm, which was then serially diluted in water to produce 1000× stocks for use in drug treatments. Water was used as the vehicle control for all experiments with pamidronate. Nimodipine, saxagliptin, and icosapent were resuspended in DMSO to 50 mm, which was then serially diluted in DMSO to produce 1000× stocks for use in drug treatments. DMSO was used as the vehicle control for all experiments with nimodipine, saxagliptin, and icosapent.

### GBM *in vitro* cell viability assay

To conduct the cell viability assay, we plated 1000 U251 GBM cells, 1000 D456 GBM cells isolated from patient‐derived xenograft, or 2000 JX39 GBM cells isolated from patient‐derived xenograft per 96 wells in DMEM/F12 in brain tumor‐initiating cell maintenance conditions, We started treating at 20, 10, 1, and 0.1 μm with drug treatments on Day 1, 3, and 5 and quantifying on Day 7 with CellTiter‐Glo, similar to our published reports [120, 121]. We changed the media with the treatments on Day 3 and 5.

### GBM *in vitro* apoptosis and necrosis assay

We conducted a RealTime‐Glo Annexin V Apoptosis and Necrosis Assay normalized to Cell Titer Glo. We measured the Annexin V mediated luminescence with 48 h pamidronate or nimodipine (50 μm) treatment of 10,000 cells isolated from JX39 and D456 GBM patient‐derived xenografts.

## Conflict of interest

The authors declare no conflict of interest.

## Author contributions

JLF contributed to conceptualization, methodology, software, formal analysis, investigation, data curation, writing—original draft, writing—review & editing, and visualization. EJW contributed to methodology, validation, and writing—review & editing. VHO contributed to conceptualization, methodology, and writing—review & editing. TCH contributed to writing—review & editing and project administration. SEG contributed to the investigation and formal analysis. VLF and ADC contributed to validation and writing—review & editing. ABH contributed to investigation, formal analysis, resources, and writing—review & editing. BNL contributed to conceptualization, methodology, resources, writing—review & editing, supervision, project administration, and funding acquisition.

## Supporting information


**Fig. S1.** Detailed overview of the signature reversion with three disease‐associated gene signatures.
**Fig. S2.** Scatter plots for limma and DESeq2 log fold change and adjusted p‐value comparisons.
**Fig. S3.** Overlap of disease‐associated gene signatures between Methods.
**Fig. S4.** Protein–protein interaction network centrality metrics for GBM and LIHC disease‐associated signature genes.
**Fig. S5.** Protein–protein interaction network centrality metrics for LUAD and PAAD disease‐associated signature genes.
**Fig. S6.** Protein–protein interaction network centrality metrics for top latent variable genes.
**Fig. S7.** Enriched gene set overlap for GBM, LIHC, LUAD, and PAAD.
**Fig. S8.** Heatmaps for LIHC GO_BP terms.
**Fig. S9.** GO heatmaps for LUAD.
**Fig. S10.** GO heatmaps for PAAD.
**Fig. S11.** DESeq2, limma, and transfer learning signature reversion results for GBM.
**Fig. S12.** DESeq2, limma, and transfer learning signature reversion results for LIHC.
**Fig. S13.** DESeq2, limma, and transfer learning signature reversion results for LUAD.
**Fig. S14.** DESeq2, limma, and transfer learning signature reversion results for PAAD.
**Fig. S15.** Alluvial plots of the top mechanism of action for identified GBM drug candidates.
**Fig. S16.** Alluvial plots of the top mechanism of action for identified LIHC drug candidates.
**Fig. S17.** Alluvial plots of the top mechanism of action for identified LUAD drug candidates.
**Fig. S18.** Alluvial plots of the top mechanism of action for identified PAAD drug candidates.
**Fig. S19.** Signature reversion NCS and FDR scatter plots from each disease‐associated gene signature for GBM.
**Fig. S20.** Signature reversion's NCS and FDR scatter plots for all methods for LIHC.
**Fig. S21.** Signature reversion's NCS and FDR scatter plots for all methods.
**Fig. S22.** Signature reversion's NCS and FDR scatter plots for all methods.
**Fig. S23.** A bar plot of the Spearman correlation of the normalized connectivity score (NCS) and the false discovery (FDR) between the different disease‐associated gene signature reversion results across the different cancers.
**Fig. S24.** Drug–drug similarity networks – drug structure.
**Fig. S25.** Screen for saxagliptin and icosapent growth inhibitory activity.
**Fig. S26.** Pamidronate and nimodipine decrease the growth of D456 cells.
**Fig. S27.** Pamidronate, but not Nimodipine, induces Apoptosis.
**Fig. S28.** Gene Expression of pamidronate and nimodipine drug targets in primary tumor and solid tissue normal samples.
**Fig. S29.** Principal component analysis for PAAD TCGA samples.
**Fig. S30.** Gene label switching test for transfer learning approach.

**Data S1.** Table of the number of samples for the project.

**Data S2.** Table of the permutation testing of random drug selection results for all methods across cancers.

**Data S3.** Pathway results for the top candidates for GBM.

**Data S4.** Computing system and package version information.

## Data Availability

The data that support the findings of this study are openly available in Recount3 at http://doi.org/doi:10.1101/2021.05.21.445138 [103], the Expanded CMAP LINCS Resource at https://clue.io/data/CMap2020#LINCS2020 [54], the Drugs@FDA at https://www.fda.gov/drugs/drug‐approvals‐and‐databases/drugsfda‐data‐files, PRISM and Achilles data in the Cancer Dependency Map (DepMap) portal at https://depmap.org/portal/depmap/ [106], and protein–protein interaction network from STRING at https://string‐db.org/cgi/download.pl [29].
