# Supplementary material for: Signature reversion of three disease‐associated gene signatures prioritizes cancer drug repurposing candidates
Source: FEBS Open Bio. 2024 Mar 26;14(5):803–30. doi: 10.1002/2211-5463.13796 (PMC11073506; doi:10.1002/2211-5463.13796)
Supplement: Supplementary file 1 — Fig. S1. Detailed overview of the signature reversion with three disease‐associated gene signatures. Fig. S2. Scatter plots for limma and DESeq2 log fold change and adjusted p‐value comparisons. Fig. S3. Overlap of disease‐associated gene signatures between Methods. Fig. S4. Protein–protein interaction network centrality metrics for GBM and LIHC disease‐associated signature genes. Fig. S5. Protein–protein interaction network centrality metrics for LUAD and PAAD disease‐associated signature genes. Fig. S6. Protein–protein interaction network centrality metrics for top latent variable genes. Fig. S7. Enriched gene set overlap for GBM, LIHC, LUAD, and PAAD. Fig. S8. Heatmaps for LIHC GO_BP terms. Fig. S9. GO heatmaps for LUAD. Fig. S10. GO heatmaps for PAAD. Fig. S11. DESeq2, limma, and transfer learning signature reversion results for GBM. Fig. S12. DESeq2, limma, and transfer learning signature reversion results for LIHC. Fig. S13. DESeq2, limma, and transfer learning signature reversion results for LUAD. Fig. S14. DESeq2, limma, and transfer learning signature reversion results for PAAD. Fig. S15. Alluvial plots of the top mechanism of action for identified GBM drug candidates. Fig. S16. Alluvial plots of the top mechanism of action for identified LIHC drug candidates. Fig. S17. Alluvial plots of the top mechanism of action for identified LUAD drug candidates. Fig. S18. Alluvial plots of the top mechanism of action for identified PAAD drug candidates. Fig. S19. Signature reversion NCS and FDR scatter plots from each disease‐associated gene signature for GBM. Fig. S20. Signature reversion's NCS and FDR scatter plots for all methods for LIHC. Fig. S21. Signature reversion's NCS and FDR scatter plots for all methods. Fig. S22. Signature reversion's NCS and FDR scatter plots for all methods. Fig. S23. A bar plot of the Spearman correlation of the normalized connectivity score (NCS) and the false discovery (FDR) between the different disease‐associated gene signature rev [file FEB4-14-803-s004.docx]

# **Supplemental Figures**


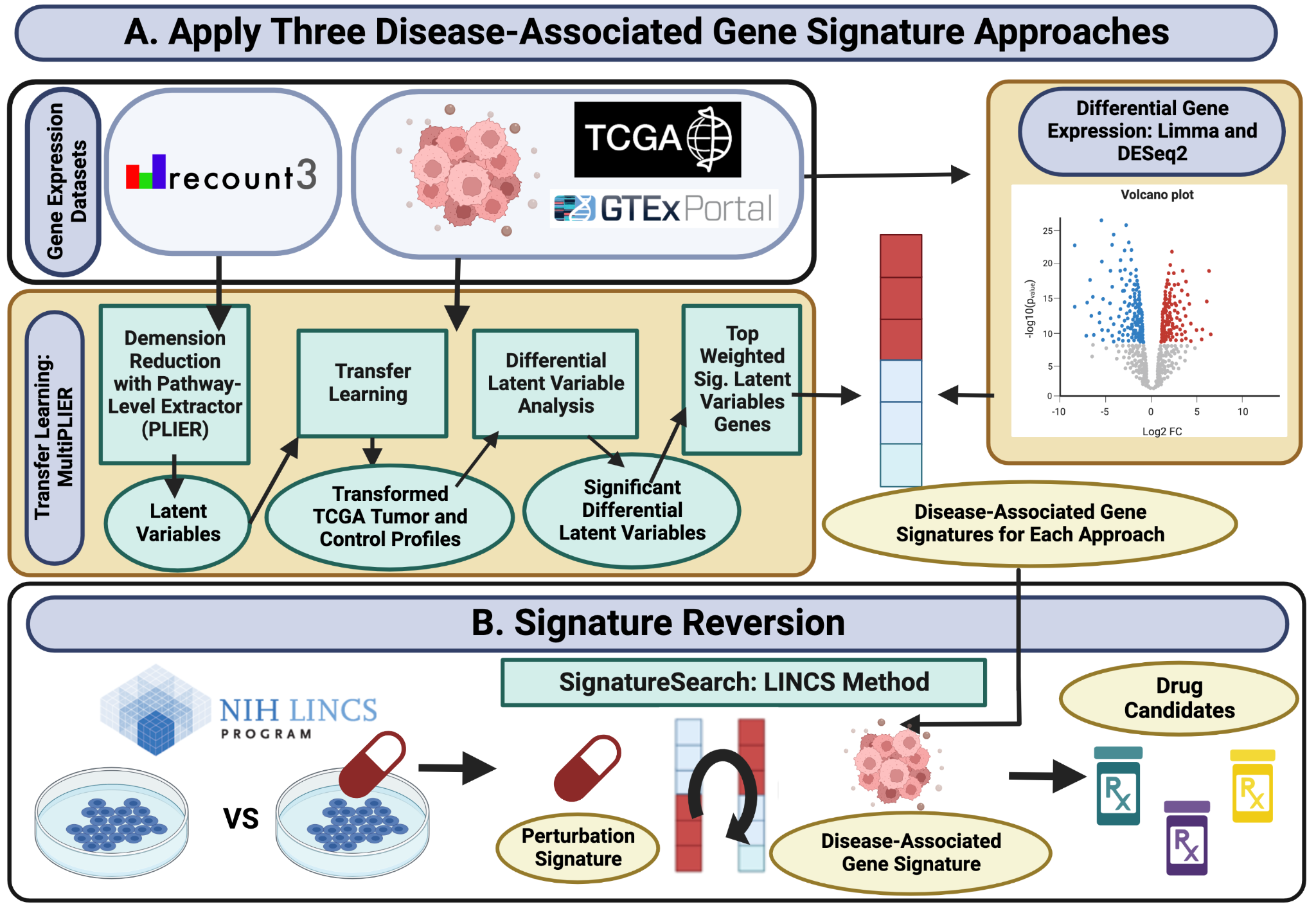


**Supplemental Figure 1: Detailed overview of the signature reversion with three disease-associated gene signatures. A)** The process for making the disease-associated gene signatures for the three approaches. **B)** The signature reversion methodology.

**
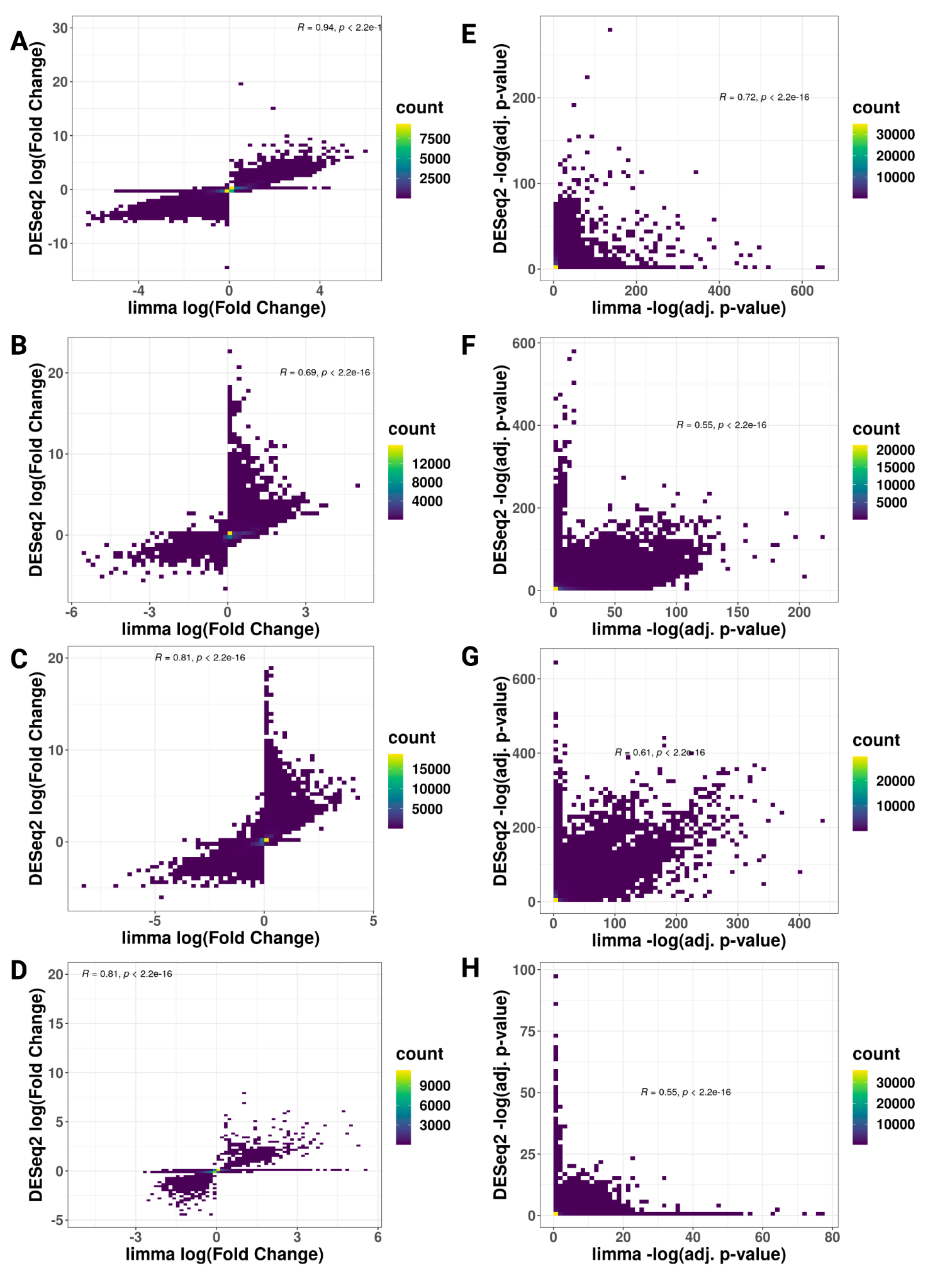
**

**Supplemental Figure 2: Scatter plots for limma and DESeq2 log fold change and adjusted p-value comparisons. A-D)** Scatter plot of the limma log fold change and DESeq2 log fold change for GBM, LIHC, LUAD, and PAAD, respectively. **E-H)** Scatter plot of the limma adjusted p-value and DESeq2 adjusted p-value for GBM, LIHC, LUAD, and PAAD, respectively. Spearman correlation and p-value from linear regression models are also plotted on each panel.

**
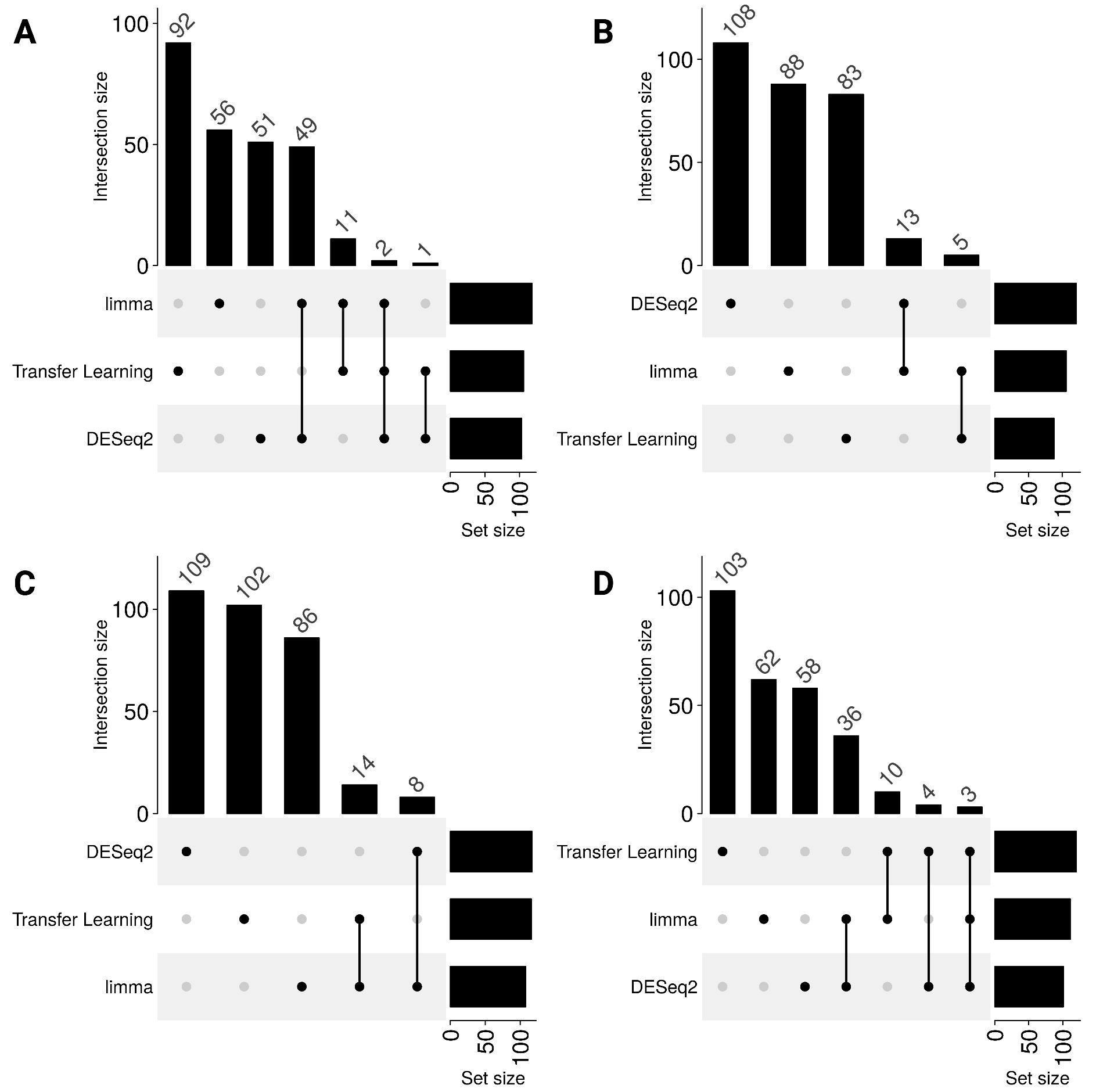
**

**Supplemental Figure 3: Overlap of disease-associated gene signatures between Methods. A-D)** Upset plots of the overlap between disease-associated gene signatures for GBM, LIHC, LUAD, and PAAD, respectively.

**
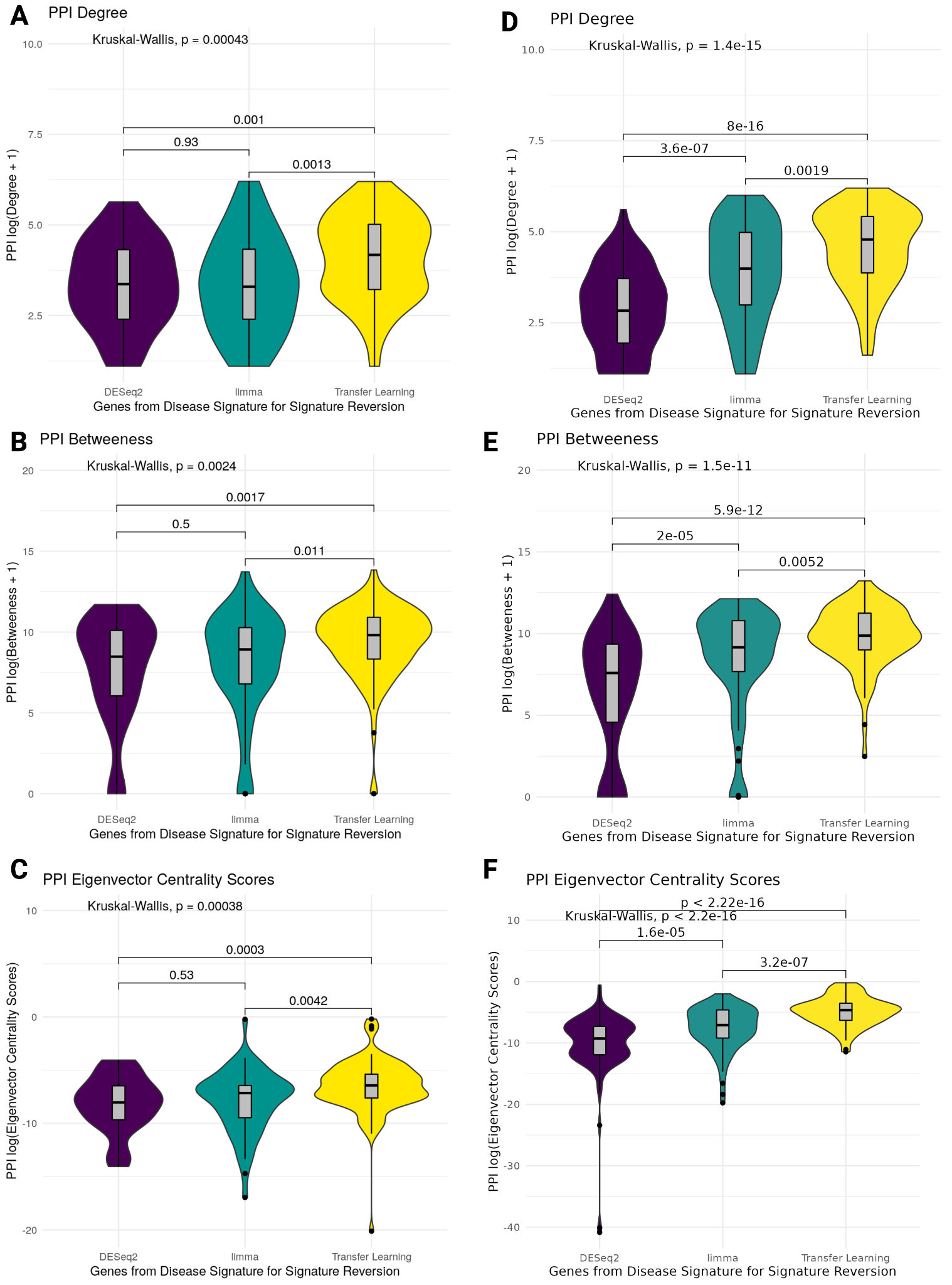
**

**Supplemental Figure 4: Protein-protein interaction network centrality metrics for GBM and LIHC disease-associated signature genes.** Violin plots for **A)** degree, **B)** betweenness, and **C)** eigenvector centrality for GBM and **D)** degree, **E)** betweenness, and **F)** eigenvector centrality for LIHC. All tests for this figure are Kruskal-Wallis and Wilcox tests with a Bonferroni multiple hypothesis adjustment.


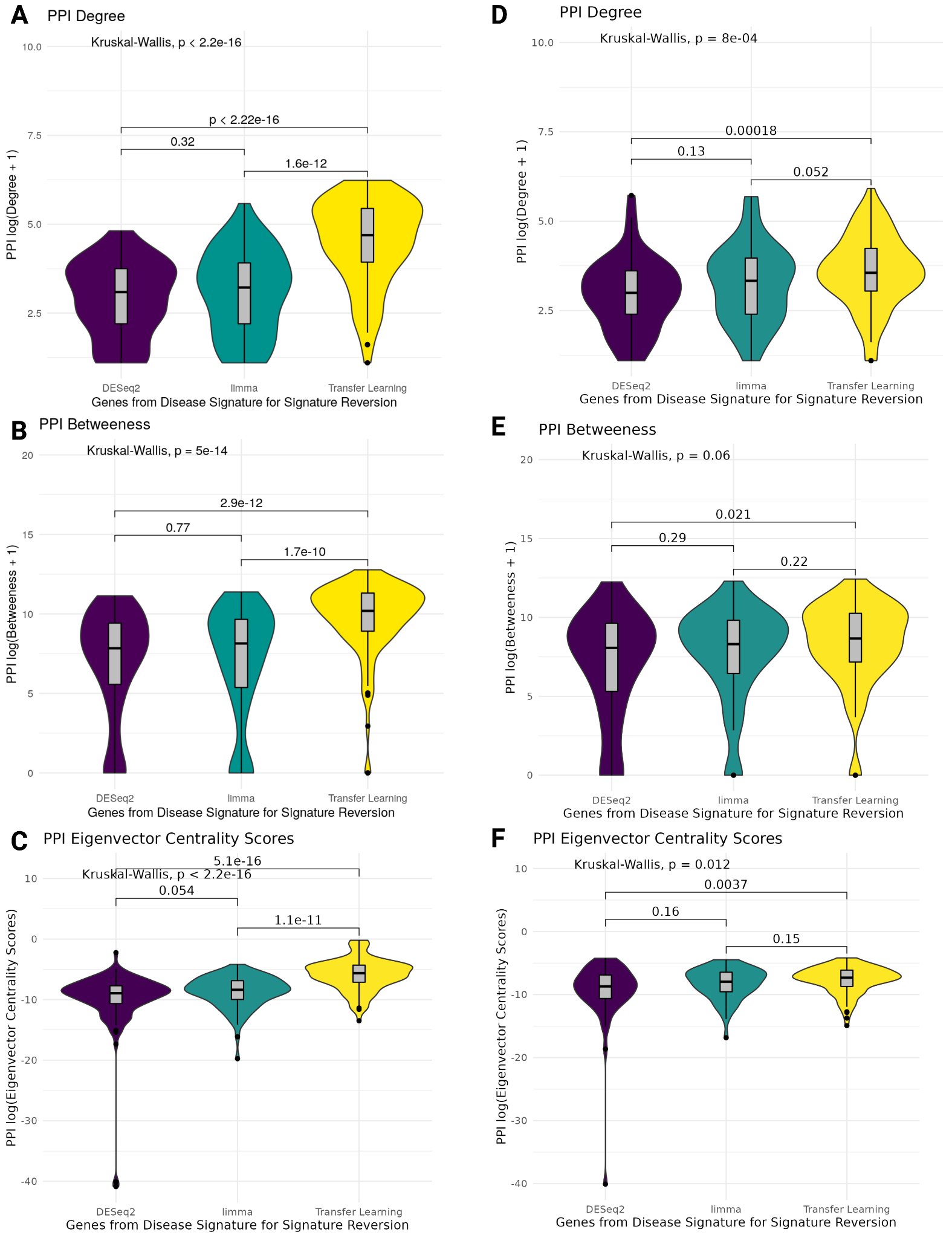


**Supplemental Figure 5: Protein-protein interaction network centrality metrics for LUAD and PAAD disease-associated signature genes.** Violin plots for **A)** degree, **B)** betweenness, and **C)** eigenvector centrality for LUAD and **D)** degree, **E)** betweenness, and **F)** eigenvector centrality for PAAD. All tests for this figure are Kruskal-Wallis and Wilcox tests with a Bonferroni multiple hypothesis adjustment.

**
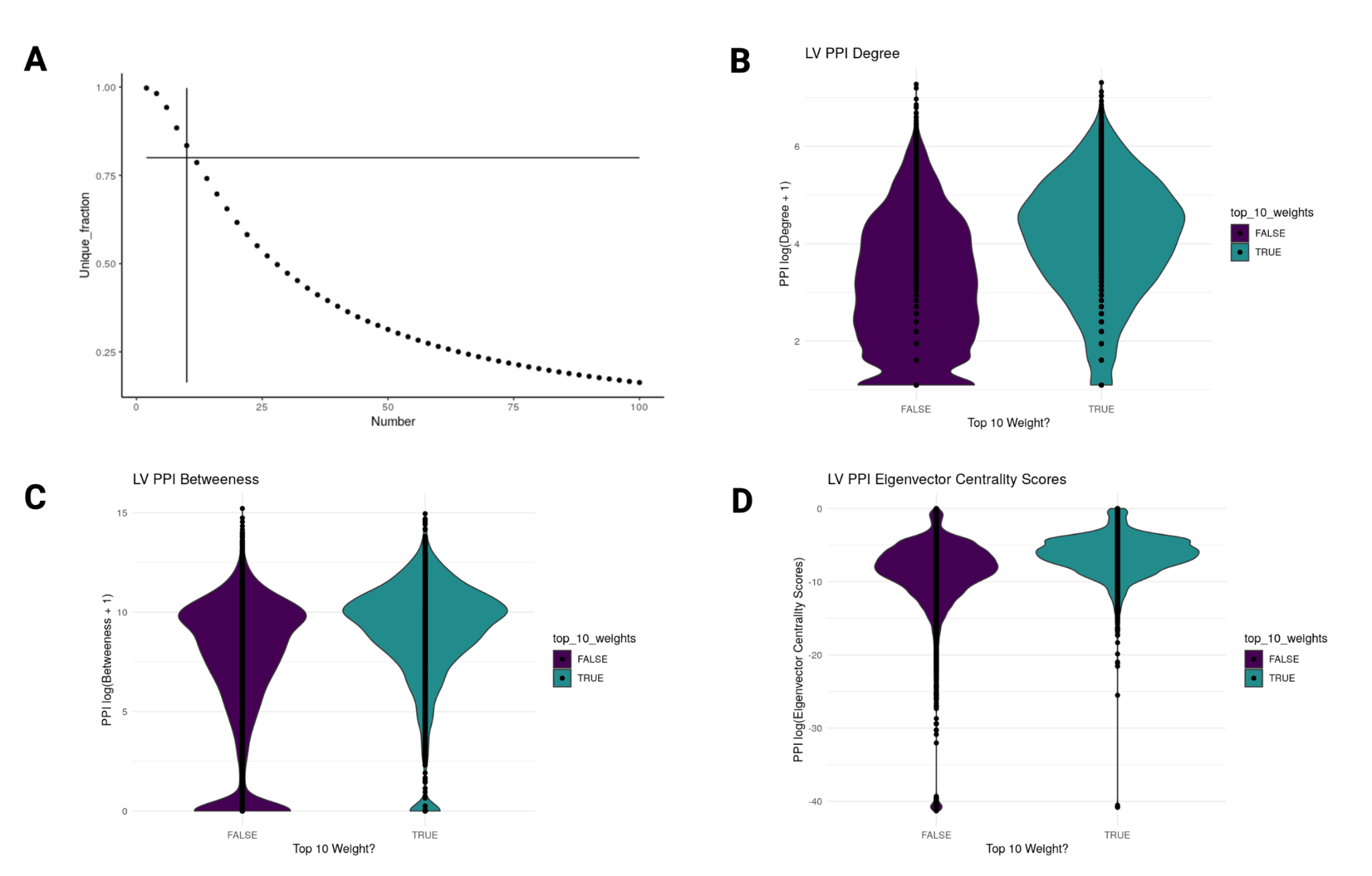
**

**Supplemental Figure 6: Protein-protein interaction network centrality metrics for top latent variable genes. A)** Ratio of the number of genes in the top most weighted genes of all latent variables to all of the genes included in the top most weighted genes of all latent variables (y-axis) plotted by a given number of top most weighted genes (x-axis). The vertical line is at 10 (i.e., 10 genes each for the 385 latent variables) and the horizontal line at 0.80 was the cut-off for inclusion in the network centrality analysis. **B)** PPI degree violin plot (adj. P-value = 0). **C)** PPI betweenness (adj. P-value = 1.883475e-176). **D)** PPI eigenvector centrality (adj. P-value = 1.231573e-231). All statistical tests for this figure are Wilcox tests with a Bonferroni multiple hypothesis adjustment.


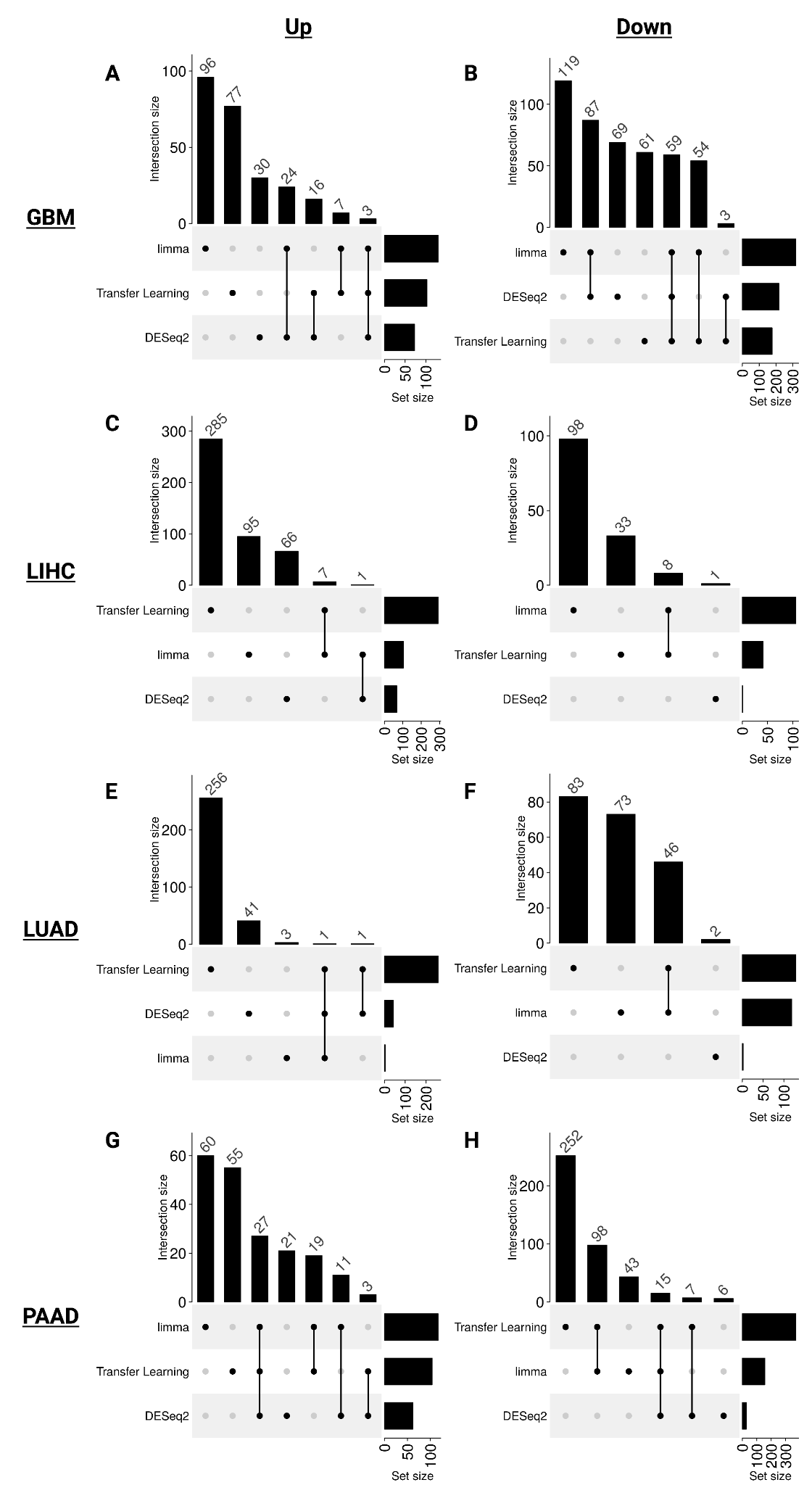


**Supplemental Figure 7: Enriched gene set overlap for GBM, LIHC, LUAD, and PAAD. A, C, E, G)** Up and **B, D, F, H)** down-regulated gene set overlap from the functional enrichment results for each disease-associated gene signature for GBM, LIHC, LUAD, and PAAD, respectively.

**
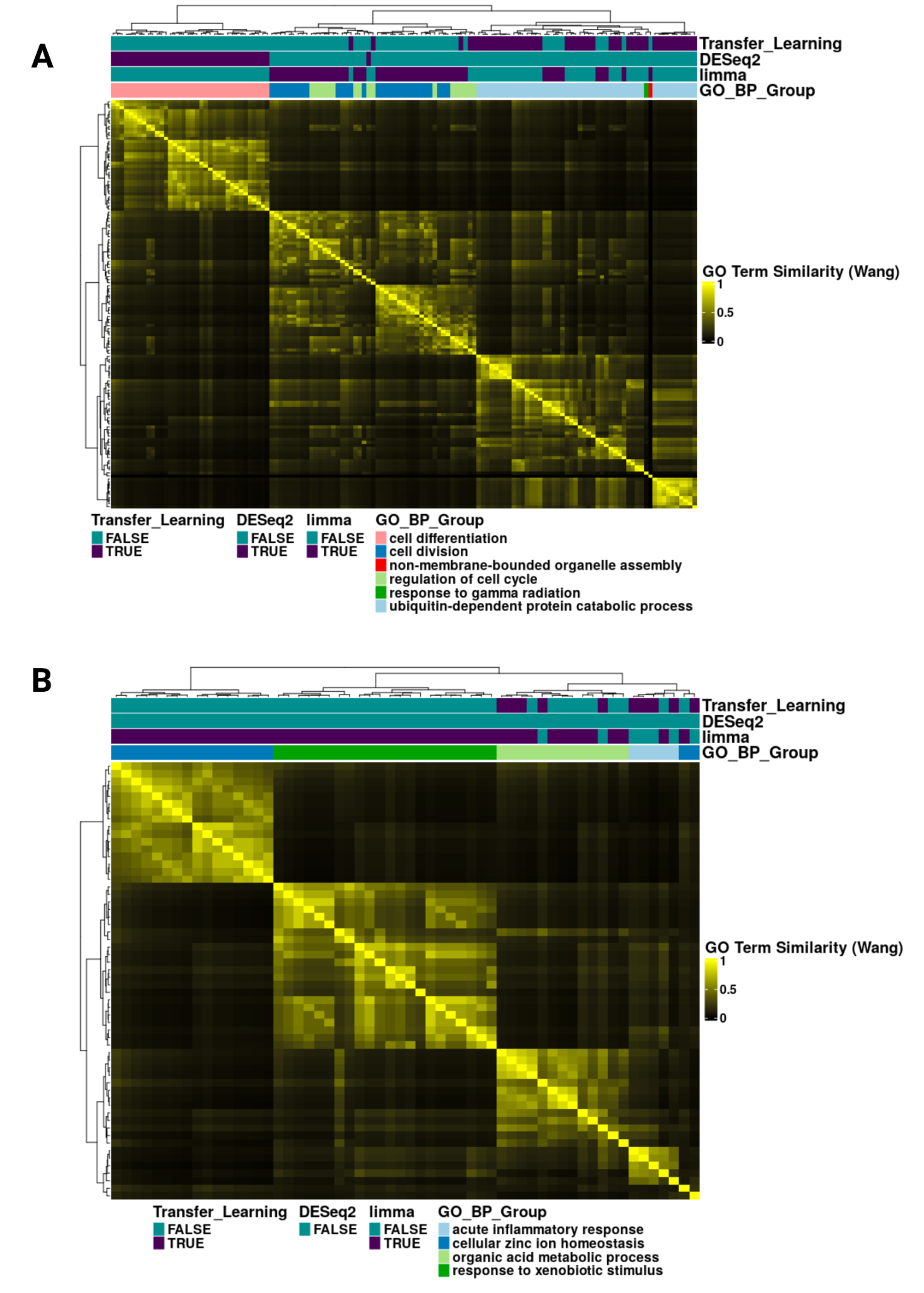
**

**Supplemental Figure 8: Heatmaps for LIHC GO_BP terms. A)** Heatmap of the Gene Ontology (GO) term semantic similarity (Wang method) of the up-regulated enriched GO Biological Process terms from the LIHC disease-associated gene signatures. Each term is associated with a disease-associated gene signature if the row for that method is purple. In addition, the Gene Ontology Biological Process terms were grouped together based on common parent terms, and the different parent term groups are indicated in the GO_BP_Group. **B)** A heatmap of the GO term semantic similarity (Wang method) of down-regulated enriched Gene Ontology Biological Process terms for LIHC.


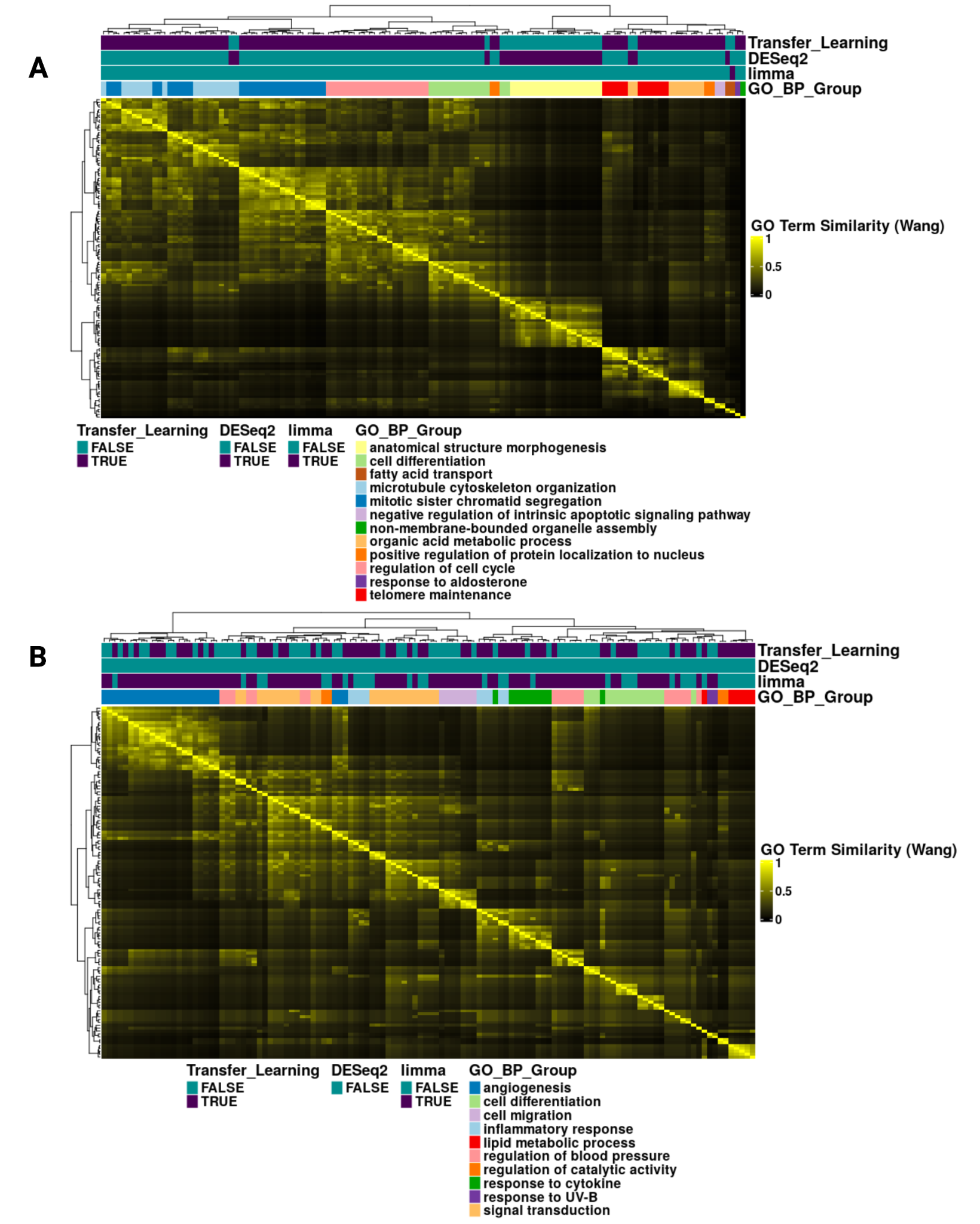


**Supplemental Figure 9: GO heatmaps for LUAD. A)** Heatmap of the Gene Ontology (GO) term semantic similarity (Wang method) of the up-regulated enriched GO Biological Process terms from the LUAD disease-associated gene signatures. Each term is associated with a disease-associated gene signature if the row for that method is purple. In addition, the Gene Ontology Biological Process terms were grouped together based on common parent terms, and the different groups are indicated in the GO_BP_Group. **B)** A heatmap of the GO term semantic similarity (Wang method) of down-regulated enriched Gene Ontology Biological Process terms for LUAD.

**
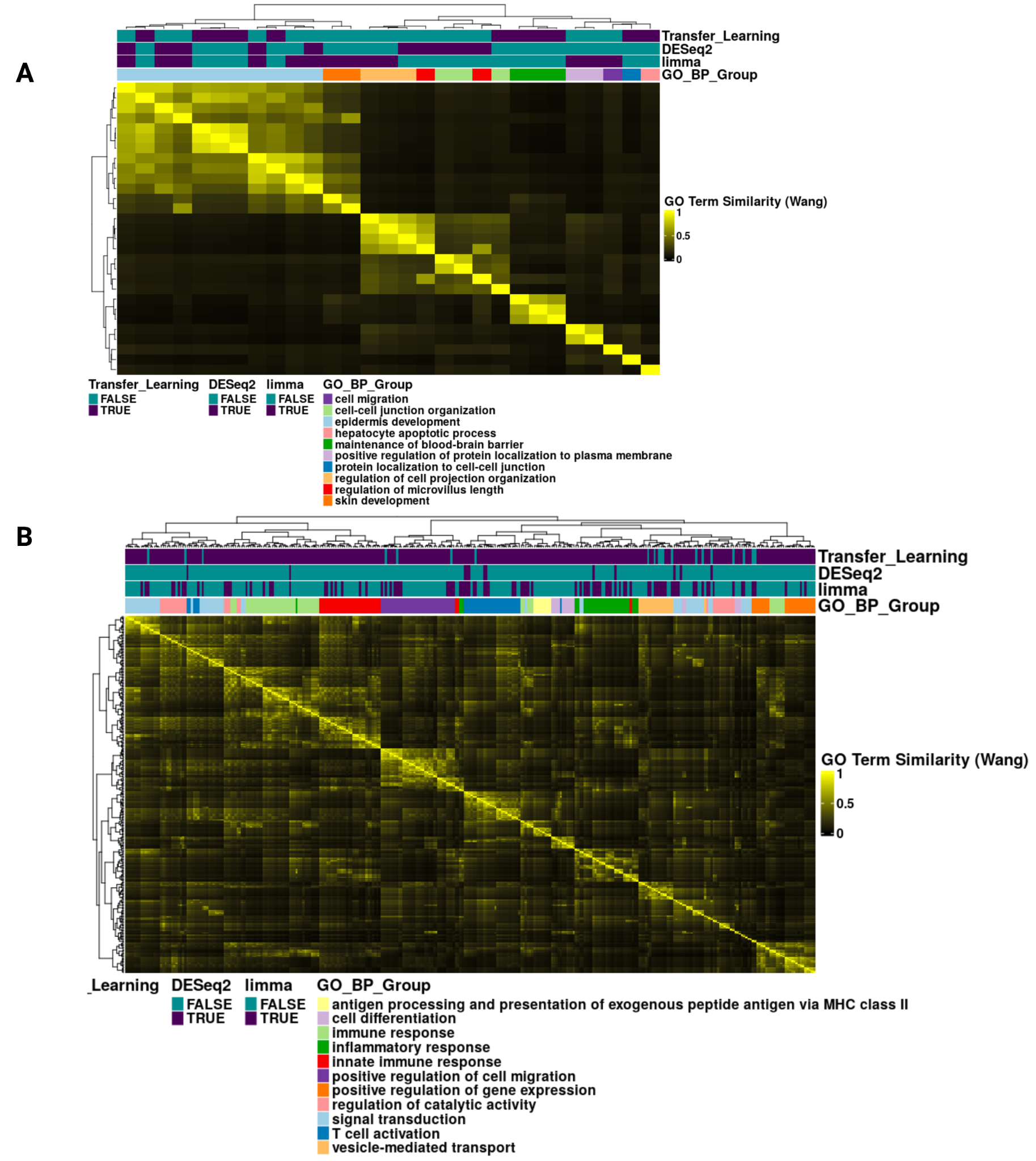
**

**Supplemental Figure 10: GO heatmaps for PAAD. A)** Heatmap of the Gene Ontology (GO) term semantic similarity (Wang method) of the up-regulated enriched GO Biological Process terms from the PAAD disease-associated gene signatures. Each term is associated with a disease-associated gene signature if the row for that method is purple. In addition, the Gene Ontology Biological Process terms were grouped together based on common parent terms, and the different groups are indicated in the GO_BP_Group. **B)** A heatmap of the GO term semantic similarity (Wang method) of down-regulated enriched Gene Ontology Biological Process terms for PAAD.

**
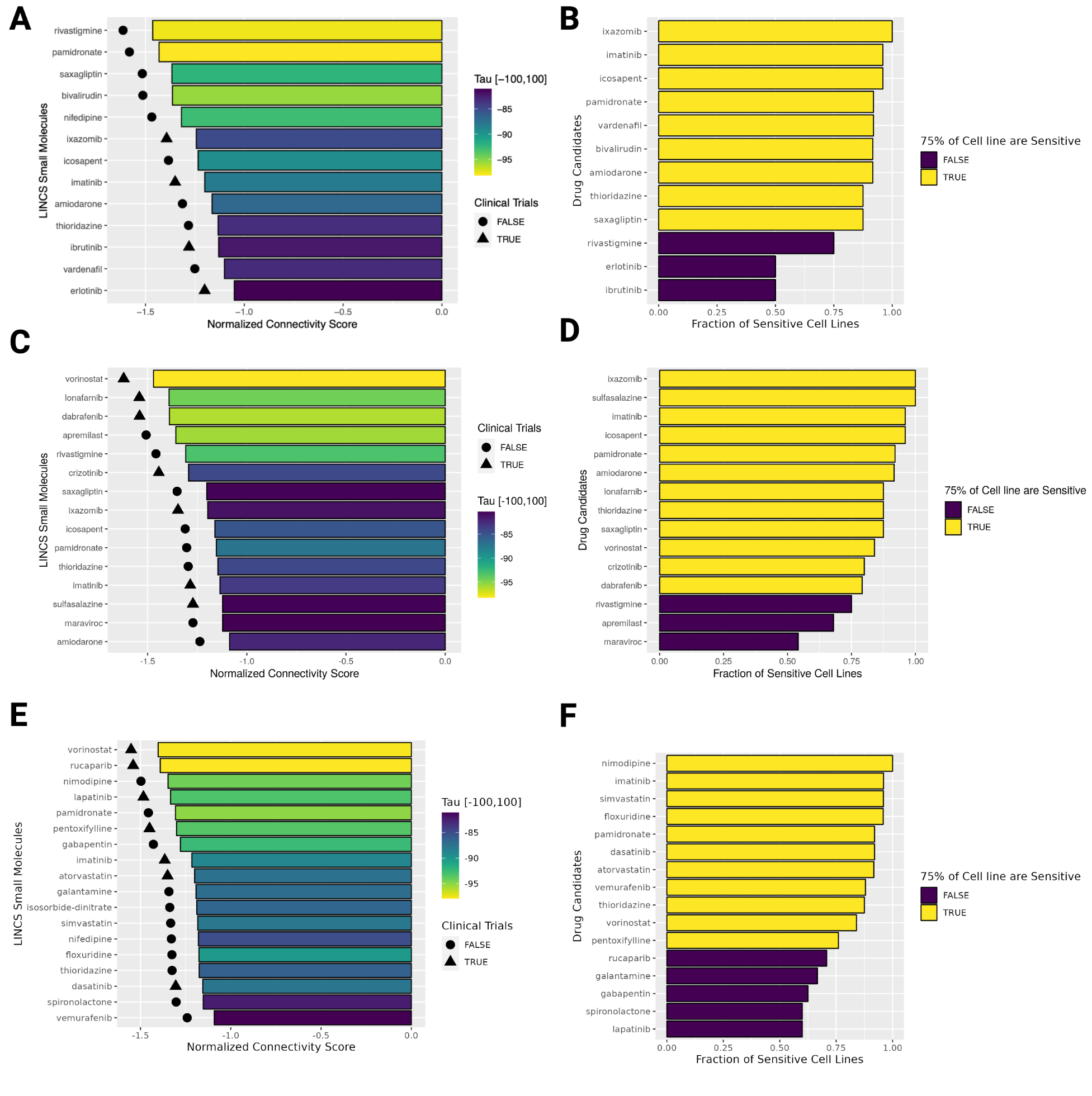
**

**Supplemental Figure 11: DESeq2, limma, and transfer learning signature reversion results for GBM. A)** Barplot of the top drug repurposing candidates ordered by Normalized Connectivity Scores and with a -80 Tau cutoff for the DESeq2 disease-associated signature. **B)** Barplot of the fraction of sensitive cell lines in the PRISM dataset for DESeq2 disease-associated signature drug candidates. **C)** Barplot of the top drug repurposing candidates identified from the limma disease-associated signature. **D)** Barplot of the fraction of sensitive cell lines in the PRISM dataset for limma disease-associated signature drug candidates. **E)** Barplot of the top drug repurposing candidates identified from the transfer learning disease-associated signature. **F)** Barplot of the fraction of sensitive cell lines in the PRISM dataset for transfer learning disease-associated signature drug candidates.

**
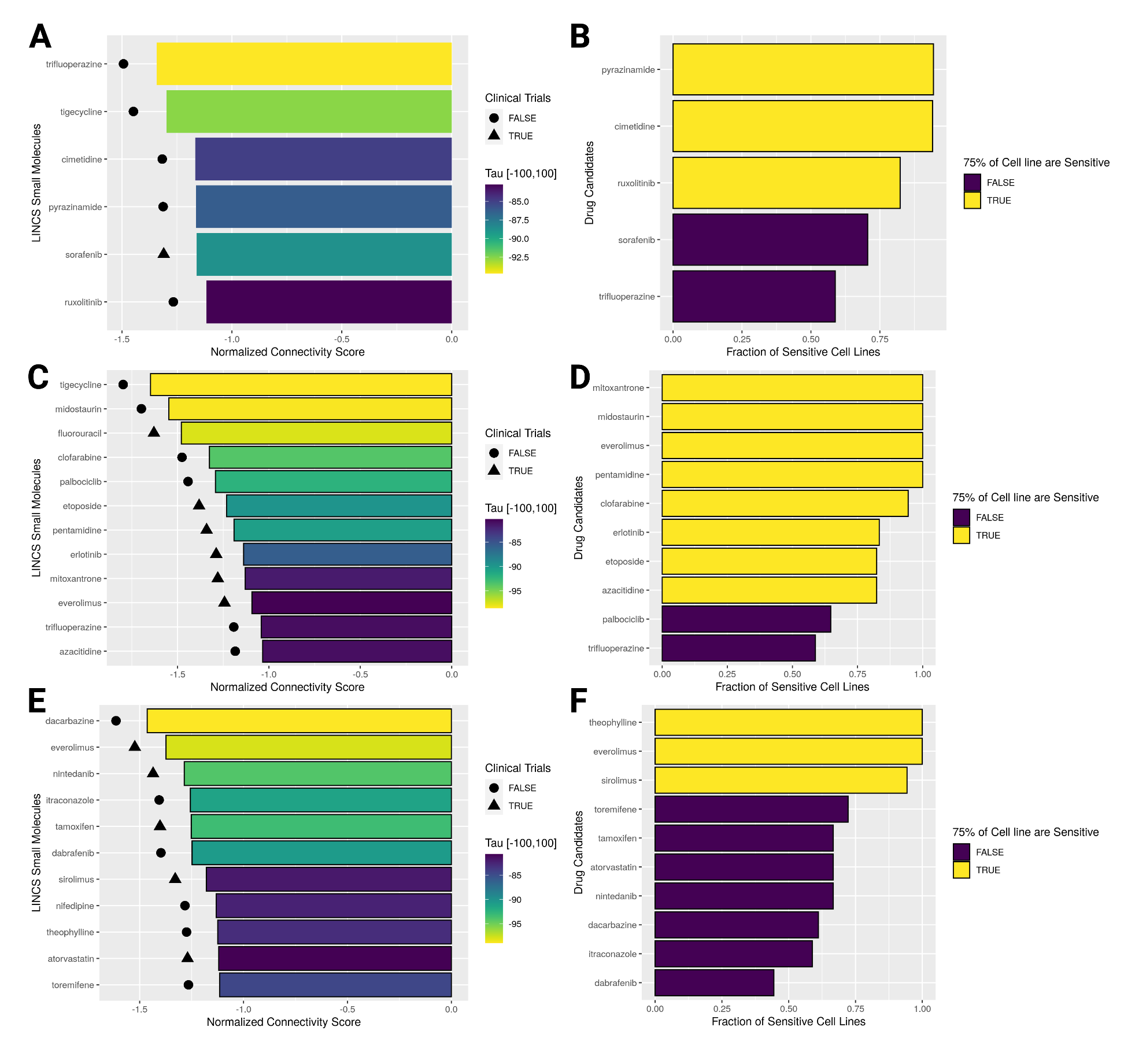
**

**Supplemental Figure 12: DESeq2, limma, and transfer learning signature reversion results for LIHC. A)** Barplot of the top drug repurposing candidates ordered by Normalized Connectivity Scores and with a -80 Tau cutoff for the DESeq2 disease-associated signature. **B)** Barplot of the fraction of sensitive cell lines in the PRISM dataset for DESeq2 disease-associated signature drug candidates. **C)** Barplot of the top drug repurposing candidates identified from the limma disease-associated signature. **D)** Barplot of the fraction of sensitive cell lines in the PRISM dataset for limma disease-associated signature drug candidates. **E)** Barplot of the top drug repurposing candidates identified from the transfer learning disease-associated signature. **F)** Barplot of the fraction of sensitive cell lines in the PRISM dataset for transfer learning disease-associated signature drug candidates.

**
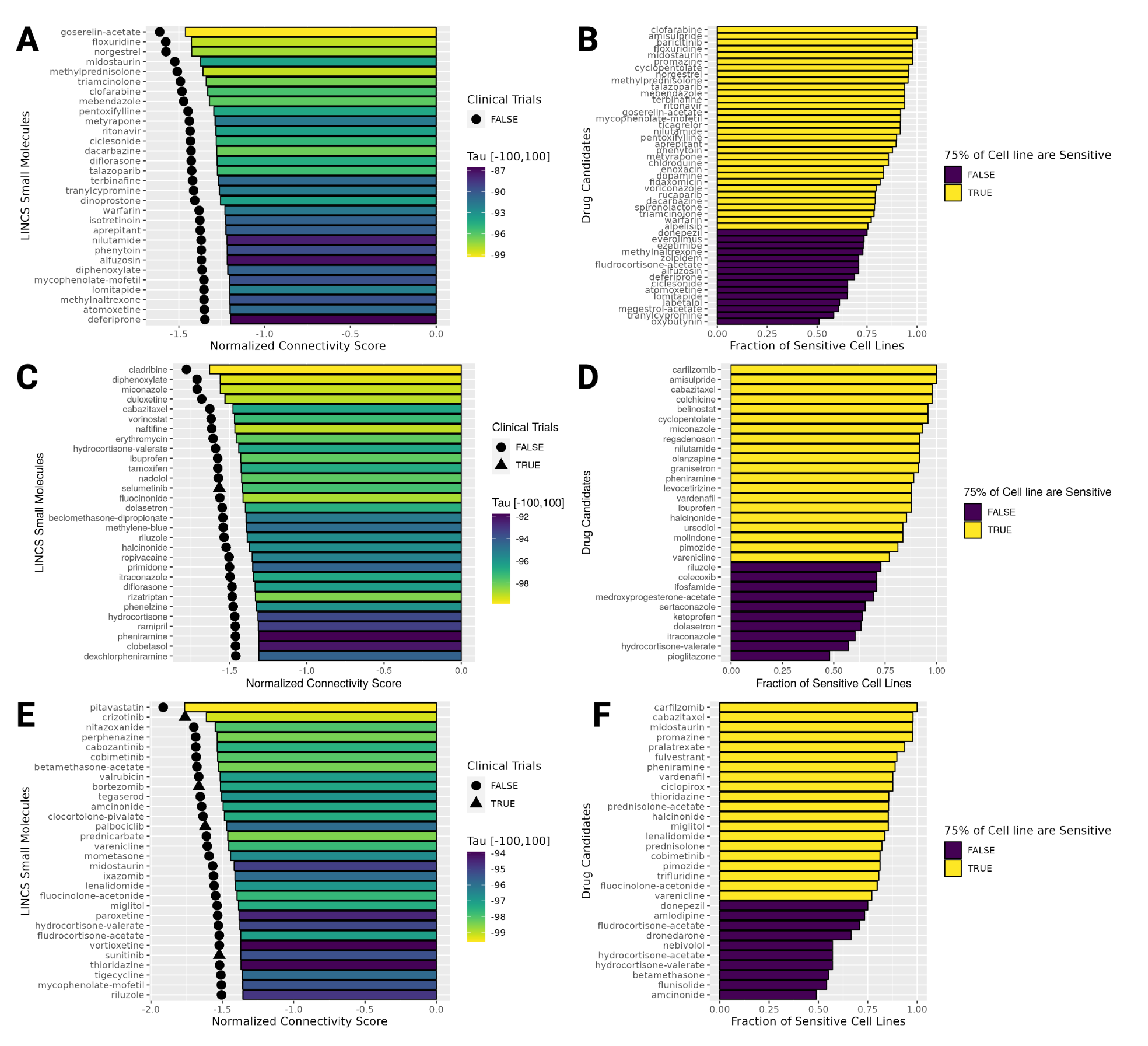
**

**Supplemental Figure 13: DESeq2, limma, and transfer learning signature reversion results for LUAD. A)** Barplot of the top drug repurposing candidates ordered by Normalized Connectivity Scores and with a -80 Tau cutoff for the DESeq2 disease-associated signature. **B)** Barplot of the fraction of sensitive cell lines in the PRISM dataset for DESeq2 disease-associated signature drug candidates. **C)** Barplot of the top drug repurposing candidates identified from the limma disease-associated signature. **D)** Barplot of the fraction of sensitive cell lines in the PRISM dataset for limma disease-associated signature drug candidates. **E)** Barplot of the top drug repurposing candidates identified from the transfer learning disease-associated signature. **F)** Barplot of the fraction of sensitive cell lines in the PRISM dataset for transfer learning disease-associated signature drug candidates.

**
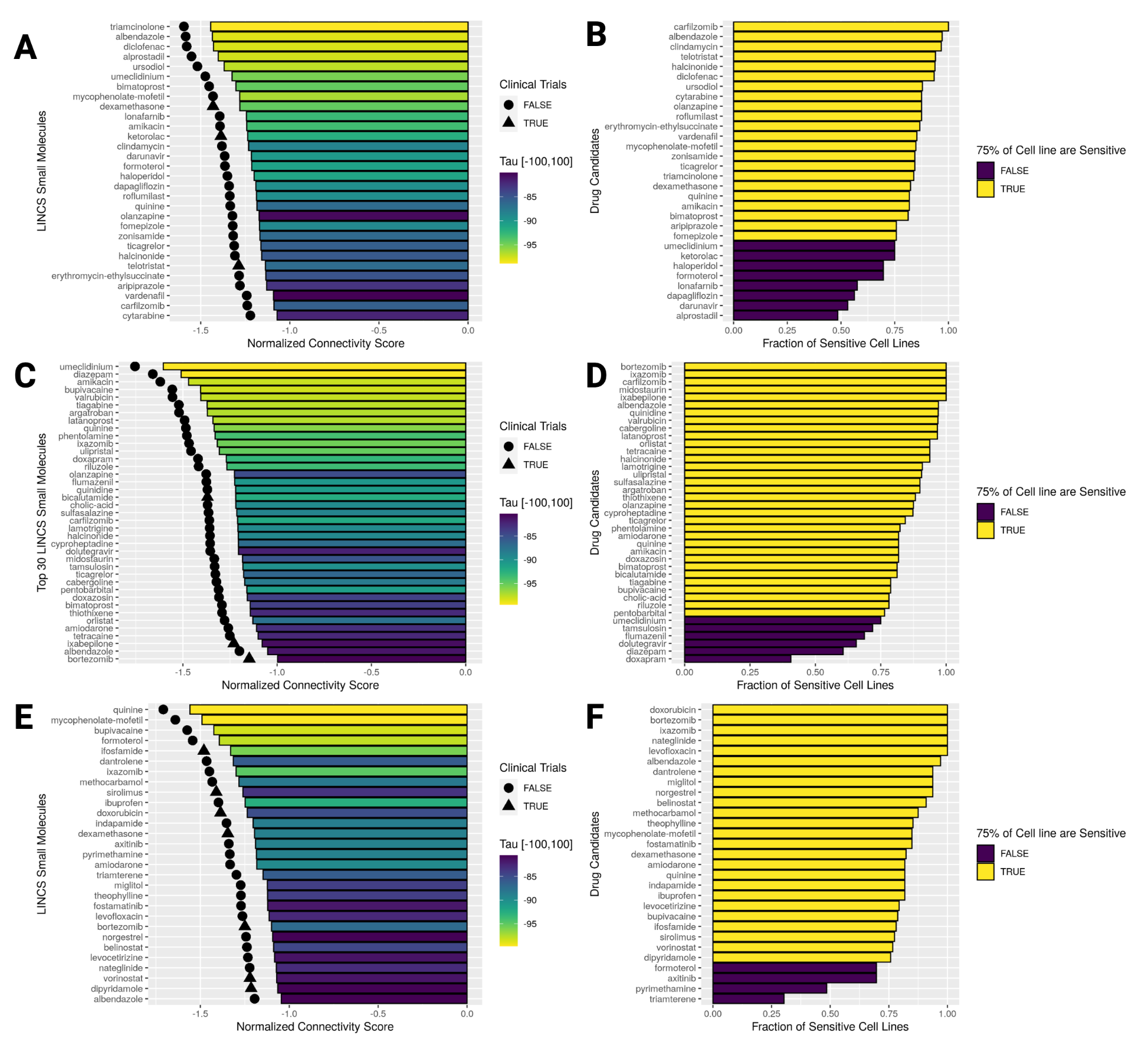
**

**Supplemental Figure 14: DESeq2, limma, and transfer learning signature reversion results for PAAD. A)** Barplot of the top drug repurposing candidates ordered by Normalized Connectivity Scores and with a -80 Tau cutoff for the DESeq2 disease-associated signature. **B)** Barplot of the fraction of sensitive cell lines in the PRISM dataset for DESeq2 disease-associated signature drug candidates. **C)** Barplot of the top drug repurposing candidates identified from the limma disease-associated signature. **D)** Barplot of the fraction of sensitive cell lines in the PRISM dataset for limma disease-associated signature drug candidates. **E)** Barplot of the top drug repurposing candidates identified from the transfer learning disease-associated signature. **F)** Barplot of the fraction of sensitive cell lines in the PRISM dataset for transfer learning disease-associated signature drug candidates.

**
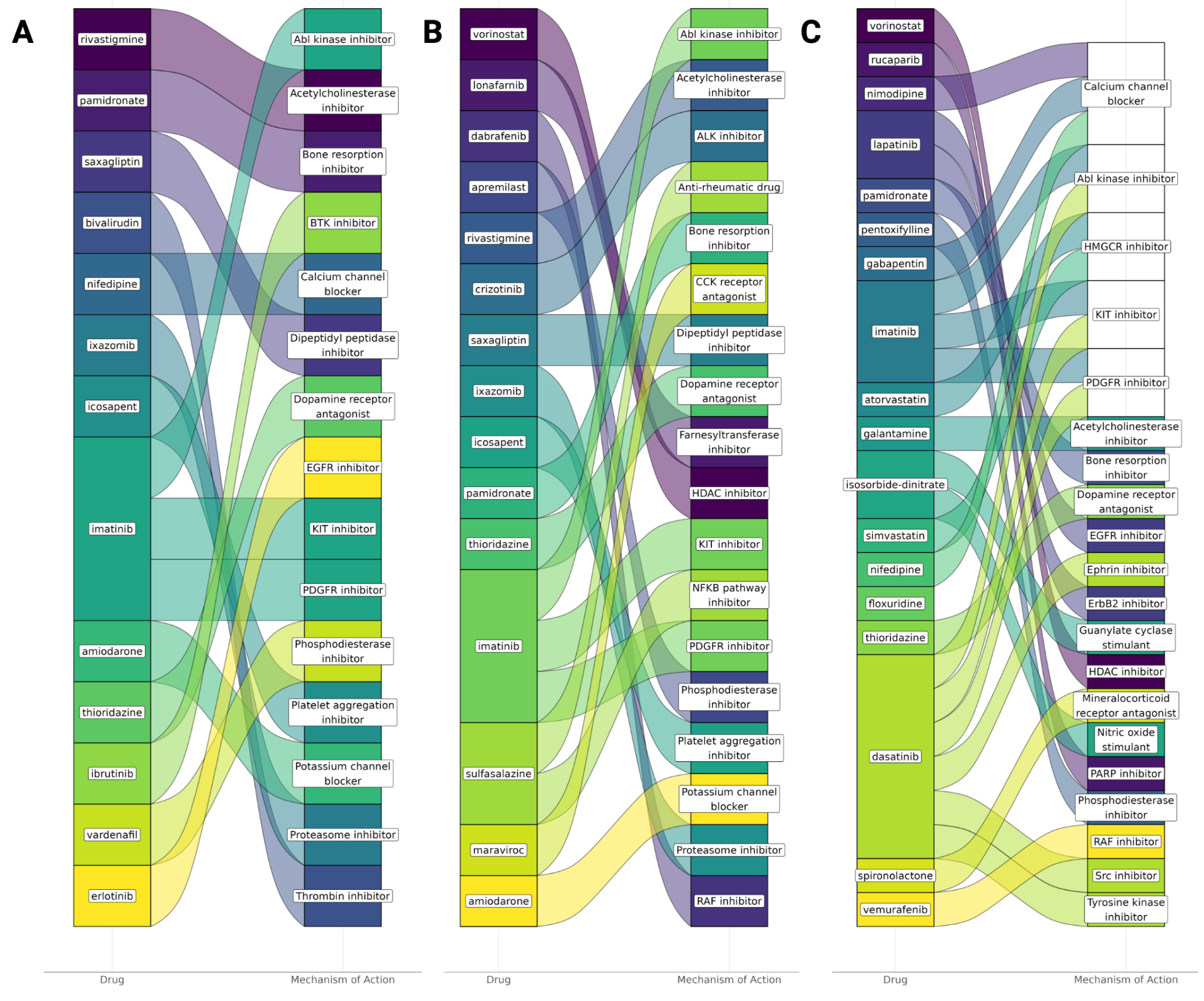
**

**Supplemental Figure 15: Alluvial plots of the top mechanism of action for identified GBM drug candidates. A-C)** Alluvial plots for GBM drug candidates identified from the DESeq2, limma, or transfer learning disease-associated signatures, respectively. If the mechanism of action is colored white, this mechanism of action is shared by multiple drugs while the colored mechanism of action matches the drug with the same color. Note that these colors do not correspond to the same drug across disease-associated signatures or across cancer figures.


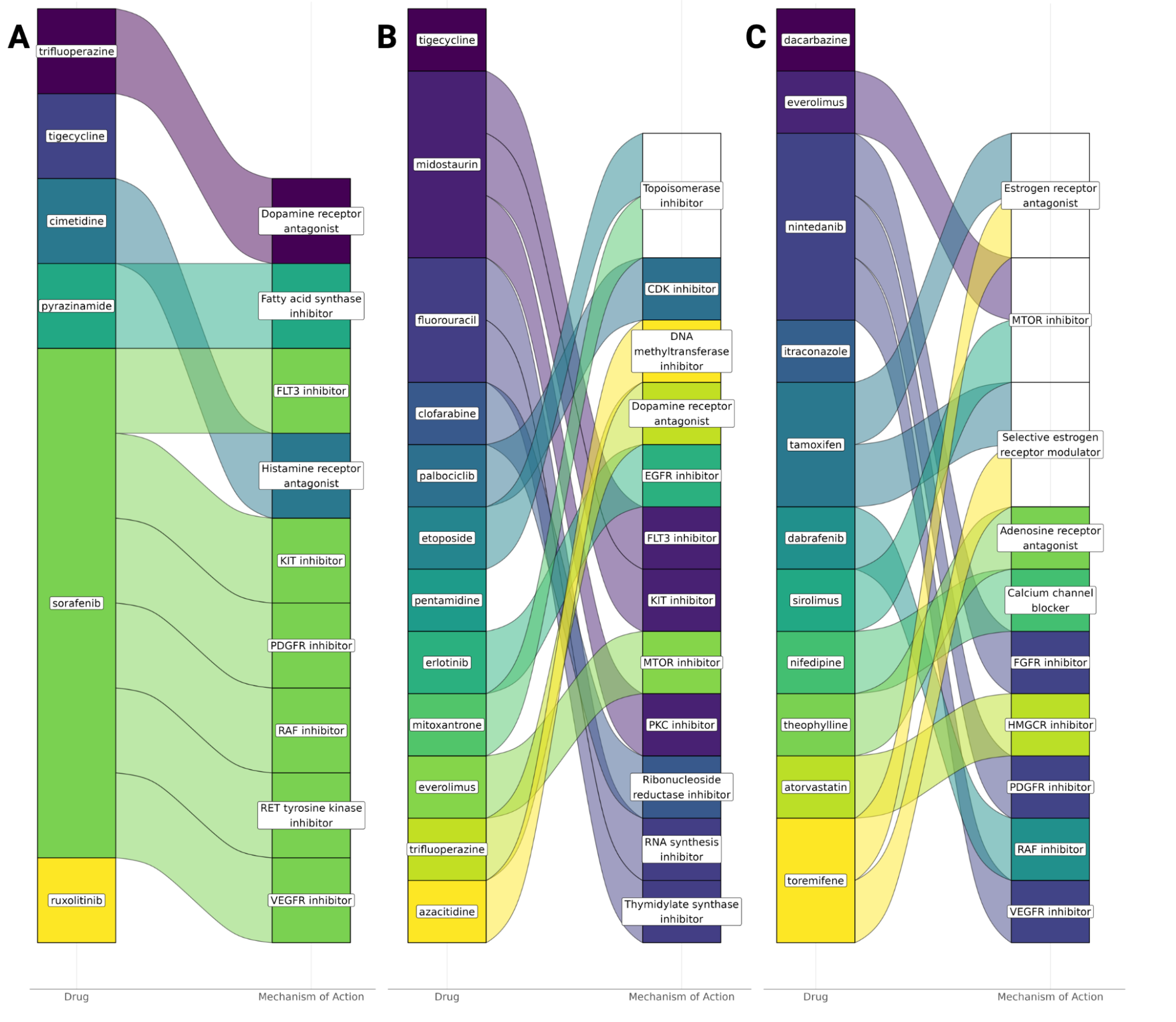


**Supplemental Figure 16: Alluvial plots of the top mechanism of action for identified LIHC drug candidates. A-C)** Alluvial plots for LIHC drug candidates identified from the DESeq2, limma, or transfer learning disease-associated signatures, respectively. If the mechanism of action is colored white, this mechanism of action is shared by multiple drugs while the colored mechanism of action matches the drug with the same color. Note that these colors do not correspond to the same drug across disease-associated signatures or across cancer figures.

**
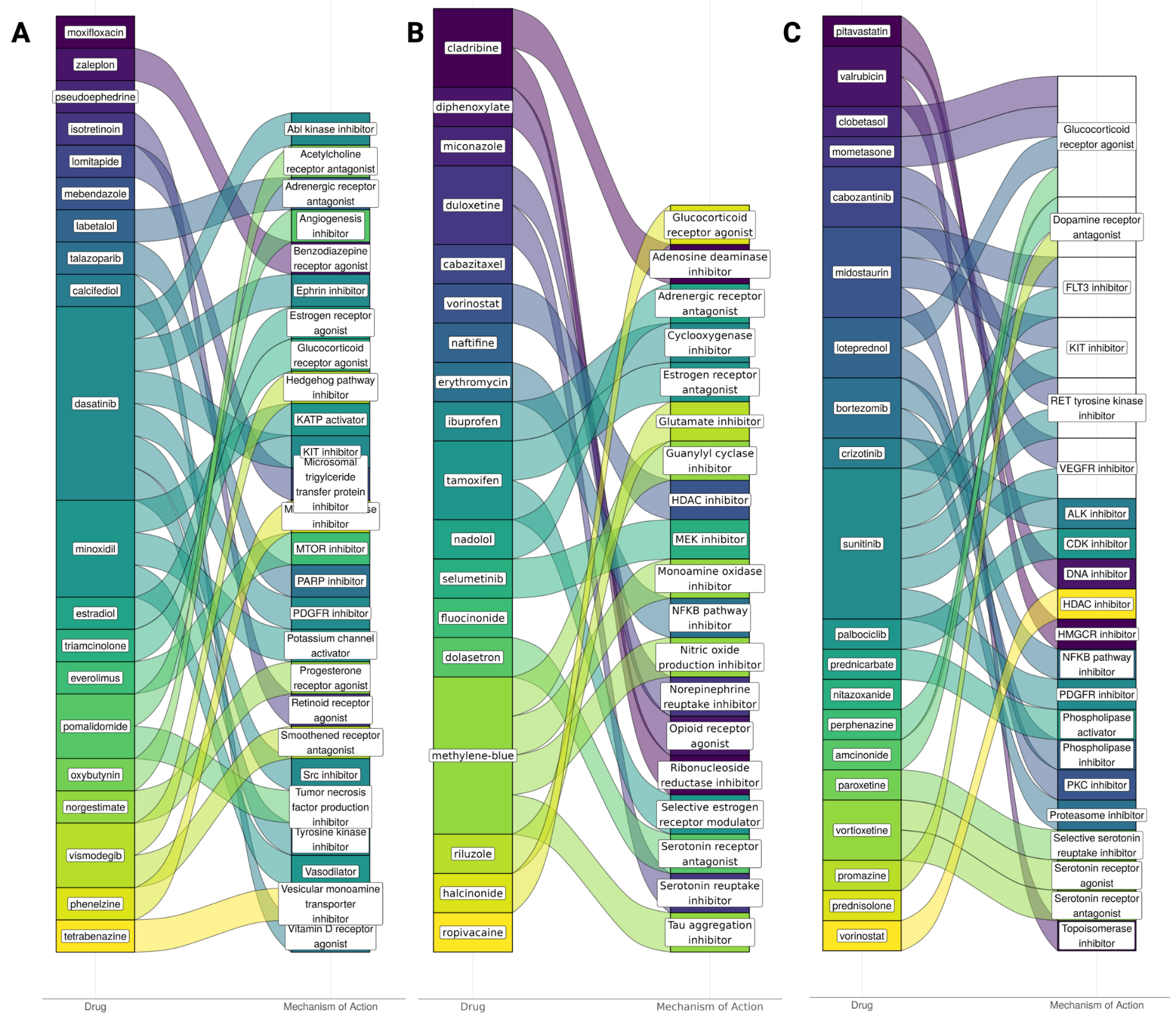
**

**Supplemental Figure 17: Alluvial plots of the top mechanism of action for identified LUAD drug candidates. A-C)** Alluvial plots for LUAD drug candidates identified from the DESeq2, limma, or transfer learning disease-associated signatures, respectively. If the mechanism of action is colored white, this mechanism of action is shared by multiple drugs while the colored mechanism of action matches the drug with the same color. Note that these colors do not correspond to the same drug across disease-associated signatures or across cancer figures.


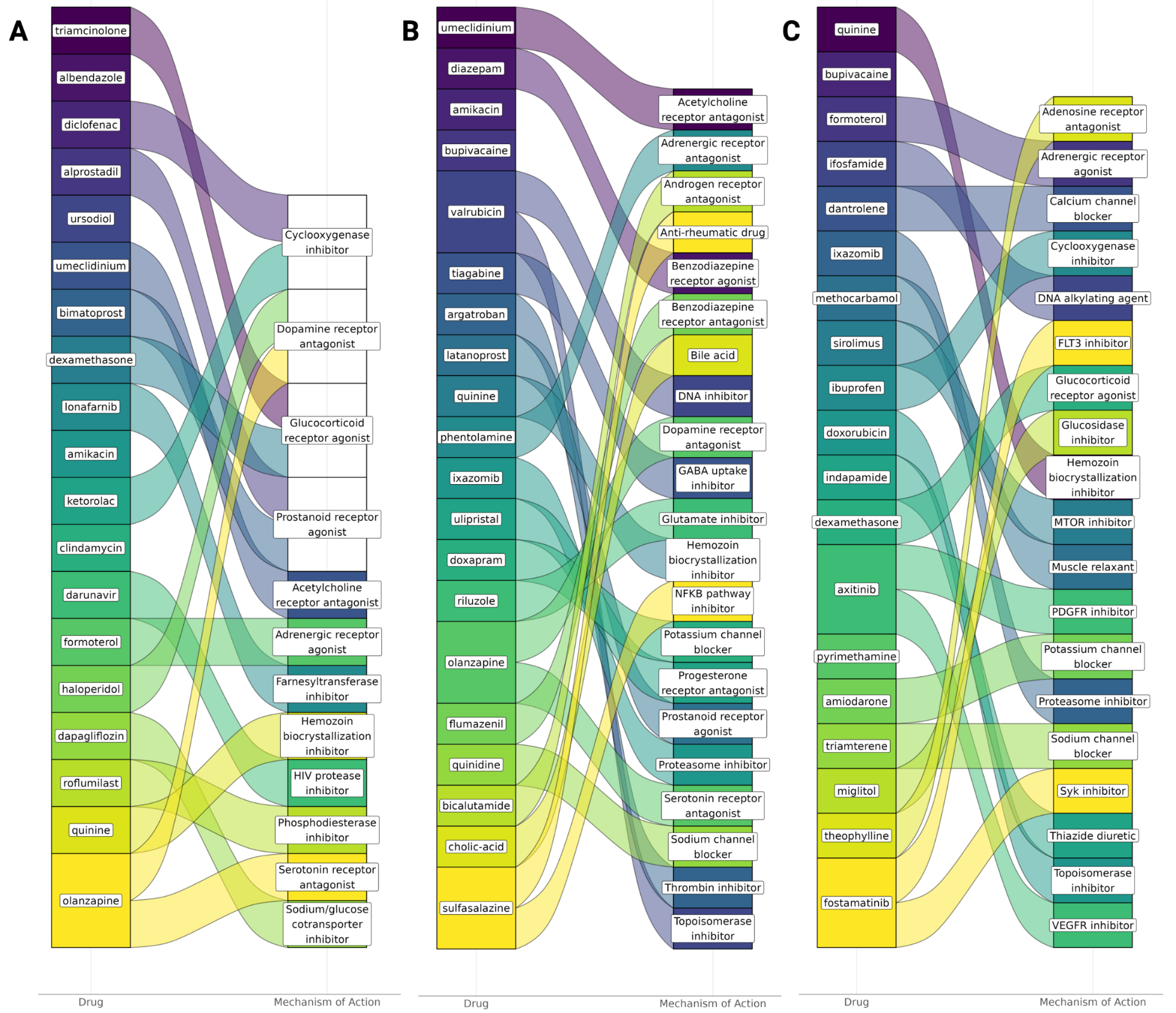


**Supplemental Figure 18: Alluvial plots of the top mechanism of action for identified PAAD drug candidates. A-C)** Alluvial plots for PAAD drug candidates identified from the DESeq2, limma, or transfer learning disease-associated signatures, respectively. If the mechanism of action is colored white, this mechanism of action is shared by multiple drugs while the colored mechanism of action matches the drug with the same color. Note that these colors do not correspond to the same drug across disease-associated signatures or across cancer figures.


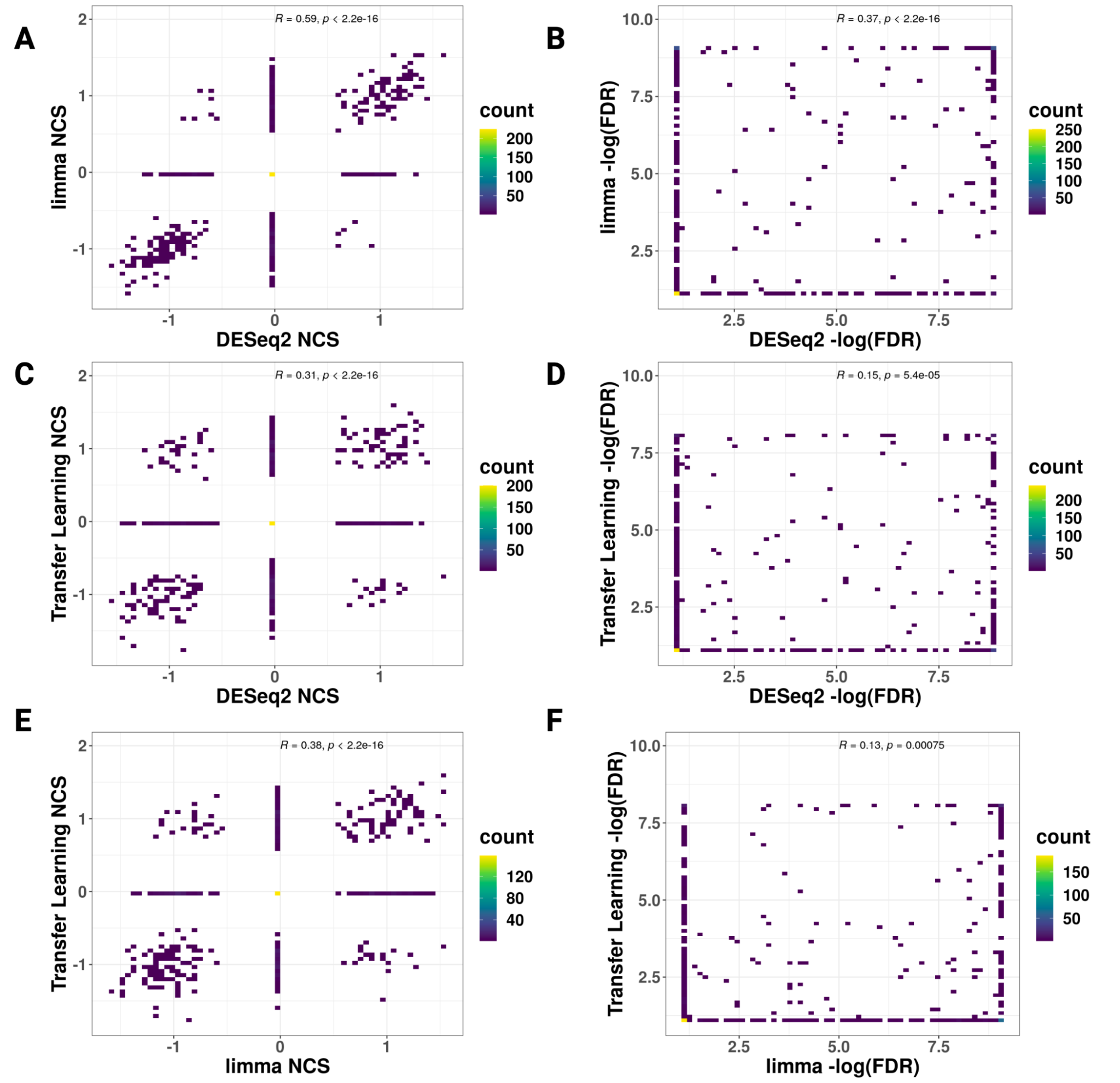


**Supplemental Figure 19: Signature reversion NCS and FDR scatter plots from each disease-associated gene signature for GBM. A)** DESeq2 vs limma disease-associated gene signature reversion NCS score. **B)** DESeq2 vs limma disease-associated gene signature reversion FDR **C)** DESeq2 vs transfer learning disease-associated gene signature reversion NCS score **D)** DESeq2 vs transfer learning disease-associated gene signature reversion FDR **E)** limma vs. transfer learning disease-associated gene signature reversion NCS score **F)** limma vs. transfer learning disease-associated gene signature reversion FDR. Spearman correlation and p-value from linear regression models are also plotted on each panel.


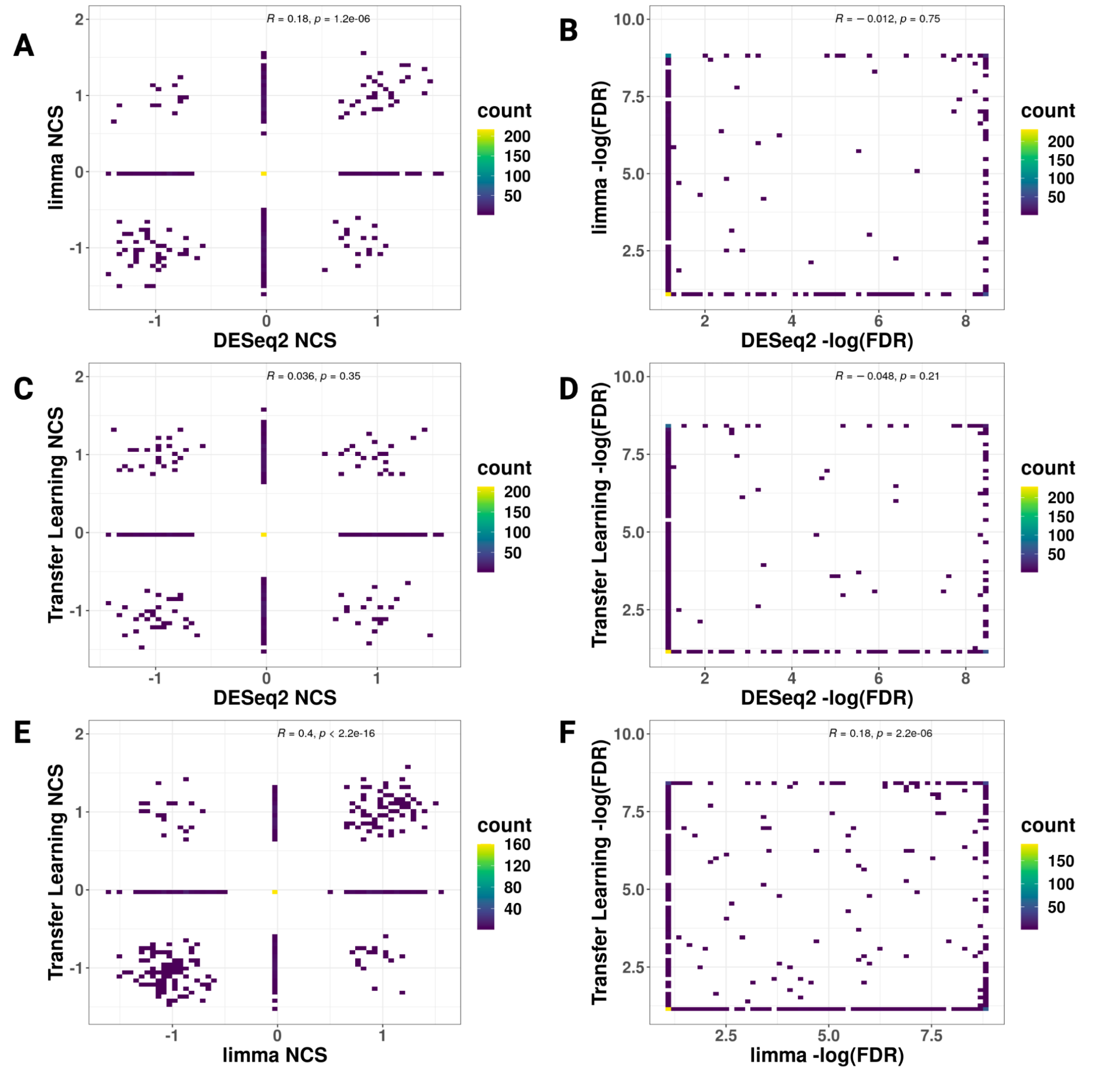


**Supplemental Figure 20: Signature reversion’s NCS and FDR scatter plots for all methods for LIHC. A)** DESeq2 vs limma disease-associated gene signature reversion NCS score. **B)** DESeq2 vs limma disease-associated gene signature reversion FDR **C)** DESeq2 vs transfer learning disease-associated gene signature reversion NCS score **D)** DESeq2 vs transfer learning disease-associated gene signature reversion FDR **E)** limma vs. transfer learning disease-associated gene signature reversion NCS score **F)** limma vs. transfer learning disease-associated gene signature reversion FDR. Spearman correlation and p-value from linear regression models are also plotted on each panel.


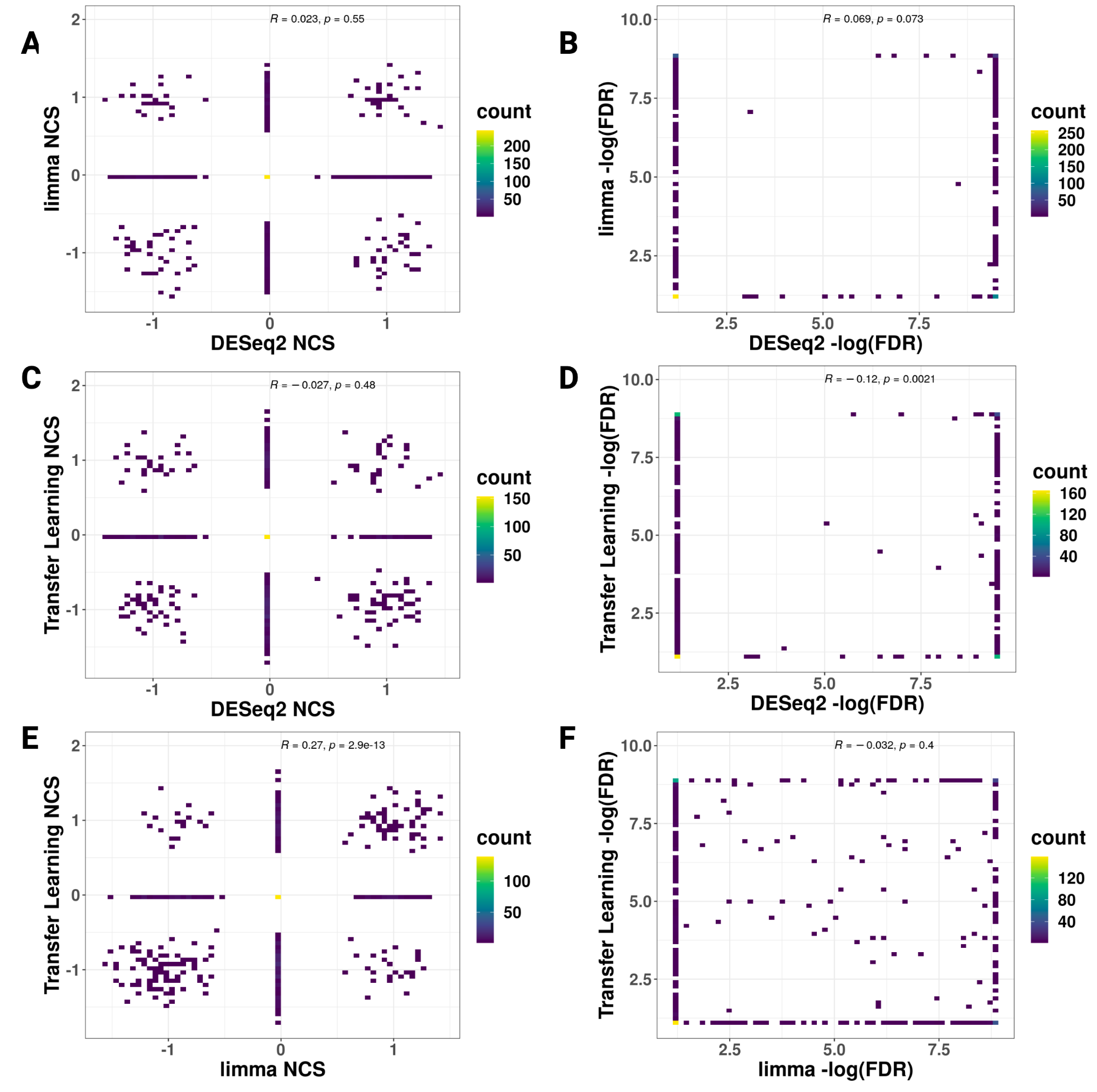


**Supplemental Figure 21: Signature reversion’s NCS and FDR scatter plots for all methods. A)** DESeq2 vs limma disease-associated gene signature reversion NCS score. **B)** DESeq2 vs limma disease-associated gene signature reversion FDR **C)** DESeq2 vs transfer learning disease-associated gene signature reversion NCS score **D)** DESeq2 vs transfer learning disease-associated gene signature reversion FDR **E)** limma vs. transfer learning disease-associated gene signature reversion NCS score **F)** limma vs. transfer learning disease-associated gene signature reversion FDR. Spearman correlation and p-value from linear regression models are also plotted on each panel.


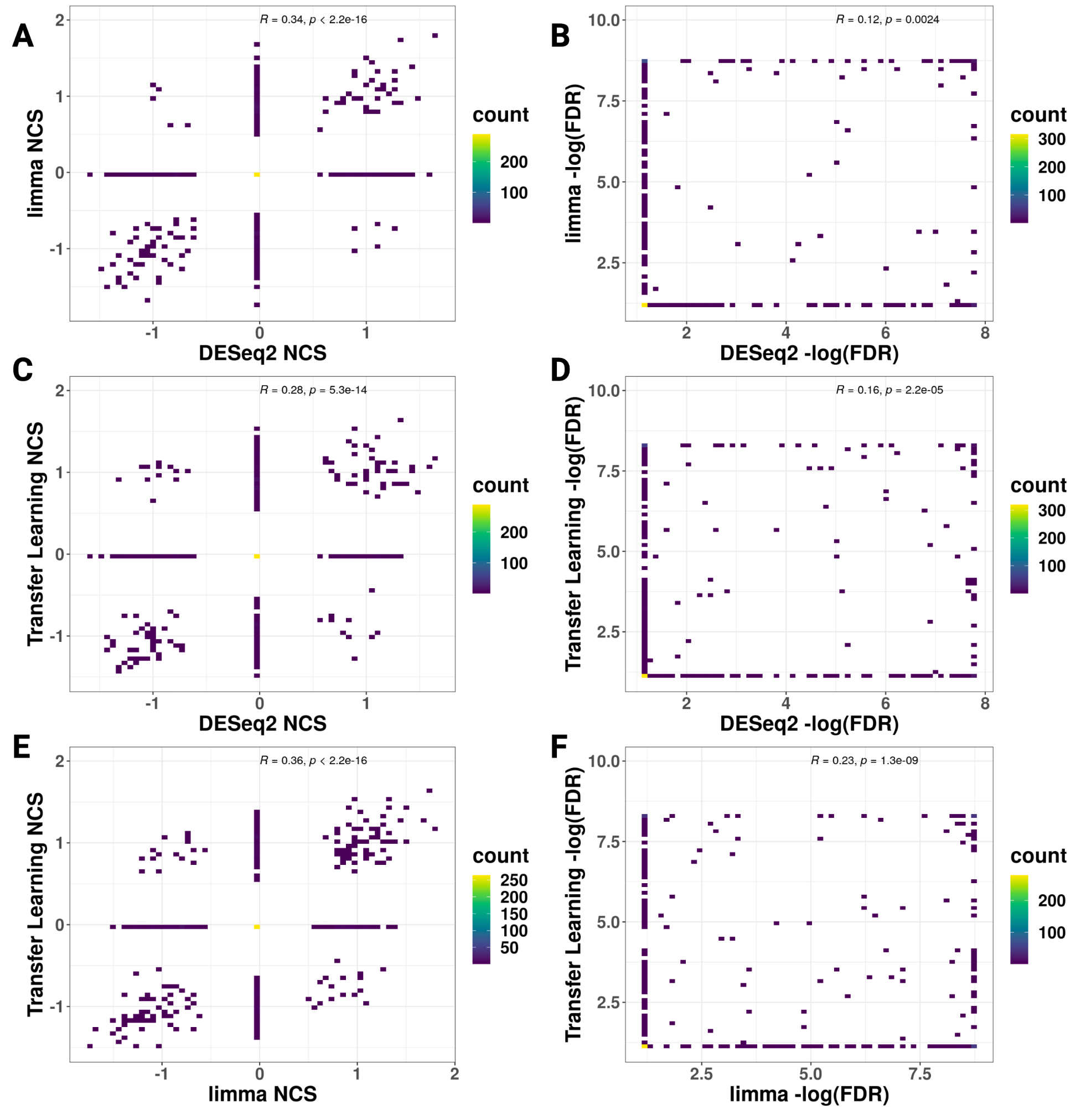


**Supplemental Figure 22: Signature reversion’s NCS and FDR scatter plots for all methods. A)** DESeq2 vs limma disease-associated gene signature reversion NCS score. **B)** DESeq2 vs limma disease-associated gene signature reversion FDR **C)** DESeq2 vs transfer learning disease-associated gene signature reversion NCS score **D)** DESeq2 vs transfer learning disease-associated gene signature reversion FDR **E)** limma vs. transfer learning disease-associated gene signature reversion NCS score **F)** limma vs. transfer learning disease-associated gene signature reversion FDR. Spearman correlation and p-value from linear regression models are also plotted on each panel.


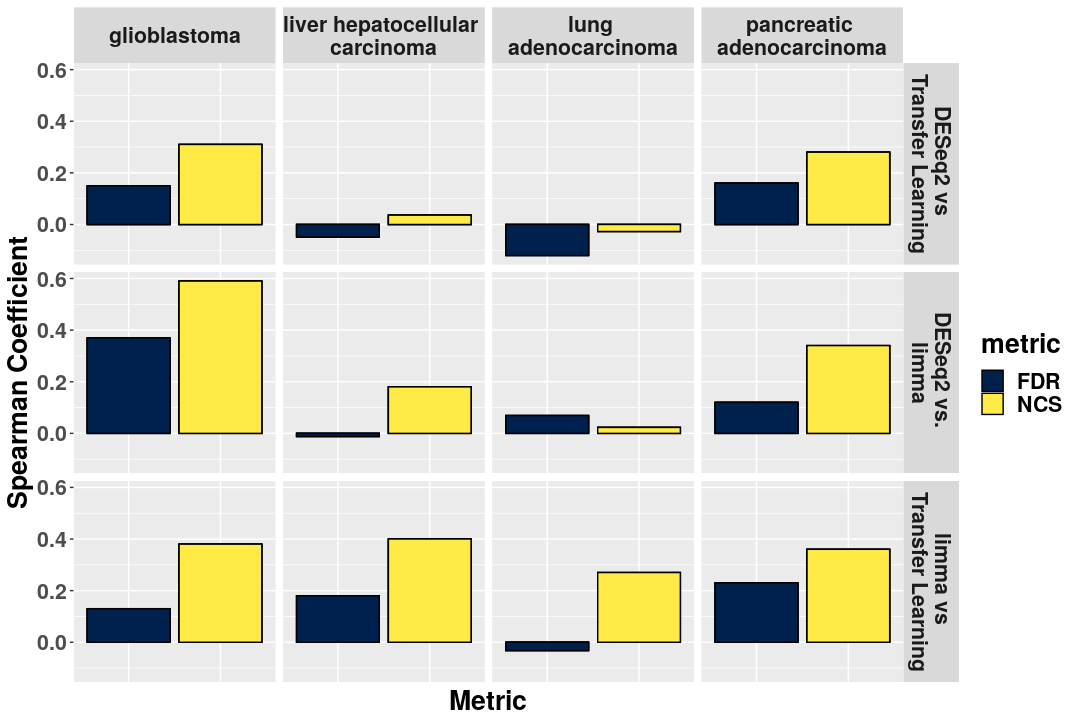


**Supplemental Figure 23:** A bar plot of the Spearman correlation of the normalized connectivity score (NCS) and the false discovery (FDR) between the different disease-associated gene signature reversion results across the different cancers.

**
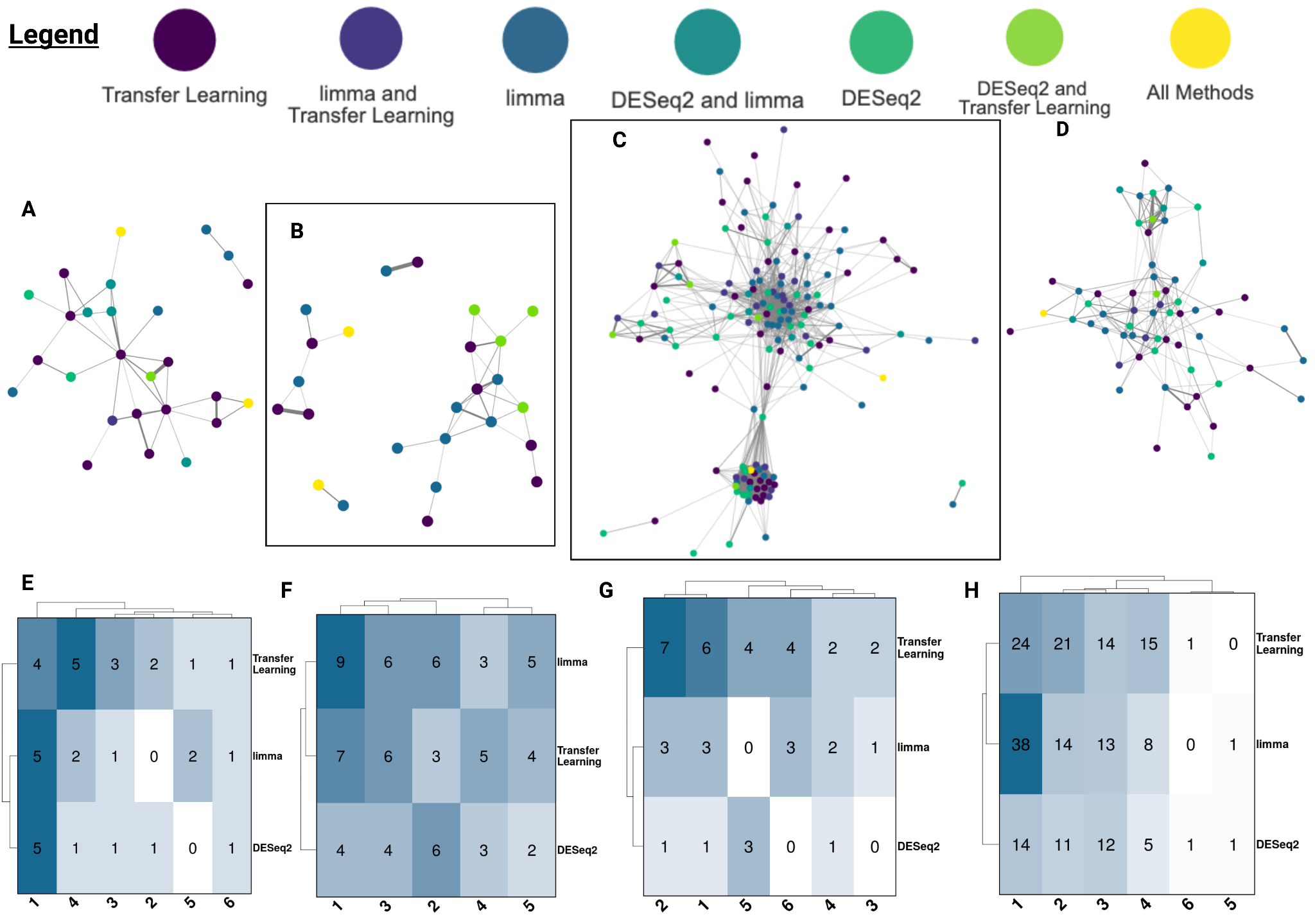
**

**Supplemental Figure 24: Drug-drug similarity networks - drug structure.** **A-D)** Drug-drug similarity networks based on the Tanimoto coefficient of SMILES drug structures where each node is a candidate colored by the disease-associated gene signature used to identify that candidate and the top 90% of edges displayed and weighted by cosine similarity for **A)** GBM candidates (GI1 profiles), **B)** LIHC cancer candidates (HEPG2 profiles), **C)** LUAD candidates (A529 LINCS profiles), and **D)** PAAD candidates (YPAC profiles). Heatmaps of the composition of the Leiden communities in the drug-drug similarity networks for **E)** GBM candidates (GI1 profiles), **F)** LIHC candidates (HEPG2 profiles), **G)** LUAD candidates (A529 LINCS profiles), and **H)** PAAD candidates (YPAC profiles).


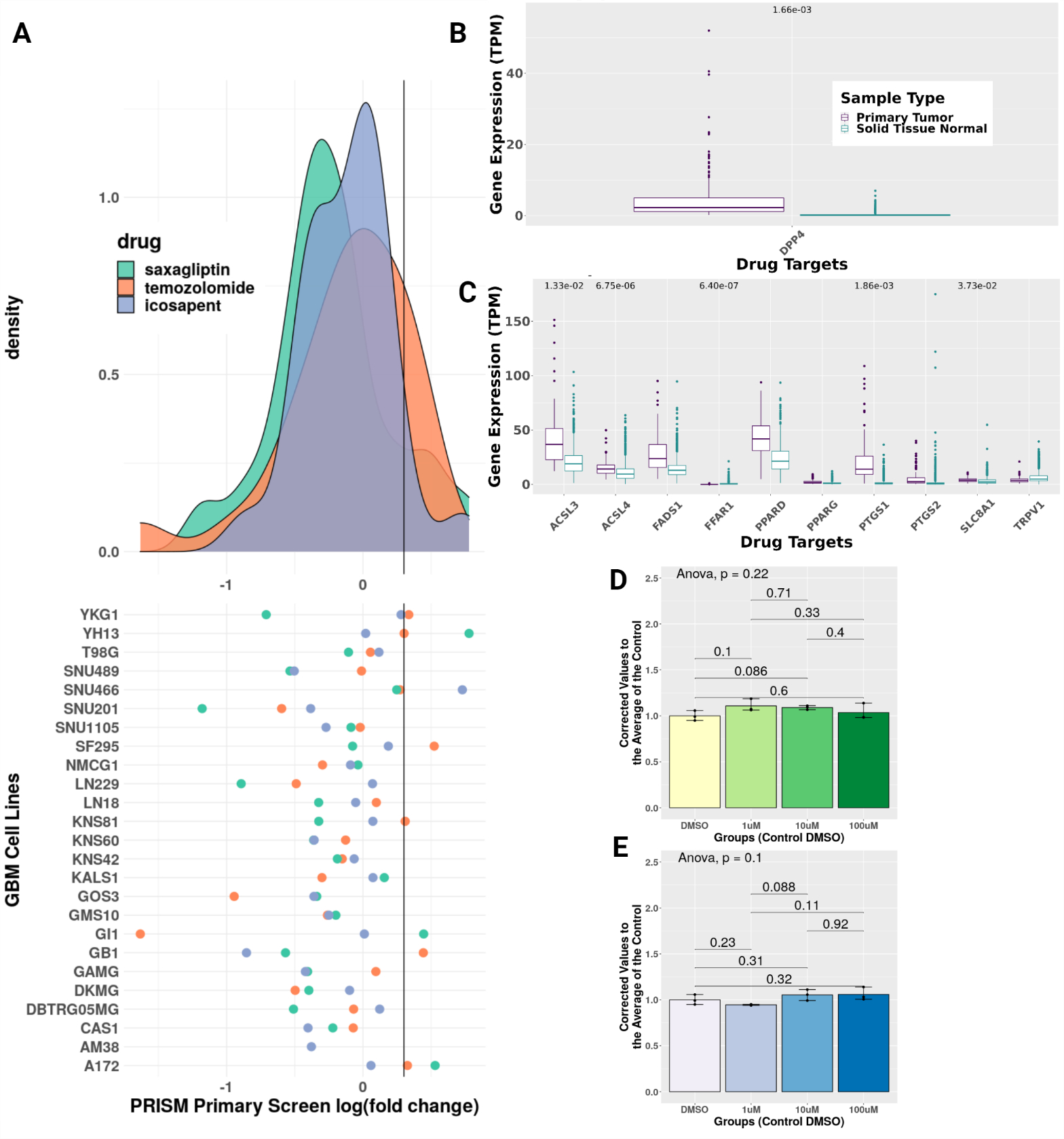

**Supplemental Figure 25: Screen for saxagliptin and icosapent growth inhibitory activity.** **A)** Density plot of candidates and TMZ log fold change from PRISM primary screen and dot plot of primary screen results for GBM cell lines. **B+C)** Boxplot of candidate’s drug target expression in tumor tissue and control brain tissue for saxagliptin **B** and icosapent **C**. **D)** CellTiter-Glo growth assay results for saxagliptin in U251 GBM cells. **E)** CellTiter-Glo growth assay results for icosapent in U251 GBM cells. ANOVA with Bonferroni corrected t-tests for pairwise comparisons with standard bars (n = 3).


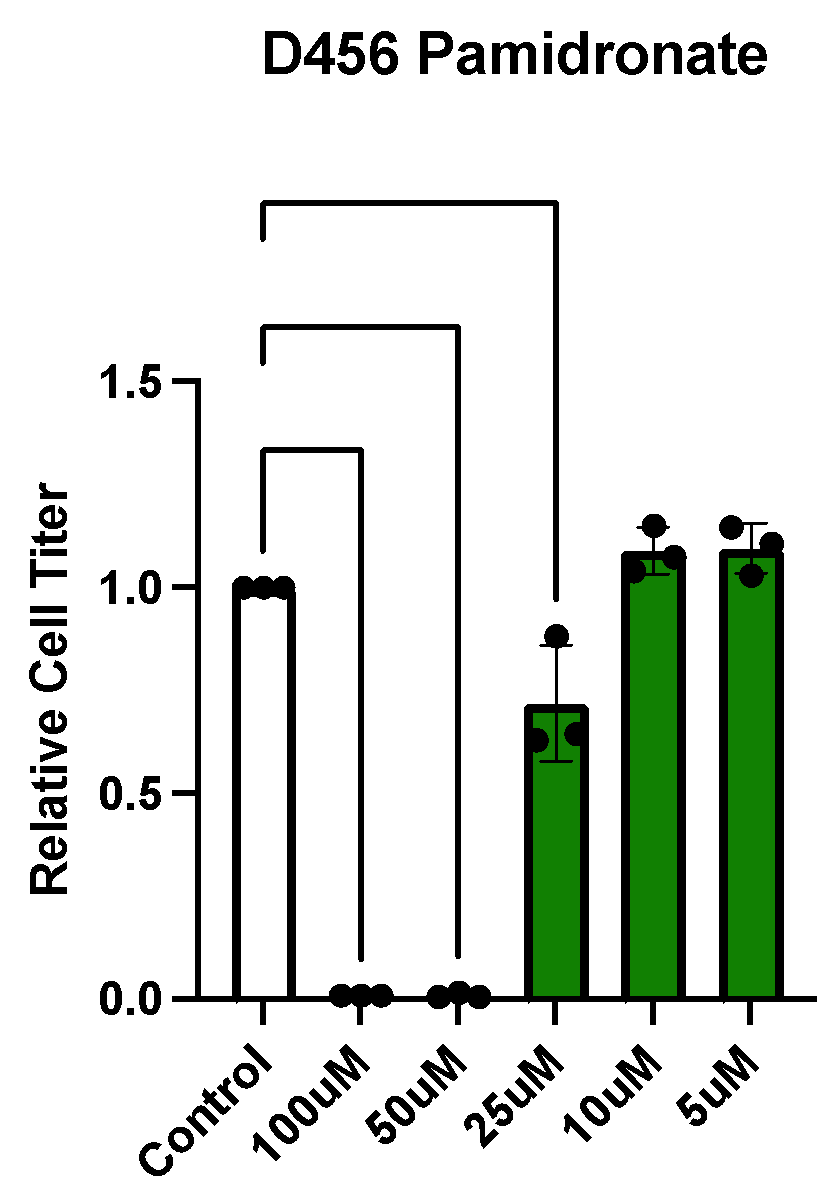



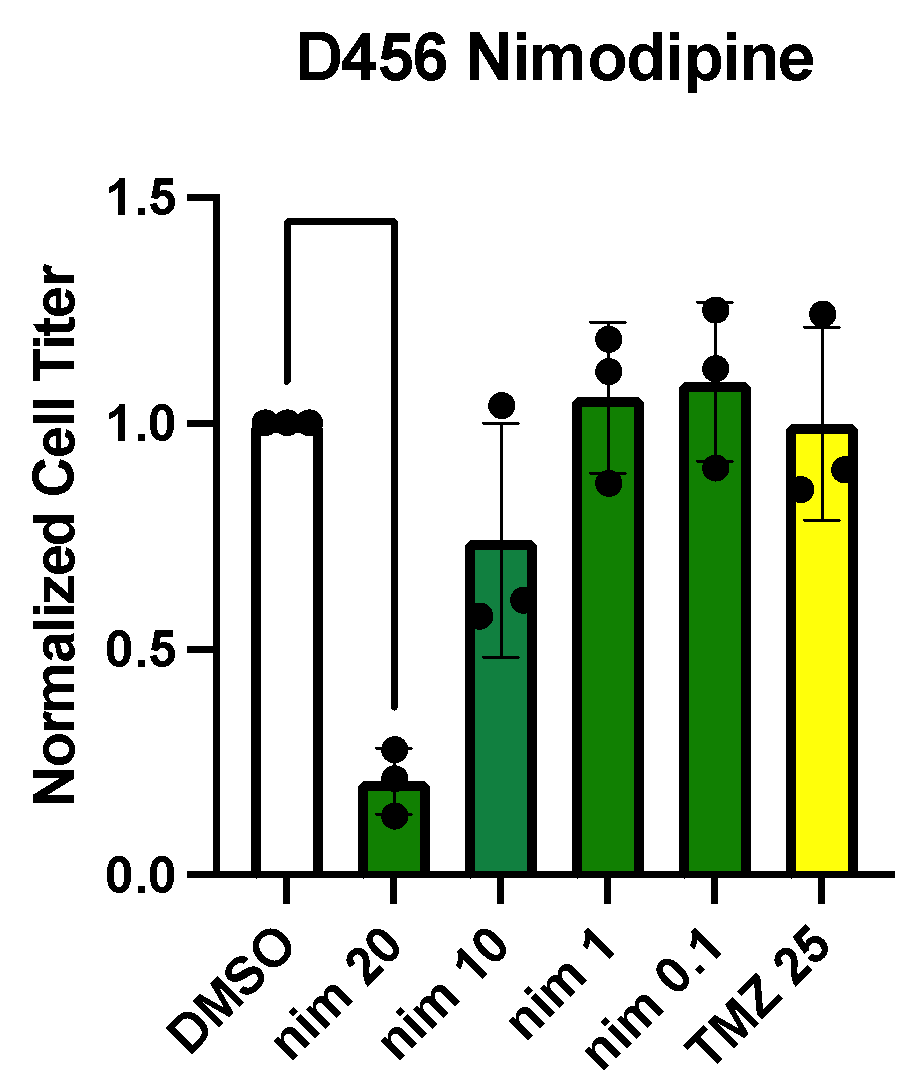


**Supplemental Figure 26: Pamidronate and nimodipine decrease the growth of D456 cells.** CellTiter-Glo viability assay results for pamidronate (A) or nimodipine (**B**) treatment of cells isolated from the GBM patient-derived xenograft D456. **P < 0.01, ***P < 0.001, ****P < 0.0001 ANOVA with comparison to vehicle control.



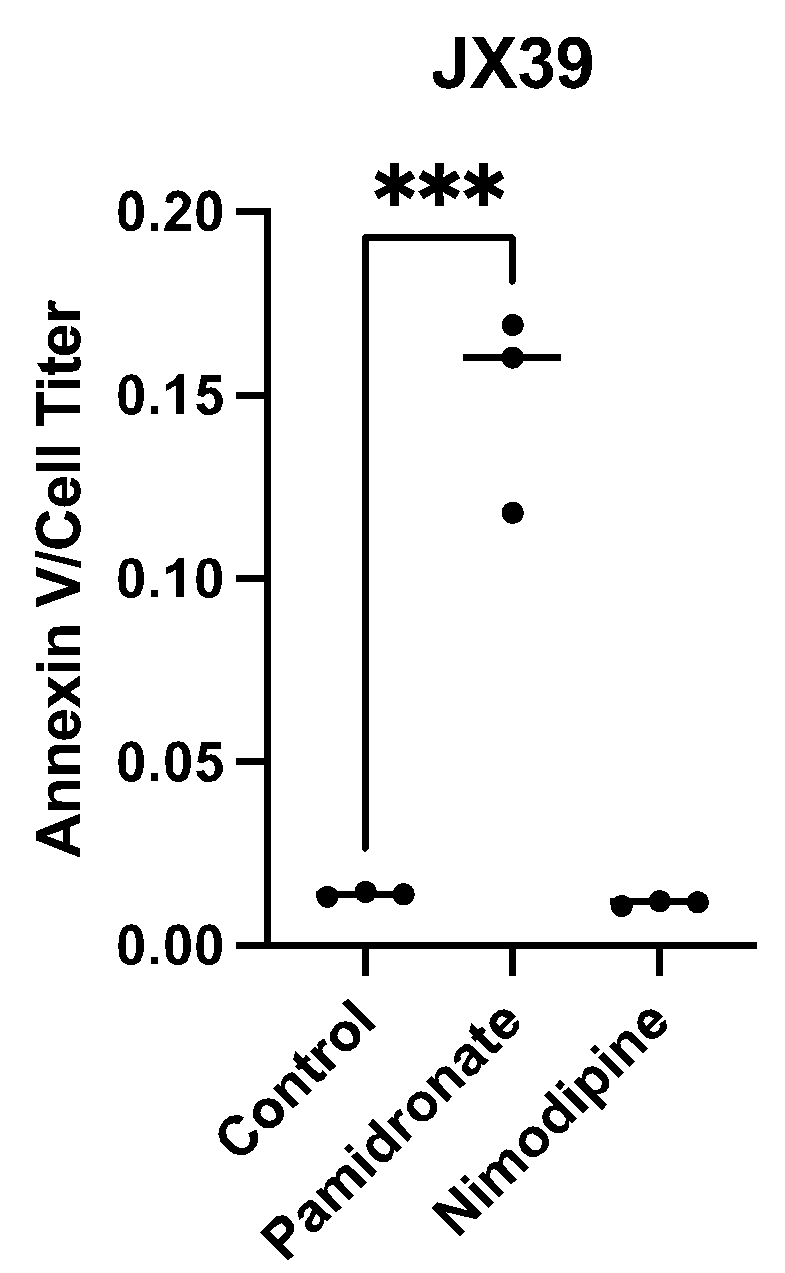


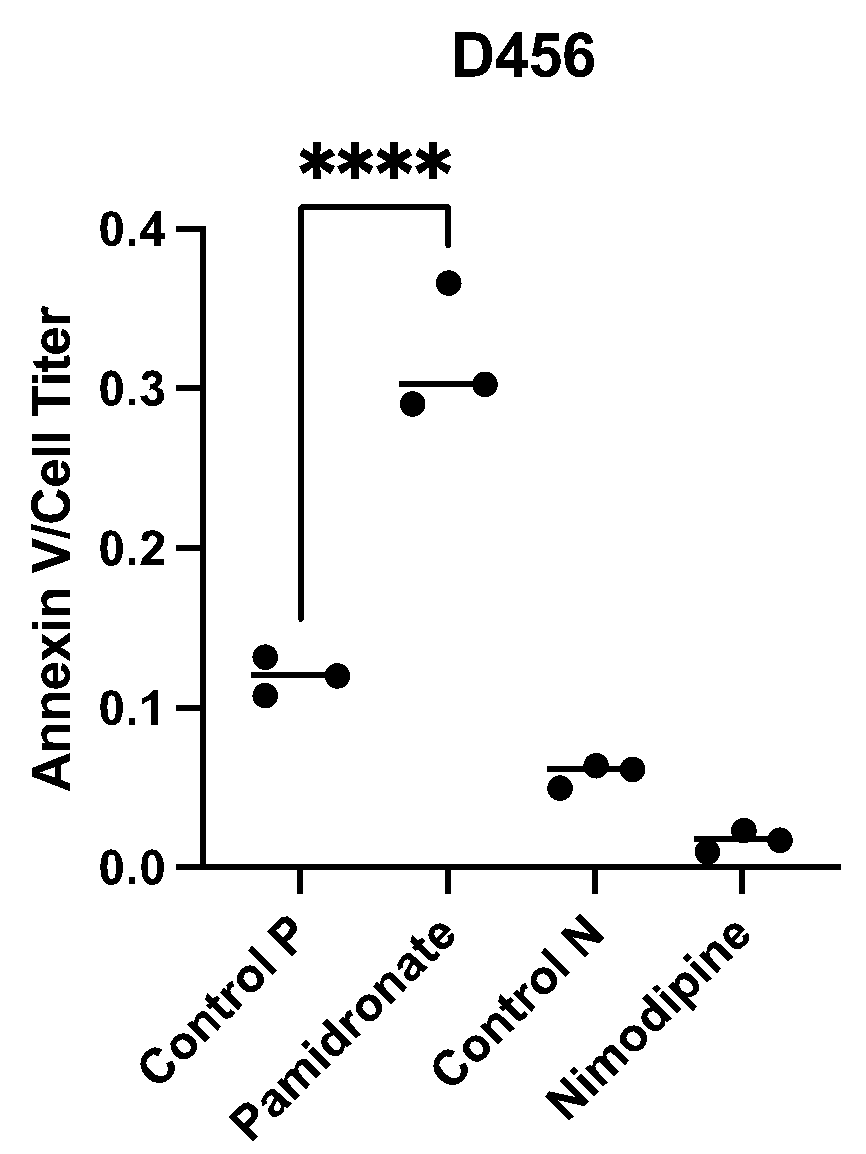


**Supplemental Figure 27: Pamidronate, but not Nimodipine, induces Apoptosis.** Results from the RealTime-Glo Annexin V Apoptosis and Necrosis Assay normalized to Cell Titer Glo indicate increases in Annexin V mediated luminescence with 48 hours pamidronate (50µM) treatment of 10,000 cells isolated from **A)** JX39 and **B)** D456 GBM patient derived xenografts. There is no increase in Annexin V with treatment for 48 hours with 50µM nimodipine in JX39 cells or 25µM in D456 cells. Results shown are biological triplicates of the average of duplicate RealTime-Glo luminescent values divided by Cell Titer glo values collected at the same time point from identically treated wells on the same plate. ***, p<0.001; ****, p<0.0001 with ANOVA or t-test comparison to the relevant control.


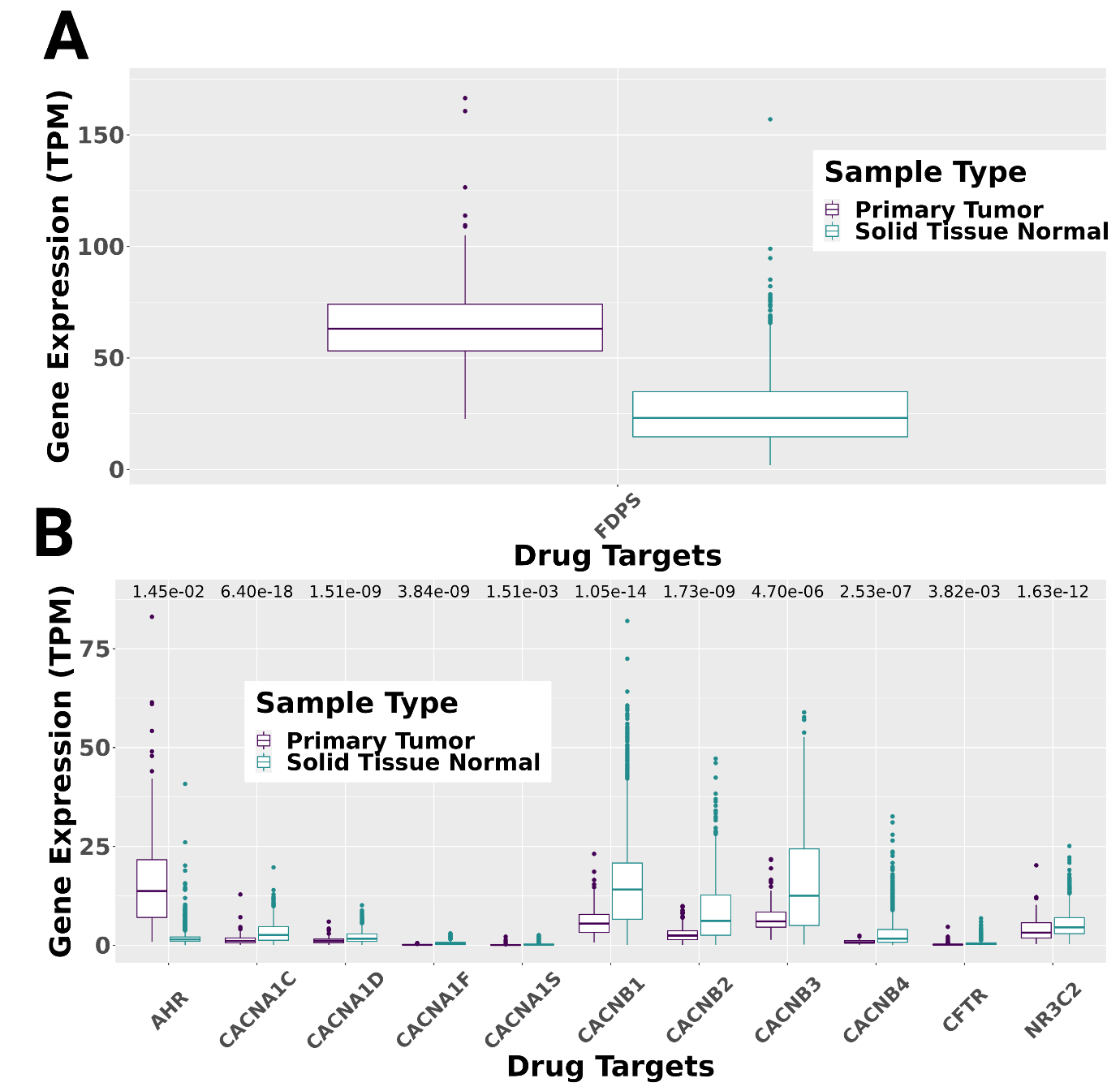


**Supplemental Figure 28: Gene Expression of pamidronate and nimodipine drug targets in primary tumor and solid tissue normal samples.** Boxplot of drug target expression in tumor tissue and control brain tissue for **A)** pamidronate (adjusted p-values = 0.1189) and **B)** nimodipine, respectively. Significant adjusted p-values from DESeq2 are plotted above the drug target expression.


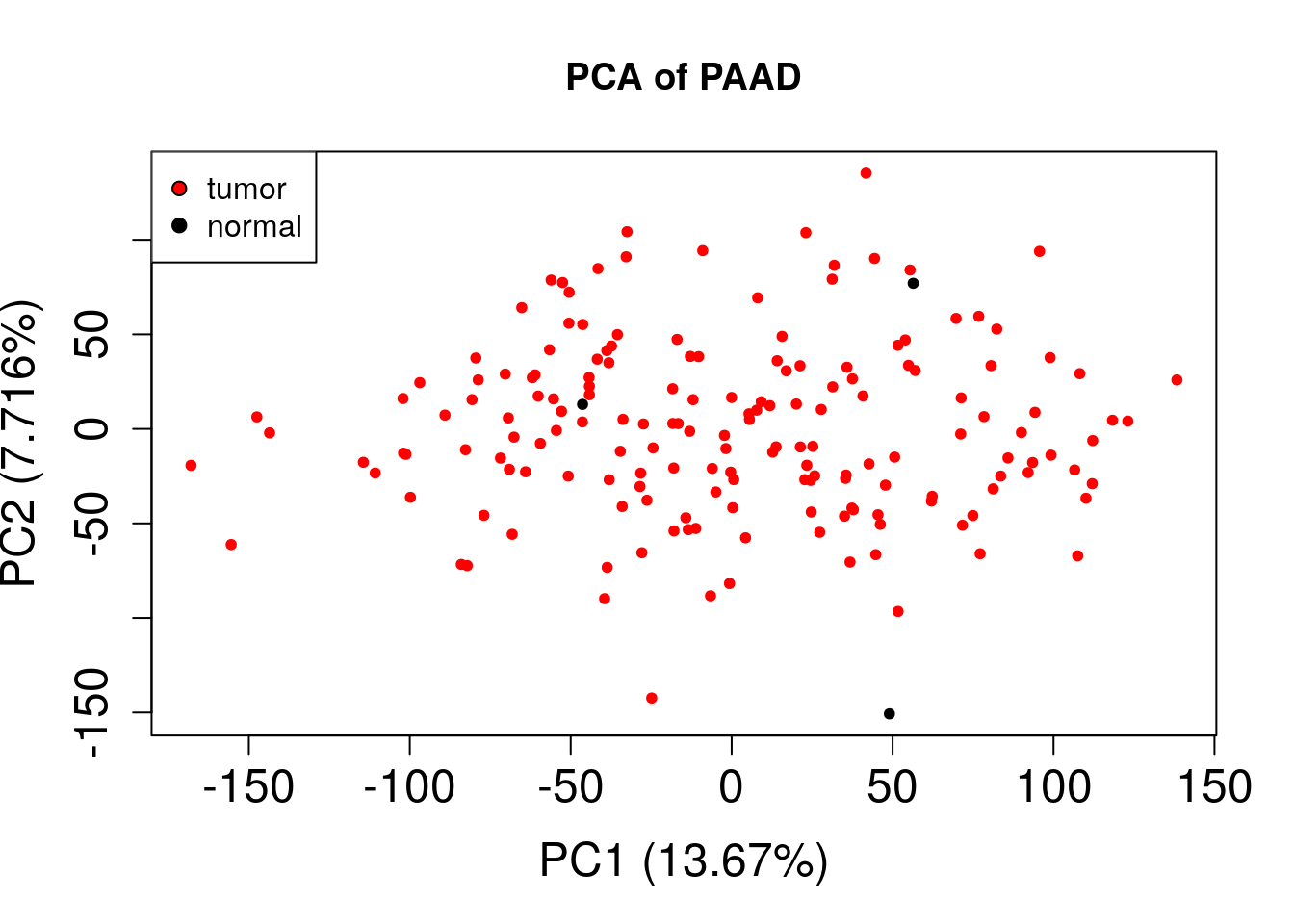


**Supplemental Figure 29: Principal component analysis for PAAD TCGA samples.** Scatterplot of PC1 (x-axis) and PC2 (y-aixs).

**
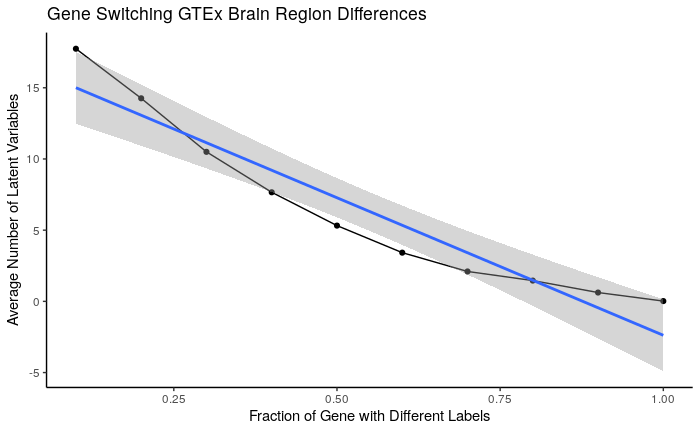
**

**Supplemental Figure 30: Gene label switching test for transfer learning approach.** GTEx brain frontal cortex and cerebellar hemisphere regions were compared with the transfer learning approach by determining latent variables that were different between these two regions. A fraction of the genes had their labels switched with another gene. This was done in 10% intervals starting at 10% and ending at 100% of all the genes. This process was done 50 times at each interval. The average number of latent variables significant for each interval was calculated (adj. p-value < 0.05; fold change of 0.05). A linear regression model was used to determine if there was an influence of the gene label and the number of latent variables (R^2^ = 0.9066 & p-value = 1.346e-05).
