# Supplementary material for: Signature reversion of three disease‐associated gene signatures prioritizes cancer drug repurposing candidates
Source: FEBS Open Bio. 2024 Mar 26;14(5):803–30. doi: 10.1002/2211-5463.13796 (PMC11073506; doi:10.1002/2211-5463.13796)
Supplement: Supplementary file 5 — Data S4. Computing system and package version information. [file FEB4-14-803-s003.pdf]

Supplemental File 4: Detailed computer and package version information.

221221_rstudio_tf_dr_v3_singularity

Jennifer Fisher

12/21/2022

```
GitHub Repo:
Transfer_Learning_R03

Docker:
rstudio_tf_dr_v3

Directory of operations:
/data/project/lasseigne_lab/JLF_scratch/Transfer_Learning_R03 (singularity)
```

```
sessionInfo()
```

```
## R version 4.1.2 (2021-11-01)
## Platform: x86_64-pc-linux-gnu (64-bit)
## Running under: Ubuntu 20.04.3 LTS
##
## Matrix products: default
## BLAS/LAPACK: /usr/lib/x86_64-linux-gnu/openblas-pthread/libopenblasp-r0.3.8.so
##
## locale:
##  [1] LC_CTYPE=en_US.UTF-8       LC_NUMERIC=C
##  [3] LC_TIME=en_US.UTF-8        LC_COLLATE=en_US.UTF-8
##  [5] LC_MONETARY=en_US.UTF-8    LC_MESSAGES=C
##  [7] LC_PAPER=en_US.UTF-8       LC_NAME=C
##  [9] LC_ADDRESS=C               LC_TELEPHONE=C
## [11] LC_MEASUREMENT=en_US.UTF-8 LC_IDENTIFICATION=C
##
## attached base packages:
## [1] stats     graphics  grDevices utils     datasets  methods   base
##
## loaded via a namespace (and not attached):
##  [1] digest_0.6.29   R6_2.5.1        jsonlite_1.7.2  magrittr_2.0.1
##  [5] evaluate_0.14   rlang_0.4.12    stringi_1.7.6   jquerylib_0.1.4
##  [9] bslib_0.3.1     rmarkdown_2.11  tools_4.1.2     stringr_1.4.0
## [13] xfun_0.28       yaml_2.2.1      fastmap_1.1.0   compiler_4.1.2
## [17] htmltools_0.5.2 knitr_1.36      sass_0.4.0
```

```
# Listing packages
installed.packages()[,c(1,3)]
```

```
##                       Package             Version
## abind                 "abind"             "1.4-5"
## affy                  "affy"              "1.72.0"
## affyio                "affyio"            "1.64.0"
## amap                  "amap"              "0.8-18"
## annotate              "annotate"          "1.72.0"
## AnnotationDbi         "AnnotationDbi"     "1.56.2"
## AnnotationFilter      "AnnotationFilter"  "1.18.0"
## AnnotationHub         "AnnotationHub"     "3.0.2"
## AnVIL                 "AnVIL"             "1.6.0"
## apcluster             "apcluster"         "1.4.8"
## ape                   "ape"               "5.5"
## aplot                 "aplot"             "0.1.1"
## askpass               "askpass"           "1.1"
## assertthat            "assertthat"        "0.2.1"
## backports             "backports"         "1.4.0"
## base64enc             "base64enc"         "0.1-3"
## BgeeDB                "BgeeDB"            "2.20.0"
## BH                    "BH"                "1.75.0-0"
## Biobase               "Biobase"           "2.54.0"
## BiocFileCache         "BiocFileCache"     "2.2.0"
## BiocGenerics          "BiocGenerics"      "0.40.0"
## BiocIO                "BiocIO"            "1.4.0"
## BiocManager           "BiocManager"       "1.30.16"
## BiocParallel          "BiocParallel"      "1.28.2"
## BiocVersion           "BiocVersion"       "3.14.0"
## biomaRt               "biomaRt"           "2.50.1"
## Biostrings            "Biostrings"        "2.62.0"
## bit                   "bit"               "4.0.4"
## bit64                 "bit64"             "4.0.5"
## bitops                "bitops"            "1.0-7"
## biwt                  "biwt"              "1.0"
## blob                  "blob"              "1.2.2"
## brew                  "brew"              "1.0-6"
## brio                  "brio"              "1.1.2"
## broom                 "broom"             "0.7.10"
## bslib                 "bslib"             "0.3.1"
## cachem                "cachem"            "1.0.6"
## callr                 "callr"             "3.7.0"
## car                   "car"               "3.0-12"
## carData               "carData"           "3.0-4"
## caret                 "caret"             "6.0-90"
## caTools               "caTools"           "1.18.2"
## cellranger            "cellranger"        "1.1.0"
## circlize              "circlize"          "0.4.13"
## cli                   "cli"               "3.1.0"
## clipr                 "clipr"             "0.7.1"
## clue                  "clue"              "0.3-60"
## clusterProfiler       "clusterProfiler"   "4.0.5"
## CoGAPS                "CoGAPS"            "3.14.0"
## cogena                "cogena"            "1.28.0"
## colorspace            "colorspace"        "2.0-2"
```

```
## commonmark           "commonmark"           "1.7"
## ComplexHeatmap       "ComplexHeatmap"       "2.10.0"
## ComplexUpset         "ComplexUpset"         "1.3.1"
## conquer              "conquer"              "1.2.1"
## corrplot             "corrplot"             "0.92"
## cowplot              "cowplot"              "1.1.1"
## cpp11                "cpp11"                "0.4.2"
## crayon               "crayon"               "1.4.2"
## credentials          "credentials"          "1.3.1"
## crosstalk            "crosstalk"            "1.2.0"
## curl                 "curl"                 "4.3.2"
## data.table           "data.table"           "1.14.2"
## DBI                  "DBI"                  "1.1.1"
## dbplyr               "dbplyr"               "1.3.0"
## DelayedArray         "DelayedArray"         "0.20.0"
## dendextend           "dendextend"           "1.15.2"
## DEoptimR             "DEoptimR"             "1.0-9"
## desc                 "desc"                 "1.4.0"
## DESeq2               "DESeq2"               "1.34.0"
## devtools             "devtools"             "2.4.3"
## diffobj              "diffobj"              "0.3.5"
## digest               "digest"               "0.6.29"
## DO.db                "DO.db"                "2.9"
## docopt               "docopt"               "0.7.1"
## doParallel           "doParallel"           "1.0.16"
## DOSE                 "DOSE"                 "3.18.3"
## downloader           "downloader"           "0.4"
## dplyr                "dplyr"                "1.0.7"
## DT                   "DT"                   "0.20"
## dtplyr               "dtplyr"               "1.2.0"
## e1071                "e1071"                "1.7-9"
## ellipse              "ellipse"              "0.4.2"
## ellipsis             "ellipsis"             "0.3.2"
## enrichplot           "enrichplot"           "1.12.3"
## EnsDb.Hsapiens.v75   "EnsDb.Hsapiens.v75"   "2.99.0"
## ensembldb            "ensembldb"            "2.18.2"
## evaluate             "evaluate"             "0.14"
## ExperimentHub        "ExperimentHub"        "2.0.0"
## factoextra           "factoextra"           "1.0.7"
## FactoMineR           "FactoMineR"           "2.4"
## fansi                "fansi"                "0.5.0"
## farver               "farver"               "2.1.0"
## fastcluster          "fastcluster"          "1.2.3"
## fastmap              "fastmap"              "1.1.0"
## fastmatch            "fastmatch"            "1.1-3"
## fgsea                "fgsea"                "1.18.0"
## filelock             "filelock"             "1.0.2"
## flashClust           "flashClust"           "1.01-2"
## fontawesome          "fontawesome"          "0.2.2"
## forcats              "forcats"              "0.5.1"
## foreach              "foreach"              "1.5.1"
## formatR              "formatR"              "1.11"
```

```
## fs                    "fs"                    "1.5.2"
## futile.logger         "futile.logger"         "1.4.3"
## futile.options        "futile.options"        "1.0.1"
## future                "future"                "1.23.0"
## future.apply          "future.apply"          "1.8.1"
## gargle                "gargle"                "1.2.0"
## genefilter            "genefilter"            "1.76.0"
## geneplotter           "geneplotter"           "1.72.0"
## generics              "generics"              "0.1.1"
## GenomeInfoDb          "GenomeInfoDb"          "1.30.0"
## GenomeInfoDbData      "GenomeInfoDbData"      "1.2.7"
## GenomicAlignments     "GenomicAlignments"     "1.30.0"
## GenomicFeatures       "GenomicFeatures"       "1.46.1"
## GenomicRanges         "GenomicRanges"         "1.46.1"
## GEOquery              "GEOquery"              "2.62.1"
## gert                  "gert"                  "1.4.3"
## GetoptLong            "GetoptLong"            "1.0.5"
## ggalluvial            "ggalluvial"            "0.12.3"
## ggforce               "ggforce"               "0.3.3"
## ggfun                 "ggfun"                 "0.0.4"
## ggplot2               "ggplot2"               "3.3.5"
## ggplotify             "ggplotify"             "0.1.0"
## ggpubr                "ggpubr"                "0.4.0"
## ggraph                "ggraph"                "2.0.5"
## ggrepel               "ggrepel"               "0.9.1"
## ggsci                 "ggsci"                 "2.9"
## ggsignif              "ggsignif"              "0.6.3"
## ggtree                "ggtree"                "3.0.4"
## gh                    "gh"                    "1.3.0"
## gitcreds              "gitcreds"              "0.1.1"
## glmnet                "glmnet"                "4.1-3"
## GlobalOptions         "GlobalOptions"         "0.1.2"
## globals               "globals"               "0.14.0"
## glue                  "glue"                  "1.5.1"
## GO.db                 "GO.db"                 "3.14.0"
## googledrive           "googledrive"           "2.0.0"
## googlesheets4         "googlesheets4"         "1.0.0"
## GOSemSim              "GOSemSim"              "2.18.1"
## gower                 "gower"                 "0.2.2"
## gplots                "gplots"                "3.1.1"
## gprofiler2            "gprofiler2"            "0.2.1"
## graph                 "graph"                 "1.72.0"
## graphlayouts          "graphlayouts"          "0.7.2"
## gridBase              "gridBase"              "0.4-7"
## gridExtra             "gridExtra"             "2.3"
## gridGraphics          "gridGraphics"          "0.5-1"
## GSEABase              "GSEABase"              "1.54.0"
## gtable                "gtable"                "0.3.0"
## gtools                "gtools"                "3.9.2"
## haven                 "haven"                 "2.4.3"
## HDF5Array             "HDF5Array"             "1.20.0"
## highr                 "highr"                 "0.9"
```

```
## hms                    "hms"                    "1.1.1"
## htmltools              "htmltools"              "0.5.2"
## htmlwidgets            "htmlwidgets"            "1.5.4"
## httpuv                 "httpuv"                 "1.6.3"
## httr                   "httr"                   "1.4.2"
## ids                    "ids"                    "1.0.1"
## igraph                 "igraph"                 "1.2.9"
## ini                    "ini"                    "0.3.1"
## interactiveDisplayBase "interactiveDisplayBase" "1.30.0"
## ipred                  "ipred"                  "0.9-12"
## IRanges                "IRanges"                "2.28.0"
## isoband                "isoband"                "0.2.5"
## iterators              "iterators"              "1.0.13"
## jquerylib              "jquerylib"              "0.1.4"
## jsonlite               "jsonlite"               "1.7.2"
## KEGGREST               "KEGGREST"               "1.34.0"
## knitr                  "knitr"                  "1.36"
## kohonen                "kohonen"                "3.0.10"
## labeling               "labeling"               "0.4.2"
## lambda.r               "lambda.r"               "1.2.4"
## later                  "later"                  "1.3.0"
## lava                   "lava"                   "1.6.10"
## lazyeval               "lazyeval"               "0.2.2"
## leaps                  "leaps"                  "3.1"
## lifecycle              "lifecycle"              "1.0.1"
## limma                  "limma"                  "3.50.0"
## listenv                "listenv"                "0.8.0"
## littler                "littler"                "0.3.14"
## lme4                   "lme4"                   "1.1-27.1"
## locfit                 "locfit"                 "1.5-9.4"
## lubridate              "lubridate"              "1.8.0"
## magrittr               "magrittr"               "2.0.1"
## maptools               "maptools"               "1.1-2"
## markdown               "markdown"               "1.1"
## MatrixGenerics         "MatrixGenerics"         "1.6.0"
## MatrixModels           "MatrixModels"           "0.5-0"
## matrixStats            "matrixStats"            "0.61.0"
## mclust                 "mclust"                 "5.4.8"
## memoise                "memoise"                "2.0.1"
## mime                   "mime"                   "0.12"
## minqa                  "minqa"                  "1.2.4"
## ModelMetrics           "ModelMetrics"           "1.2.2.2"
## modelr                 "modelr"                 "0.1.8"
## munsell                "munsell"                "0.5.0"
## mvtnorm                "mvtnorm"                "1.1-3"
## nloptr                 "nloptr"                 "1.2.2.3"
## NMF                    "NMF"                    "0.23.0"
## numDeriv               "numDeriv"               "2016.8-1.1"
## openssl                "openssl"                "1.4.5"
## org.Hs.eg.db           "org.Hs.eg.db"           "3.14.0"
## parallelly             "parallelly"             "1.29.0"
## pasilla                "pasilla"                "1.22.0"
```

```
## patchwork            "patchwork"            "1.1.1"
## pbkrtest             "pbkrtest"             "0.5.1"
## pcaPP                "pcaPP"                "1.9-74"
## pheatmap             "pheatmap"             "1.0.12"
## pillar               "pillar"               "1.6.4"
## pkgbuild             "pkgbuild"             "1.2.0"
## pkgconfig            "pkgconfig"            "2.0.3"
## pkgload              "pkgload"              "1.2.3"
## pkgmaker             "pkgmaker"             "0.32.2"
## PLIER                "PLIER"                "0.99.0"
## plogr                "plogr"                "0.2.0"
## plotly               "plotly"               "4.10.0"
## plyr                 "plyr"                 "1.8.6"
## png                  "png"                  "0.1-7"
## polyclip             "polyclip"             "1.10-0"
## polynom              "polynom"              "1.4-0"
## praise               "praise"               "1.0.0"
## preprocessCore       "preprocessCore"       "1.56.0"
## prettyunits          "prettyunits"          "1.1.1"
## pROC                 "pROC"                 "1.18.0"
## processx             "processx"             "3.5.2"
## prodlim              "prodlim"              "2019.11.13"
## progress             "progress"             "1.2.2"
## progressr            "progressr"            "0.9.0"
## projectR             "projectR"             "1.10.0"
## ProliferativeIndex   "ProliferativeIndex"   "1.0.1"
## promises             "promises"             "1.2.0.1"
## ProtGenerics         "ProtGenerics"         "1.26.0"
## proxy                "proxy"                "0.4-26"
## ps                   "ps"                   "1.6.0"
## purrr                "purrr"                "0.3.4"
## quantreg             "quantreg"             "5.86"
## qvalue               "qvalue"               "2.24.0"
## R.methodsS3          "R.methodsS3"          "1.8.1"
## R.oo                 "R.oo"                 "1.24.0"
## R.utils              "R.utils"              "2.11.0"
## R6                   "R6"                   "2.5.1"
## rapiclient           "rapiclient"           "0.1.3"
## rappdirs             "rappdirs"             "0.3.3"
## rcmdcheck            "rcmdcheck"            "1.4.0"
## RColorBrewer         "RColorBrewer"         "1.1-2"
## Rcpp                 "Rcpp"                 "1.0.7"
## RcppArmadillo        "RcppArmadillo"        "0.10.7.3.0"
## RcppEigen            "RcppEigen"            "0.3.3.9.1"
## RCurl                "RCurl"                "1.98-1.5"
## reactome.db          "reactome.db"          "1.76.0"
## readr                "readr"                "2.1.1"
## readxl               "readxl"               "1.3.1"
## recipes              "recipes"              "0.1.17"
## recount3             "recount3"             "1.4.0"
## registry             "registry"             "0.5-1"
## rematch              "rematch"              "1.0.1"
```

```
## rematch2              "rematch2"              "2.1.2"
## remotes               "remotes"               "2.4.1"
## reprex                "reprex"                "2.0.1"
## reshape2              "reshape2"              "1.4.4"
## restfulr              "restfulr"              "0.0.13"
## rhdf5                 "rhdf5"                 "2.38.0"
## rhdf5filters          "rhdf5filters"          "1.6.0"
## Rhdf5lib              "Rhdf5lib"              "1.16.0"
## Rhtslib               "Rhtslib"               "1.26.0"
## rjson                 "rjson"                 "0.2.20"
## rlang                 "rlang"                 "0.4.12"
## rmarkdown             "rmarkdown"             "2.11"
## rngtools              "rngtools"              "1.5.2"
## robustbase            "robustbase"            "0.93-9"
## ROCR                  "ROCR"                  "1.0-11"
## roxygen2              "roxygen2"              "7.1.2"
## rprojroot             "rprojroot"             "2.0.2"
## rrcov                 "rrcov"                 "1.6-0"
## Rsamtools             "Rsamtools"             "2.10.0"
## RSQLite               "RSQLite"               "2.2.9"
## rstatix               "rstatix"               "0.7.0"
## rstudioapi            "rstudioapi"            "0.13"
## rsvd                  "rsvd"                  "1.0.5"
## rtracklayer           "rtracklayer"           "1.54.0"
## rversions             "rversions"             "2.1.1"
## rvest                 "rvest"                 "1.0.2"
## S4Vectors             "S4Vectors"             "0.32.3"
## sass                  "sass"                  "0.4.0"
## scales                "scales"                "1.1.1"
## scatterpie            "scatterpie"            "0.1.7"
## scatterplot3d         "scatterplot3d"         "0.3-41"
## selectr               "selectr"               "0.4-2"
## sessioninfo           "sessioninfo"           "1.2.1"
## shadowtext            "shadowtext"            "0.0.9"
## shape                 "shape"                 "1.4.6"
## shiny                 "shiny"                 "1.7.1"
## signatureSearch       "signatureSearch"       "1.9.2"
## signatureSearchData   "signatureSearchData"   "1.8.2"
## SingleCellExperiment  "SingleCellExperiment"  "1.16.0"
## snow                  "snow"                  "0.4-4"
## sourcetools           "sourcetools"           "0.1.7"
## sp                    "sp"                    "1.4-6"
## SparseM               "SparseM"               "1.81"
## SQUAREM               "SQUAREM"               "2021.1"
## stringi               "stringi"               "1.7.6"
## stringr               "stringr"               "1.4.0"
## SummarizedExperiment  "SummarizedExperiment"  "1.24.0"
## sys                   "sys"                   "3.4"
## testthat              "testthat"              "3.1.0"
## TFEA.ChIP             "TFEA.ChIP"             "1.14.0"
## tibble                "tibble"                "3.1.6"
## tidygraph             "tidygraph"             "1.2.0"
```

```
## tidyr          "tidyr"          "1.1.4"
## tidyselect     "tidyselect"     "1.1.1"
## tidytree       "tidytree"       "0.3.6"
## tidyverse      "tidyverse"      "1.3.1"
## timeDate       "timeDate"       "3043.102"
## tinytex        "tinytex"        "0.35"
## topGO          "topGO"          "2.46.0"
## treeio         "treeio"         "1.16.2"
## tweenr         "tweenr"         "1.0.2"
## tzdb           "tzdb"           "0.2.0"
## usethis        "usethis"        "2.1.3"
## utf8           "utf8"           "1.2.2"
## uuid           "uuid"           "1.0-3"
## vctrs          "vctrs"          "0.3.8"
## VennDiagram    "VennDiagram"    "1.7.1"
## viridis        "viridis"        "0.6.2"
## viridisLite    "viridisLite"    "0.4.0"
## visNetwork     "visNetwork"     "2.1.0"
## vroom          "vroom"          "1.5.7"
## waldo          "waldo"          "0.3.1"
## whisker        "whisker"        "0.4"
## withr          "withr"          "2.4.3"
## xfun           "xfun"           "0.28"
## XML            "XML"            "3.99-0.8"
## xml2           "xml2"           "1.3.2"
## xopen          "xopen"          "1.0.0"
## xtable         "xtable"         "1.8-4"
## XVector        "XVector"        "0.34.0"
## yaml           "yaml"           "2.2.1"
## yulab.utils    "yulab.utils"    "0.0.4"
## zip            "zip"            "2.2.0"
## zlibbioc       "zlibbioc"       "1.40.0"
## base           "base"           "4.1.2"
## boot           "boot"           "1.3-28"
## class          "class"          "7.3-19"
## cluster        "cluster"        "2.1.2"
## codetools      "codetools"      "0.2-18"
## compiler       "compiler"       "4.1.2"
## datasets       "datasets"       "4.1.2"
## foreign        "foreign"        "0.8-81"
## graphics       "graphics"       "4.1.2"
## grDevices      "grDevices"      "4.1.2"
## grid           "grid"           "4.1.2"
## KernSmooth     "KernSmooth"     "2.23-20"
## lattice        "lattice"        "0.20-45"
## MASS           "MASS"           "7.3-54"
## Matrix         "Matrix"         "1.3-4"
## methods        "methods"        "4.1.2"
## mgcv           "mgcv"           "1.8-38"
## nlme           "nlme"           "3.1-153"
## nnet           "nnet"           "7.3-16"
## parallel       "parallel"       "4.1.2"
```

```
## rpart                    "rpart"                   "4.1-15"
## spatial                  "spatial"                 "7.3-14"
## splines                  "splines"                 "4.1.2"
## stats                    "stats"                   "4.1.2"
## stats4                   "stats4"                  "4.1.2"
## survival                 "survival"                "3.2-13"
## tcltk                    "tcltk"                   "4.1.2"
## tools                    "tools"                   "4.1.2"
## utils                    "utils"                   "4.1.2"
```

```
R version 4.0.5 (2021-03-31)
Platform: x86_64-conda-linux-gnu (64-bit)
Running under: Red Hat Enterprise Linux

Matrix products: default
BLAS/LAPACK: /data/user/jfisher7/.conda/envs/SR_TAU_CELL/lib/
libopenblasp-r0.3.18.so

locale:
[1] C

attached base packages:
[1] stats     graphics  grDevices utils     datasets  methods   base

loaded via a namespace (and not attached):
[1] compiler_4.0.5
[1] "Listing packages"
                       Package                  Version
AnnotationDbi          "AnnotationDbi"          "1.52.0"
AnnotationHub          "AnnotationHub"          "2.22.1"
BH                     "BH"                     "1.78.0-0"
Biobase                "Biobase"                "2.50.0"
BiocFileCache          "BiocFileCache"          "1.14.0"
BiocGenerics           "BiocGenerics"           "0.36.1"
BiocManager            "BiocManager"            "1.30.16"
BiocParallel           "BiocParallel"           "1.24.1"
BiocVersion            "BiocVersion"            "3.12.0"
DBI                    "DBI"                    "1.1.2"
DO.db                  "DO.db"                  "2.9"
DOSE                   "DOSE"                   "3.16.0"
DT                     "DT"                     "0.20"
DelayedArray           "DelayedArray"           "0.16.3"
ExperimentHub          "ExperimentHub"          "1.16.1"
GO.db                  "GO.db"                  "3.12.1"
GOSemSim               "GOSemSim"               "2.16.1"
GSEABase               "GSEABase"               "1.52.1"
GenomeInfoDb           "GenomeInfoDb"           "1.26.7"
GenomeInfoDbData       "GenomeInfoDbData"       "1.2.4"
GenomicRanges          "GenomicRanges"          "1.42.0"
HDF5Array              "HDF5Array"              "1.18.1"
IRanges                "IRanges"                "2.24.1"
MASS                   "MASS"                   "7.3-55"
Matrix                 "Matrix"                 "1.4-0"
MatrixGenerics         "MatrixGenerics"         "1.2.1"
R.methodsS3            "R.methodsS3"            "1.8.1"
R.oo                   "R.oo"                   "1.24.0"
R.utils                "R.utils"                "2.11.0"
R6                     "R6"                     "2.5.1"
RColorBrewer           "RColorBrewer"           "1.1-2"
RCurl                  "RCurl"                  "1.98-1.5"
```

```
RSQLite                "RSQLite"                "2.2.9"
Rcpp                   "Rcpp"                   "1.0.8"
RcppArmadillo          "RcppArmadillo"          "0.10.8.1.0"
RcppEigen              "RcppEigen"              "0.3.3.9.1"
Rhdf5lib               "Rhdf5lib"               "1.12.1"
S4Vectors              "S4Vectors"              "0.28.1"
SummarizedExperiment   "SummarizedExperiment"   "1.20.0"
XML                    "XML"                    "3.99-0.8"
XVector                "XVector"                "0.30.0"
affy                   "affy"                   "1.68.0"
affyio                 "affyio"                 "1.60.0"
annotate               "annotate"               "1.68.0"
askpass                "askpass"                "1.1"
assertthat             "assertthat"             "0.2.1"
backports              "backports"              "1.4.1"
base                   "base"                   "4.0.5"
base64enc              "base64enc"              "0.1-3"
bit                    "bit"                    "4.0.4"
bit64                  "bit64"                  "4.0.5"
bitops                 "bitops"                 "1.0-7"
blob                   "blob"                   "1.2.2"
brio                   "brio"                   "1.1.3"
broom                  "broom"                  "0.7.12"
bslib                  "bslib"                  "0.3.1"
cachem                 "cachem"                 "1.0.6"
callr                  "callr"                  "3.7.0"
cellranger             "cellranger"             "1.1.0"
cli                    "cli"                    "3.1.1"
clipr                  "clipr"                  "0.7.1"
clusterProfiler        "clusterProfiler"        "3.18.1"
colorspace             "colorspace"             "2.0-2"
commonmark             "commonmark"             "1.7"
compiler               "compiler"               "4.0.5"
cowplot                "cowplot"                "1.1.1"
cpp11                  "cpp11"                  "0.4.2"
crayon                 "crayon"                 "1.4.2"
crosstalk              "crosstalk"              "1.2.0"
curl                   "curl"                   "4.3.2"
data.table             "data.table"             "1.14.2"
datasets               "datasets"               "4.0.5"
dbplyr                 "dbplyr"                 "2.1.1"
desc                   "desc"                   "1.4.0"
diffobj                "diffobj"                "0.3.5"
digest                 "digest"                 "0.6.29"
downloader             "downloader"             "0.4"
dplyr                  "dplyr"                  "1.0.7"
dtplyr                 "dtplyr"                 "1.2.1"
ellipsis               "ellipsis"               "0.3.2"
enrichplot             "enrichplot"             "1.10.2"
evaluate               "evaluate"               "0.14"
```

fansi	"fansi"	"1.0.2"
farver	"farver"	"2.1.0"
fastmap	"fastmap"	"1.1.0"
fastmatch	"fastmatch"	"1.1-3"
fgsea	"fgsea"	"1.16.0"
fontawesome	"fontawesome"	"0.2.2"
forcats	"forcats"	"0.5.1"
formatR	"formatR"	"1.11"
fs	"fs"	"1.5.2"
futile.logger	"futile.logger"	"1.4.3"
futile.options	"futile.options"	"1.0.1"
gargle	"gargle"	"1.2.0"
generics	"generics"	"0.1.2"
ggforce	"ggforce"	"0.3.3"
ggfun	"ggfun"	"0.0.5"
ggplot2	"ggplot2"	"3.3.5"
ggraph	"ggraph"	"2.0.5"
ggrepel	"ggrepel"	"0.9.1"
glue	"glue"	"1.6.1"
googledrive	"googledrive"	"2.0.0"
googlesheets4	"googlesheets4"	"1.0.0"
grDevices	"grDevices"	"4.0.5"
graph	"graph"	"1.68.0"
graphics	"graphics"	"4.0.5"
graphlayouts	"graphlayouts"	"0.8.0"
grid	"grid"	"4.0.5"
gridExtra	"gridExtra"	"2.3"
gtable	"gtable"	"0.3.0"
haven	"haven"	"2.4.3"
highr	"highr"	"0.9"
hms	"hms"	"1.1.1"
htmltools	"htmltools"	"0.5.2"
htmlwidgets	"htmlwidgets"	"1.5.4"
httpuv	"httpuv"	"1.6.5"
httr	"httr"	"1.4.2"
ids	"ids"	"1.0.1"
igraph	"igraph"	"1.2.11"
interactiveDisplayBase	"interactiveDisplayBase"	"1.28.0"
isoband	"isoband"	"0.2.5"
jquerylib	"jquerylib"	"0.1.4"
jsonlite	"jsonlite"	"1.7.3"
knitr	"knitr"	"1.37"
labeling	"labeling"	"0.4.2"
lambda.r	"lambda.r"	"1.2.4"
later	"later"	"1.3.0"
lattice	"lattice"	"0.20-45"
lazyeval	"lazyeval"	"0.2.2"
lifecycle	"lifecycle"	"1.0.1"
limma	"limma"	"3.46.0"
lubridate	"lubridate"	"1.8.0"

```
magrittr              "magrittr"              "2.0.2"
matrixStats           "matrixStats"           "0.61.0"
memoise               "memoise"               "2.0.1"
methods               "methods"               "4.0.5"
mgcv                  "mgcv"                  "1.8-38"
mime                  "mime"                  "0.12"
modelr                "modelr"                "0.1.8"
munsell               "munsell"               "0.5.0"
nlme                  "nlme"                  "3.1-155"
openssl               "openssl"               "1.4.6"
parallel              "parallel"              "4.0.5"
pillar                "pillar"                "1.7.0"
pkgconfig             "pkgconfig"             "2.0.3"
pkgload               "pkgload"               "1.2.4"
plogr                 "plogr"                 "0.2.0"
plyr                  "plyr"                  "1.8.6"
polyclip              "polyclip"              "1.10-0"
praise                "praise"                "1.0.0"
preprocessCore        "preprocessCore"        "1.52.1"
prettyunits           "prettyunits"           "1.1.1"
processx              "processx"              "3.5.2"
progress              "progress"              "1.2.2"
promises              "promises"              "1.2.0.1"
ps                    "ps"                    "1.6.0"
purrr                 "purrr"                 "0.3.4"
qvalue                "qvalue"                "2.22.0"
rappdirs              "rappdirs"              "0.3.3"
reactome.db           "reactome.db"           "1.74.0"
readr                 "readr"                 "2.1.2"
readxl                "readxl"                "1.3.1"
rematch               "rematch"               "1.0.1"
rematch2              "rematch2"              "2.1.2"
reprex                "reprex"                "2.0.1"
reshape2              "reshape2"              "1.4.4"
rhdf5                 "rhdf5"                 "2.34.0"
rhdf5filters          "rhdf5filters"          "1.2.1"
rlang                 "rlang"                 "1.0.0"
rmarkdown             "rmarkdown"             "2.11"
rprojroot             "rprojroot"             "2.0.2"
rstudioapi            "rstudioapi"            "0.13"
rvcheck               "rvcheck"               "0.2.1"
rvest                 "rvest"                 "1.0.2"
sass                  "sass"                  "0.4.0"
scales                "scales"                "1.1.1"
scatterpie            "scatterpie"            "0.1.7"
selectr               "selectr"               "0.4-2"
shadowtext            "shadowtext"            "0.1.1"
shiny                 "shiny"                 "1.7.1"
signatureSearch       "signatureSearch"       "1.4.6"
signatureSearchData   "signatureSearchData"   "1.4.0"
```

snow	"snow"	"0.4-4"
sourcetools	"sourcetools"	"0.1.7"
splines	"splines"	"4.0.5"
stats	"stats"	"4.0.5"
stats4	"stats4"	"4.0.5"
stringi	"stringi"	"1.7.6"
stringr	"stringr"	"1.4.0"
sys	"sys"	"3.4"
tcltk	"tcltk"	"4.0.5"
testthat	"testthat"	"3.1.2"
tibble	"tibble"	"3.1.6"
tidygraph	"tidygraph"	"1.2.0"
tidyr	"tidyr"	"1.2.0"
tidyselect	"tidyselect"	"1.1.1"
tidyverse	"tidyverse"	"1.3.1"
tinytex	"tinytex"	"0.36"
tools	"tools"	"4.0.5"
tweenr	"tweenr"	"1.0.2"
tzdb	"tzdb"	"0.2.0"
utf8	"utf8"	"1.2.2"
utils	"utils"	"4.0.5"
uuid	"uuid"	"1.0-3"
vctrs	"vctrs"	"0.3.8"
viridis	"viridis"	"0.6.2"
viridisLite	"viridisLite"	"0.4.0"
visNetwork	"visNetwork"	"2.1.0"
vroom	"vroom"	"1.5.7"
waldo	"waldo"	"0.3.1"
withr	"withr"	"2.4.3"
xfun	"xfun"	"0.29"
xml2	"xml2"	"1.3.3"
xtable	"xtable"	"1.8-4"
yaml	"yaml"	"2.2.2"
yulab.utils	"yulab.utils"	"0.0.4"
zlibbioc	"zlibbioc"	"1.36.0"

221221_rstudio_cancer_dr_info

Jennifer Fisher

2022-12-21

```
GitHub Repo:
Transfer_Leanring_R03

Docker:
rstudio_cancer_dr

Directory of operations:
/home/rstudio (Docker)
```

```
sessionInfo()
```

```
## R version 4.1.3 (2022-03-10)
## Platform: x86_64-pc-linux-gnu (64-bit)
## Running under: Ubuntu 20.04.4 LTS
##
## Matrix products: default
## BLAS/LAPACK: /usr/lib/x86_64-linux-gnu/openblas-pthread/libopenblasp-r0.3.8.so
##
## locale:
##  [1] LC_CTYPE=en_US.UTF-8       LC_NUMERIC=C
##  [3] LC_TIME=en_US.UTF-8        LC_COLLATE=en_US.UTF-8
##  [5] LC_MONETARY=en_US.UTF-8    LC_MESSAGES=en_US.UTF-8
##  [7] LC_PAPER=en_US.UTF-8       LC_NAME=C
##  [9] LC_ADDRESS=C               LC_TELEPHONE=C
## [11] LC_MEASUREMENT=en_US.UTF-8 LC_IDENTIFICATION=C
##
## attached base packages:
## [1] stats     graphics  grDevices utils     datasets  methods   base
##
## loaded via a namespace (and not attached):
##  [1] digest_0.6.29   R6_2.5.1        jsonlite_1.8.0  magrittr_2.0.3
##  [5] evaluate_0.16   stringi_1.7.8   cachem_1.0.6    rlang_1.0.6
##  [9] cli_3.4.1       rstudioapi_0.13 jquerylib_0.1.4 bslib_0.4.0
## [13] rmarkdown_2.16  tools_4.1.3     stringr_1.4.1   xfun_0.33
## [17] yaml_2.3.5      fastmap_1.1.0   compiler_4.1.3  htmltools_0.5.3
## [21] knitr_1.40      sass_0.4.2
```

```
# Listing packages
installed.packages()[,c(1,3)]
```

```
##                        Package                 Version
## abind                  "abind"                 "1.4-5"
## affy                   "affy"                  "1.72.0"
## affyio                 "affyio"                "1.64.0"
## amap                   "amap"                  "0.8-18"
## annotate               "annotate"              "1.72.0"
## AnnotationDbi          "AnnotationDbi"         "1.56.2"
## AnnotationFilter       "AnnotationFilter"      "1.18.0"
## AnnotationForge        "AnnotationForge"       "1.36.0"
## AnnotationHub          "AnnotationHub"         "3.2.2"
## AnVIL                  "AnVIL"                 "1.6.6"
## apcluster              "apcluster"             "1.4.10"
## ape                    "ape"                   "5.6-2"
## apeglm                 "apeglm"                "1.16.0"
## aplot                  "aplot"                 "0.1.7"
## ashr                   "ashr"                  "2.2-55"
## askpass                "askpass"               "1.1"
## assertthat             "assertthat"            "0.2.1"
## backports              "backports"             "1.4.1"
## base64                 "base64"                "2.0.1"
## base64enc              "base64enc"             "0.1-3"
## base64url              "base64url"             "1.4"
## bayestestR             "bayestestR"            "0.13.0"
## bbmle                  "bbmle"                 "1.0.25"
## bdsmatrix              "bdsmatrix"             "1.3-6"
## beanplot               "beanplot"              "1.3.1"
## BgeeDB                 "BgeeDB"                "2.20.1"
## BH                     "BH"                    "1.78.0-0"
## Biobase                "Biobase"               "2.54.0"
## BiocFileCache          "BiocFileCache"         "2.2.1"
## BiocGenerics           "BiocGenerics"          "0.40.0"
## BiocIO                 "BiocIO"                "1.4.0"
## BiocManager            "BiocManager"           "1.30.16"
## BiocParallel           "BiocParallel"          "1.28.3"
## BiocVersion            "BiocVersion"           "3.14.0"
## biomaRt                "biomaRt"               "2.50.3"
## Biostrings             "Biostrings"            "2.62.0"
## bit                    "bit"                   "4.0.4"
## bit64                  "bit64"                 "4.0.5"
## bitops                 "bitops"                "1.0-7"
## biwt                   "biwt"                  "1.0.1"
## blob                   "blob"                  "1.2.3"
## brew                   "brew"                  "1.0-7"
## brio                   "brio"                  "1.1.3"
## broom                  "broom"                 "1.0.1"
## broom.mixed            "broom.mixed"           "0.2.9.4"
## bslib                  "bslib"                 "0.4.0"
## bumphunter             "bumphunter"            "1.36.0"
## cachem                 "cachem"                "1.0.6"
## callr                  "callr"                 "3.7.0"
## car                    "car"                   "3.1-0"
## carData                "carData"               "3.0-5"
```

```
## caret                "caret"                "6.0-93"
## Category             "Category"             "2.60.0"
## caTools              "caTools"              "1.18.2"
## cellranger           "cellranger"           "1.1.0"
## ChemmineR            "ChemmineR"            "3.46.0"
## chron                "chron"                "2.3-57"
## circlize             "circlize"             "0.4.15"
## classInt             "classInt"             "0.4-7"
## cli                  "cli"                  "3.4.1"
## clipr                "clipr"                "0.8.0"
## clue                 "clue"                 "0.3-61"
## clusterProfiler      "clusterProfiler"      "4.2.2"
## coda                 "coda"                 "0.19-4"
## CoGAPS               "CoGAPS"               "3.14.0"
## cogena               "cogena"               "1.28.0"
## colorspace           "colorspace"           "2.0-3"
## combinat             "combinat"             "0.0-8"
## commonmark           "commonmark"           "1.8.0"
## ComplexHeatmap       "ComplexHeatmap"       "2.10.0"
## ComplexUpset         "ComplexUpset"         "1.3.3"
## conflicted           "conflicted"           "1.1.0"
## corrplot             "corrplot"             "0.92"
## cowplot              "cowplot"              "1.1.1"
## cpp11                "cpp11"                "0.4.2"
## crayon               "crayon"               "1.5.2"
## credentials          "credentials"          "1.3.2"
## crosstalk            "crosstalk"            "1.2.0"
## curl                 "curl"                 "4.3.2"
## customCMPdb          "customCMPdb"          "1.4.0"
## data.table           "data.table"           "1.14.2"
## datawizard           "datawizard"           "0.6.1"
## DBI                  "DBI"                  "1.1.3"
## dbplyr               "dbplyr"               "2.2.1"
## DelayedArray         "DelayedArray"         "0.20.0"
## DelayedMatrixStats   "DelayedMatrixStats"   "1.16.0"
## dendextend           "dendextend"           "1.16.0"
## DEoptimR             "DEoptimR"             "1.0-11"
## desc                 "desc"                 "1.4.1"
## DESeq2               "DESeq2"               "1.34.0"
## devtools             "devtools"             "2.4.4"
## dials                "dials"                "1.0.0"
## DiceDesign           "DiceDesign"           "1.9"
## diffobj              "diffobj"              "0.3.5"
## digest               "digest"               "0.6.29"
## discrim              "discrim"              "1.0.0"
## DO.db                "DO.db"                "2.9"
## docopt               "docopt"               "0.7.1"
## doParallel           "doParallel"           "1.0.17"
## doRNG                "doRNG"                "1.8.2"
## DOSE                 "DOSE"                 "3.20.1"
## dotwhisker           "dotwhisker"           "0.7.4"
## downlit              "downlit"              "0.4.2"
```

```
## downloader           "downloader"           "0.4"
## dplyr                "dplyr"                "1.0.10"
## drugbankR            "drugbankR"            "1.5"
## DT                   "DT"                   "0.25"
## dtplyr               "dtplyr"               "1.2.2"
## e1071                "e1071"                "1.7-11"
## earth                "earth"                "5.3.1"
## edgeR                "edgeR"                "3.36.0"
## ellipse              "ellipse"              "0.4.3"
## ellipsis             "ellipsis"             "0.3.2"
## emdbook              "emdbook"              "1.3.12"
## emmeans              "emmeans"              "1.8.1-1"
## enrichplot           "enrichplot"           "1.14.2"
## EnsDb.Hsapiens.v75   "EnsDb.Hsapiens.v75"   "2.99.0"
## ensembldb            "ensembldb"            "2.18.4"
## estimability         "estimability"         "1.4.1"
## etrunct              "etrunct"              "0.1"
## evaluate             "evaluate"             "0.16"
## ExperimentHub        "ExperimentHub"        "2.2.1"
## factoextra           "factoextra"           "1.0.7"
## FactoMineR           "FactoMineR"           "2.6"
## fansi                "fansi"                "1.0.3"
## farver               "farver"               "2.1.1"
## fastcluster          "fastcluster"          "1.2.3"
## fastmap              "fastmap"              "1.1.0"
## fastmatch            "fastmatch"            "1.1-3"
## fgsea                "fgsea"                "1.20.0"
## filelock             "filelock"             "1.0.2"
## flashClust           "flashClust"           "1.01-2"
## fmcsR                "fmcsR"                "1.36.0"
## fontawesome          "fontawesome"          "0.3.0"
## forcats              "forcats"              "0.5.2"
## foreach              "foreach"              "1.5.2"
## formatR              "formatR"              "1.12"
## Formula              "Formula"              "1.2-4"
## fs                   "fs"                   "1.5.2"
## furrr                "furrr"                "0.3.1"
## futile.logger        "futile.logger"        "1.4.3"
## futile.options       "futile.options"       "1.0.1"
## future               "future"               "1.28.0"
## future.apply         "future.apply"         "1.9.1"
## gargle               "gargle"               "1.2.1"
## gbm                  "gbm"                  "2.1.8.1"
## genefilter           "genefilter"           "1.76.0"
## geneplotter          "geneplotter"          "1.72.0"
## generics             "generics"             "0.1.3"
## GenomeInfoDb         "GenomeInfoDb"         "1.30.1"
## GenomeInfoDbData     "GenomeInfoDbData"     "1.2.7"
## GenomicAlignments    "GenomicAlignments"    "1.30.0"
## GenomicFeatures      "GenomicFeatures"      "1.46.5"
## GenomicRanges        "GenomicRanges"        "1.46.1"
## GEOquery             "GEOquery"             "2.62.2"
```

```
## gert                  "gert"                "1.5.0"
## GetoptLong            "GetoptLong"          "1.0.5"
## ggalluvial            "ggalluvial"          "0.12.3"
## ggdendro              "ggdendro"            "0.1.23"
## ggforce               "ggforce"             "0.3.4"
## ggfun                 "ggfun"               "0.0.7"
## ggplot2               "ggplot2"             "3.3.6"
## ggplotify             "ggplotify"           "0.1.0"
## ggpubr                "ggpubr"              "0.4.0"
## ggraph                "ggraph"              "2.0.6"
## ggrepel               "ggrepel"             "0.9.1"
## ggsci                 "ggsci"               "2.9"
## ggsignif              "ggsignif"            "0.6.3"
## ggstance              "ggstance"            "0.3.5"
## ggtree                "ggtree"              "3.2.1"
## gh                    "gh"                  "1.3.0"
## gitcreds              "gitcreds"            "0.1.1"
## glmnet                "glmnet"              "4.1-4"
## GlobalOptions         "GlobalOptions"       "0.1.2"
## globals               "globals"             "0.16.1"
## glue                  "glue"                "1.6.2"
## GO.db                 "GO.db"               "3.14.0"
## googledrive           "googledrive"         "2.0.0"
## googlesheets4         "googlesheets4"       "1.0.1"
## GOSemSim              "GOSemSim"            "2.20.0"
## GOstats               "GOstats"             "2.60.0"
## gower                 "gower"               "1.0.0"
## GPfit                 "GPfit"               "1.0-8"
## gplots                "gplots"              "3.1.3"
## gprofiler2            "gprofiler2"          "0.2.1"
## graph                 "graph"               "1.72.0"
## graphlayouts          "graphlayouts"        "0.8.2"
## gridBase              "gridBase"            "0.4-7"
## gridExtra             "gridExtra"           "2.3"
## gridGraphics          "gridGraphics"        "0.5-1"
## GSEABase              "GSEABase"            "1.56.0"
## gsubfn                "gsubfn"              "0.7"
## gtable                "gtable"              "0.3.1"
## gtools                "gtools"              "3.9.3"
## hardhat               "hardhat"             "1.2.0"
## hash                  "hash"                "2.2.6.2"
## haven                 "haven"               "2.5.1"
## HDF5Array             "HDF5Array"           "1.22.1"
## here                  "here"                "1.0.1"
## hexbin                "hexbin"              "1.28.2"
## highr                 "highr"               "0.9"
## hms                   "hms"                 "1.1.2"
## htmltools             "htmltools"           "0.5.3"
## htmlwidgets           "htmlwidgets"         "1.5.4"
## httpuv                "httpuv"              "1.6.6"
## httr                  "httr"                "1.4.4"
## ids                   "ids"                 "1.0.1"
```

```
## igraph                 "igraph"                 "1.3.5"
## illuminaio             "illuminaio"             "0.36.0"
## infer                  "infer"                  "1.0.3"
## ini                    "ini"                    "0.3.1"
## insight                "insight"                "0.18.4"
## interactiveDisplayBase "interactiveDisplayBase" "1.32.0"
## inum                   "inum"                   "1.0-4"
## invgamma               "invgamma"               "1.1"
## ipred                  "ipred"                  "0.9-13"
## IRanges                "IRanges"                "2.28.0"
## IRdisplay              "IRdisplay"              "1.1"
## IRkernel               "IRkernel"               "1.3"
## irlba                  "irlba"                  "2.3.5"
## isoband                "isoband"                "0.2.5"
## iterators              "iterators"              "1.0.14"
## jquerylib              "jquerylib"              "0.1.4"
## jsonlite               "jsonlite"               "1.8.0"
## KEGGREST               "KEGGREST"               "1.34.0"
## kernlab                "kernlab"                "0.9-31"
## klaR                   "klaR"                   "1.7-1"
## knitr                  "knitr"                  "1.40"
## kohonen                "kohonen"                "3.0.11"
## labeling               "labeling"               "0.4.2"
## labelled               "labelled"               "2.10.0"
## lambda.r               "lambda.r"               "1.2.4"
## later                  "later"                  "1.3.0"
## lava                   "lava"                   "1.6.10"
## lazyeval               "lazyeval"               "0.2.2"
## leaps                  "leaps"                  "3.1"
## lhs                    "lhs"                    "1.1.5"
## libcoin                "libcoin"                "1.0-9"
## LiblineaR              "LiblineaR"              "2.10-12"
## lifecycle              "lifecycle"              "1.0.2"
## limma                  "limma"                  "3.50.3"
## listenv                "listenv"                "0.8.0"
## littler                "littler"                "0.3.15"
## lme4                   "lme4"                   "1.1-30"
## locfit                 "locfit"                 "1.5-9.6"
## lubridate              "lubridate"              "1.8.0"
## magrittr               "magrittr"               "2.0.3"
## maptools               "maptools"               "1.1-4"
## margins                "margins"                "0.3.26"
## markdown               "markdown"               "1.4"
## mashr                  "mashr"                  "0.2.57"
## MASS                   "MASS"                   "7.3-58.1"
## Matrix                 "Matrix"                 "1.5-1"
## MatrixGenerics         "MatrixGenerics"         "1.6.0"
## MatrixModels           "MatrixModels"           "0.5-1"
## matrixStats            "matrixStats"            "0.62.0"
## mclust                 "mclust"                 "5.4.10"
## memoise                "memoise"                "2.0.1"
## mime                   "mime"                   "0.12"
```

```
## minfi                "minfi"                "1.40.0"
## miniUI               "miniUI"               "0.1.1.1"
## minqa                "minqa"                "1.2.4"
## mixsqp               "mixsqp"               "0.3-43"
## modeldata            "modeldata"            "1.0.1"
## ModelMetrics         "ModelMetrics"         "1.2.2.2"
## modelr               "modelr"               "0.1.9"
## multcompView         "multcompView"         "0.1-8"
## multtest             "multtest"             "2.50.0"
## munsell              "munsell"              "0.5.0"
## mvtnorm              "mvtnorm"              "1.1-3"
## naivebayes           "naivebayes"           "0.9.7"
## netZooR              "netZooR"              "1.1.15"
## nloptr               "nloptr"               "2.0.3"
## NLP                  "NLP"                  "0.2-1"
## NMF                  "NMF"                  "0.24.0"
## nnet                 "nnet"                 "7.3-17"
## nor1mix              "nor1mix"              "1.3-0"
## numDeriv             "numDeriv"             "2016.8-1.1"
## openssl              "openssl"              "2.0.3"
## org.Hs.eg.db         "org.Hs.eg.db"         "3.14.0"
## pamr                 "pamr"                 "1.56.1"
## pandaR               "pandaR"               "1.26.0"
## parallelly           "parallelly"           "1.32.1"
## parameters           "parameters"           "0.18.2"
## parsnip              "parsnip"              "1.0.1"
## partykit             "partykit"             "1.2-16"
## pasilla              "pasilla"              "1.22.0"
## patchwork            "patchwork"            "1.1.2"
## pbdZMQ               "pbdZMQ"               "0.3-7"
## pbkrtest             "pbkrtest"             "0.5.1"
## penalized            "penalized"            "0.9-52"
## permute              "permute"              "0.9-7"
## pheatmap             "pheatmap"             "1.0.12"
## pillar               "pillar"               "1.8.1"
## pkgbuild             "pkgbuild"             "1.3.1"
## pkgconfig            "pkgconfig"            "2.0.3"
## pkgdown              "pkgdown"              "2.0.6"
## pkgload              "pkgload"              "1.3.0"
## pkgmaker             "pkgmaker"             "0.32.2"
## PLIER                "PLIER"                "0.99.0"
## plogr                "plogr"                "0.2.0"
## plotly               "plotly"               "4.10.0"
## plotmo               "plotmo"               "3.6.2"
## plotrix              "plotrix"              "3.8-2"
## plyr                 "plyr"                 "1.8.7"
## png                  "png"                  "0.1-7"
## polyclip             "polyclip"             "1.10-0"
## polynom              "polynom"              "1.4-1"
## praise               "praise"               "1.0.0"
## prediction           "prediction"           "0.3.14"
## preprocessCore       "preprocessCore"       "1.56.0"
```

```
## prettyunits           "prettyunits"           "1.1.1"
## pROC                  "pROC"                  "1.18.0"
## processx              "processx"              "3.5.2"
## prodlim               "prodlim"               "2019.11.13"
## profvis               "profvis"               "0.3.7"
## progress              "progress"              "1.2.2"
## progressr             "progressr"             "0.11.0"
## projectR              "projectR"              "1.10.0"
## ProliferativeIndex    "ProliferativeIndex"    "1.0.1"
## promises              "promises"              "1.2.0.1"
## ProtGenerics          "ProtGenerics"          "1.26.0"
## proto                 "proto"                 "1.0.0"
## proxy                 "proxy"                 "0.4-27"
## ps                    "ps"                    "1.6.0"
## purrr                 "purrr"                 "0.3.4"
## quadprog              "quadprog"              "1.5-8"
## quantreg              "quantreg"              "5.94"
## quantro               "quantro"               "1.28.0"
## questionr             "questionr"             "0.7.7"
## qvalue                "qvalue"                "2.26.0"
## R.cache               "R.cache"               "0.16.0"
## R.methodsS3           "R.methodsS3"           "1.8.2"
## R.oo                  "R.oo"                  "1.25.0"
## R.utils               "R.utils"               "2.12.0"
## R6                    "R6"                    "2.5.1"
## ragg                  "ragg"                  "1.2.2"
## randomForest          "randomForest"          "4.7-1.1"
## ranger                "ranger"                "0.14.1"
## rapiclient            "rapiclient"            "0.1.3"
## rappdirs              "rappdirs"              "0.3.3"
## RBGL                  "RBGL"                  "1.70.0"
## rcmdcheck             "rcmdcheck"             "1.4.0"
## RColorBrewer          "RColorBrewer"          "1.1-3"
## Rcpp                  "Rcpp"                  "1.0.9"
## RcppArmadillo         "RcppArmadillo"         "0.11.2.4.0"
## RcppEigen             "RcppEigen"             "0.3.3.9.2"
## RcppGSL               "RcppGSL"               "0.3.11"
## RcppNumerical         "RcppNumerical"         "0.4-0"
## RcppTOML              "RcppTOML"              "0.1.7"
## RCurl                 "RCurl"                 "1.98-1.8"
## RCy3                  "RCy3"                  "2.14.2"
## reactome.db           "reactome.db"           "1.77.0"
## readr                 "readr"                 "2.1.2"
## readxl                "readxl"                "1.4.1"
## recipes               "recipes"               "1.0.1"
## recount3              "recount3"              "1.4.0"
## registry              "registry"              "0.5-1"
## rematch               "rematch"               "1.0.1"
## rematch2              "rematch2"              "2.1.2"
## remotes               "remotes"               "2.4.2"
## repr                  "repr"                  "1.1.4"
## reprex                "reprex"                "2.0.2"
```

```
## reshape               "reshape"               "0.8.9"
## reshape2              "reshape2"              "1.4.4"
## restfulr              "restfulr"              "0.0.15"
## reticulate            "reticulate"            "1.26"
## Rgraphviz             "Rgraphviz"             "2.38.0"
## rhdf5                 "rhdf5"                 "2.38.1"
## rhdf5filters          "rhdf5filters"          "1.6.0"
## Rhdf5lib              "Rhdf5lib"              "1.16.0"
## Rhtslib               "Rhtslib"               "1.26.0"
## rJava                 "rJava"                 "1.0-6"
## rjson                 "rjson"                 "0.2.21"
## RJSONIO               "RJSONIO"               "1.3-1.6"
## rlang                 "rlang"                 "1.0.6"
## rmarkdown             "rmarkdown"             "2.16"
## rmeta                 "rmeta"                 "3.0"
## rngtools              "rngtools"              "1.5.2"
## robustbase            "robustbase"            "0.95-0"
## ROCR                  "ROCR"                  "1.0-11"
## roxygen2              "roxygen2"              "7.2.1"
## rpart                 "rpart"                 "4.1.16"
## rprojroot             "rprojroot"             "2.0.3"
## rrvgo                 "rrvgo"                 "1.6.0"
## rsample               "rsample"               "1.1.0"
## Rsamtools             "Rsamtools"             "2.10.0"
## RSQLite               "RSQLite"               "2.2.17"
## rstatix               "rstatix"               "0.7.0"
## rstudioapi            "rstudioapi"            "0.13"
## rsvd                  "rsvd"                  "1.0.5"
## rsvg                  "rsvg"                  "2.3.1"
## rtracklayer           "rtracklayer"           "1.54.0"
## RUnit                 "RUnit"                 "0.4.32"
## rversions             "rversions"             "2.1.1"
## rvest                 "rvest"                 "1.0.3"
## RWeka                 "RWeka"                 "0.4-44"
## RWekajars             "RWekajars"             "3.9.3-2"
## S4Vectors             "S4Vectors"             "0.32.4"
## sass                  "sass"                  "0.4.2"
## scales                "scales"                "1.2.1"
## scatterpie            "scatterpie"            "0.1.8"
## scatterplot3d         "scatterplot3d"         "0.3-42"
## scrime                "scrime"                "1.3.5"
## selectr               "selectr"               "0.4-2"
## sessioninfo           "sessioninfo"           "1.2.2"
## shadowtext            "shadowtext"            "0.1.2"
## shape                 "shape"                 "1.4.6"
## shiny                 "shiny"                 "1.7.2"
## siggenes              "siggenes"              "1.68.0"
## signatureSearch       "signatureSearch"       "1.8.2"
## signatureSearchData   "signatureSearchData"   "1.8.4"
## SingleCellExperiment  "SingleCellExperiment"  "1.16.0"
## skimr                 "skimr"                 "2.1.4"
## slam                  "slam"                  "0.1-50"
```

```
## slider                  "slider"                  "0.2.2"
## snow                    "snow"                    "0.4-4"
## softImpute              "softImpute"              "1.4-1"
## sourcetools             "sourcetools"             "0.1.7"
## sp                      "sp"                      "1.5-0"
## SparseM                 "SparseM"                 "1.81"
## sparseMatrixStats       "sparseMatrixStats"       "1.6.0"
## sqldf                   "sqldf"                   "0.4-11"
## SQUAREM                 "SQUAREM"                 "2021.1"
## STRINGdb                "STRINGdb"                "2.6.5"
## stringi                 "stringi"                 "1.7.8"
## stringr                 "stringr"                 "1.4.1"
## styler                  "styler"                  "1.7.0"
## SummarizedExperiment    "SummarizedExperiment"    "1.24.0"
## sys                     "sys"                     "3.4"
## systemfonts             "systemfonts"             "1.0.4"
## TeachingDemos           "TeachingDemos"           "2.12"
## testthat                "testthat"                "3.1.4"
## textshaping             "textshaping"             "0.3.6"
## TFEA.ChIP               "TFEA.ChIP"               "1.14.0"
## tibble                  "tibble"                  "3.1.8"
## tidygraph               "tidygraph"               "1.2.2"
## tidymodels              "tidymodels"              "1.0.0"
## tidyr                   "tidyr"                   "1.2.1"
## tidyselect              "tidyselect"              "1.1.2"
## tidytree                "tidytree"                "0.4.1"
## tidyverse               "tidyverse"               "1.3.2"
## timeDate                "timeDate"                "4021.104"
## tinytex                 "tinytex"                 "0.41"
## tm                      "tm"                      "0.7-8"
## topGO                   "topGO"                   "2.46.0"
## treeio                  "treeio"                  "1.18.1"
## treemap                 "treemap"                 "2.4-3"
## truncnorm               "truncnorm"               "1.0-8"
## tune                    "tune"                    "1.0.0"
## tweenr                  "tweenr"                  "2.0.2"
## tzdb                    "tzdb"                    "0.3.0"
## uchardet                "uchardet"                "1.1.0"
## urlchecker              "urlchecker"              "1.0.1"
## usethis                 "usethis"                 "2.1.6"
## utf8                    "utf8"                    "1.2.2"
## uuid                    "uuid"                    "1.1-0"
## vctrs                   "vctrs"                   "0.4.2"
## vegan                   "vegan"                   "2.6-2"
## VennDiagram             "VennDiagram"             "1.7.3"
## viridis                 "viridis"                 "0.6.2"
## viridisLite             "viridisLite"             "0.4.1"
## visNetwork              "visNetwork"              "2.1.2"
## vroom                   "vroom"                   "1.5.7"
## waldo                   "waldo"                   "0.4.0"
## warp                    "warp"                    "0.2.0"
## whisker                 "whisker"                 "0.4"
```

```
## withr          "withr"          "2.5.0"
## wordcloud      "wordcloud"      "2.6"
## workflows      "workflows"      "1.1.0"
## workflowsets   "workflowsets"   "1.0.0"
## xfun           "xfun"           "0.33"
## xgboost        "xgboost"        "1.6.0.1"
## XML            "XML"            "3.99-0.10"
## xml2           "xml2"           "1.3.3"
## xopen          "xopen"          "1.0.0"
## xtable         "xtable"         "1.8-4"
## XVector        "XVector"        "0.34.0"
## yaml           "yaml"           "2.3.5"
## yardstick      "yardstick"      "1.1.0"
## yarn           "yarn"           "1.20.0"
## yulab.utils    "yulab.utils"    "0.0.5"
## zip            "zip"            "2.2.0"
## zlibbioc       "zlibbioc"       "1.40.0"
## base           "base"           "4.1.3"
## boot           "boot"           "1.3-28"
## class          "class"          "7.3-20"
## cluster        "cluster"        "2.1.2"
## codetools      "codetools"      "0.2-18"
## compiler       "compiler"       "4.1.3"
## datasets       "datasets"       "4.1.3"
## foreign        "foreign"        "0.8-82"
## graphics       "graphics"       "4.1.3"
## grDevices      "grDevices"      "4.1.3"
## grid           "grid"           "4.1.3"
## KernSmooth     "KernSmooth"     "2.23-20"
## lattice        "lattice"        "0.20-45"
## MASS           "MASS"           "7.3-55"
## Matrix         "Matrix"         "1.4-0"
## methods        "methods"        "4.1.3"
## mgcv           "mgcv"           "1.8-39"
## nlme           "nlme"           "3.1-155"
## nnet           "nnet"           "7.3-17"
## parallel       "parallel"       "4.1.3"
## rpart          "rpart"          "4.1.16"
## spatial        "spatial"        "7.3-15"
## splines        "splines"        "4.1.3"
## stats          "stats"          "4.1.3"
## stats4         "stats4"         "4.1.3"
## survival       "survival"       "3.3-1"
## tcltk          "tcltk"          "4.1.3"
## tools          "tools"          "4.1.3"
## utils          "utils"          "4.1.3"
```
